# An adverse outcome pathway for parkinsonian motor deficits associated with mitochondrial complex I inhibition

**DOI:** 10.1007/s00204-017-2133-4

**Published:** 2017-12-05

**Authors:** Andrea Terron, Anna Bal-Price, Alicia Paini, Florianne Monnet-Tschudi, Susanne Hougaard Bennekou, EFSA WG EPI1 Members, Karine Angeli, Ellen Fritsche, Alberto Mantovani, Barbara Viviani, Marcel Leist, Stefan Schildknecht

**Affiliations:** 1 European Food Safety Authority, Parma, Italy; 2 European Commission Joint Research Centre, Ispra, Italy; 3 University of Lausanne and SCAHT, Lausanne, Switzerland; 4 The Danish Environmental Protection Agency, Copenhagen, Denmark; 5 In Vitro Toxicology and Biomedicine, Department of Biology, University of Konstanz, Universitätsstr. 10, PO Box M657, 78457 Konstanz, Germany

**Keywords:** Adverse outcome pathway, Mitochondrial complex I inhibitor, Parkinson’s disease, Pesticide exposure, Rotenone, MPTP, Regulatory decision-making

## Abstract

Epidemiological studies have observed an association between pesticide exposure and the development of Parkinson’s disease, but have not established causality. The concept of an adverse outcome pathway (AOP) has been developed as a framework for the organization of available information linking the modulation of a molecular target [molecular initiating event (MIE)], via a sequence of essential biological key events (KEs), with an adverse outcome (AO). Here, we present an AOP covering the toxicological pathways that link the binding of an inhibitor to mitochondrial complex I (i.e., the MIE) with the onset of parkinsonian motor deficits (i.e., the AO). This AOP was developed according to the Organisation for Economic Co-operation and Development guidelines and uploaded to the AOP database. The KEs linking complex I inhibition to parkinsonian motor deficits are mitochondrial dysfunction, impaired proteostasis, neuroinflammation, and the degeneration of dopaminergic neurons of the *substantia nigra*. These KEs, by convention, were linearly organized. However, there was also evidence of additional feed-forward connections and shortcuts between the KEs, possibly depending on the intensity of the insult and the model system applied. The present AOP demonstrates mechanistic plausibility for epidemiological observations on a relationship between pesticide exposure and an elevated risk for Parkinson’s disease development.

## Introduction

Pesticides such as dichlorodiphenyltrichloroethane (DDT), dieldrin, paraquat, and rotenone have been considered as potential contributing factors to the development of Parkinson’s disease (PD) (Baltazar et al. 2014; Sandström et al. 2014). Concerns about the contribution of environmental agents to parkinsonian disorders have led to epidemiological studies examining an association between human exposure to pesticides and the development of PD. Meta-analyses of these epidemiological studies confirmed a significant association between pesticide exposure and PD. Such observations are difficult to integrate into regulatory risk assessments, as exposure is currently evaluated retrospectively and indirectly in the vast majority of epidemiological studies. Therefore, these studies do not allow the identification of causal relationships (Breckenridge et al. 2016; Hernández et al. 2016; Van Maele-Fabry et al. 2012). Moreover, epidemiological observations usually do not provide a plausible link to molecular processes known to be associated with PD pathogenesis.

The adverse outcome pathway (AOP) concept organizes heterogeneous biological and toxicological data to provide information on possible sequences of events across multiple levels of biological organization (Bal-Price et al. 2017; Villeneuve et al. 2014a, b; Leist et al. 2017). An AOP represents a linear sequence of key events (KEs) causally connected through key event relationships (KERs) that provide a plausible link between a molecular initiating event (MIE) and an adverse outcome (AO). The MIE is defined as the first specific modification of a biological target at the molecular level by a chemical that can trigger the subsequent events, leading to pathology (i.e., the AO). Notably, AOPs of regulatory significance describe a sequence of biological processes (biochemical, cellular, physiological) and not the effects (mode of action) of a specific compound (AOPs are “compound agnostic”). The most important implication of this characteristic is that they do not describe or take into account toxicokinetics. The latter cannot be described in a generic way but is inseparably coupled to the molecular identity of a toxicant. Essential criteria for the overall evaluation of AOPs is the presence of a solid basis for the (1) essentiality of KEs, (2) the biological plausibility and empirical support for KERs. The empirical support for the KER is indeed complementing the biological plausibility for the KER and the essentiality of the KEs. These principles are described in the Organisation for Economic Co-operation and Development (OECD) guidance document on the development of an AOP (http://www.oecd-ilibrary.org). The ratings represent a comparative measure of the degree of confidence in the supporting weight of evidence, based on acquired collective experience. In the future, the AOP framework could contribute to an integrated approach to testing and assessment (IATA) that includes absorption, distribution, metabolism, excretion (ADME) information as well as quantification of effective threshold concentrations.

**Fig. 1:**
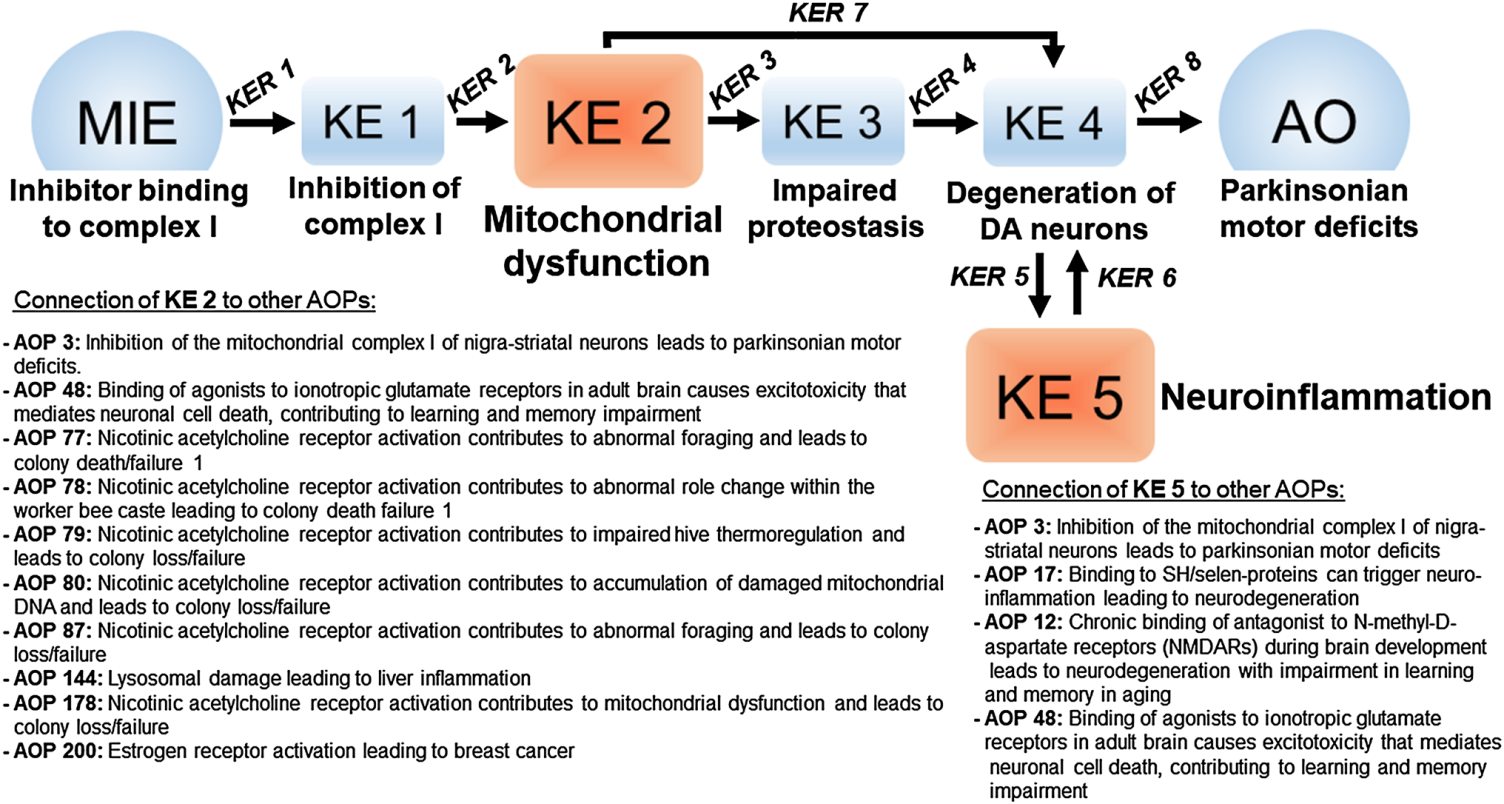
Schematic overview on the adverse outcome pathway (AOP) for the development of parkinsonian motor deficits by inhibitor binding to mitochondrial complex I. The AOP is initiated by binding of an inhibitor to mitochondrial complex I as the molecular initiating event (MIE), leading to the activation of a series of key events (KEs) that cover various levels of biological organization. Parkinsonian motor deficits were selected as the adverse outcome (AO) of the present AOP, based on its relevance in risk assessment. Key event relationships (KER) (indicated by arrows) represent the available experimental evidence in the literature, illustrating a quantitative relationship between a KE and its corresponding downstream KE. *Overlap with other AOPs*: overlap of KEs integrated in the AOP “Inhibition of mitochondrial complex I of nigro-striatal neurons leads to parkinsonian motor deficits” with KEs of other AOPs of the AOP-Wiki (https://aopwiki.org) was examined in October 2017. Mitochondrial dysfunction (KE 2) is part of 9, while Neuroinflammation (KE 5) is part of 3 other AOPs in the AOP Wiki in different stages of development

In a meta-analysis by Tanner et al. (Tanner et al. 2011), pesticides were classified by their presumed mechanism and not only by their chemical class. This study allowed the identification of significant associations between the inhibition of mitochondrial complex I and a parkinsonian phenotype. A causal role of complex I inhibition in the development of a parkinsonian phenotype is supported by broad evidence, and over the past 30 years, the complex I inhibitors rotenone and MPP^+^ emerged as the most widely applied experimental toxicants in PD research (Schildknecht 2017). On this basis, we constructed an AOP that describes the link between the inhibition of mitochondrial complex I and the manifestation of parkinsonian motor deficits. The full AOP (Fig. 1) can be found in the AOP Wiki (https://aopwiki.org/aops/3), an AOP platform established as part of the 2012 OECD AOP development work plan.

## Key events

Key events, essential for the progression of the response evoked by inhibitor binding to complex I (MIE) towards the AO, were assessed on their essentiality, based on the present knowledge on how a KE works, on the availability of robust detection methods (Table 1) and on evidence in the literature, indicating that experimental inhibition of a KE reduces or abolishes downstream KE activation. Weight of evidence analysis for the rating of KE essentiality is summarized in Table 2.

**Table 1:** Description of the individual key events

Key event	How this KE works	Analytical detection	Remarks and references
MIE: Inhibitor binding to complex I	Complex I (NADH-ubiquinone oxidoreductase) acts as the initial acceptor of electrons from NADH and orchestrates the electron transfer via flavin mononucleotide (FMN) and eight iron–sulfur (Fe–S)-clusters onto ubiquinone [1]. Rotenoids, piercidines, myxothiazoles, and vanilloids were identified as complex I inhibitors. It is assumed that electron transfer between the terminal Fe–S-cluster N_2_ and ubiquinone or the semiquinone dismutation step is affected by the inhibitors [2]. The exact inhibitor binding site is controversially discussed. It is currently assumed that all hydrophobic inhibitors target a common binding domain within the ubiquinone binding pocket [3–6]	Quantitative autoradiography or radioligand binding assay: displacement of [^3^H]-dihydrorotenone by an inhibitor as indicator for binding to the rotenone-binding site. Application in vitro; ex vivo (histologically or biochemically) [7–9] Complex I enzymatic activity assays of KE1 are used as indirect measures of binding: oxidation of NADH to NAD^+^, reduction of detection dye (e.g., nitroblue tetrazolium) [3, 10]	Complex I is a very large, multiprotein complex with 14 core subunits, highly conserved from bacteria to man. The total number of subunits varies between species (mammals = 44–46) Most activity assays have large uncertainties [1] Sharma et al. (2009), [2] Okun et al. (1999), [3] Friedrich et al. (1994), [4] Degli Esposti (1998), [5] Ino et al. (2003), [6] Degli-Esposito et al. (1996), [7] Greenamyre et al. (1992), [8] Higgins and Greenamyre (1996), [9] Talpade et al. (2000), [10] Höllerhage et al. (2009)
KE 1: Inhibition of complex I	Complex I accomplishes the coordinated reduction of ubiquinone to ubiquinol with electrons provided by the Krebs cycle in the form of NADH [1]. Electron transfer through complex I is coupled to H^+^ translocation out of the mitochondrial matrix [2, 3]. Inhibition of complex I impairs the generation of a proton gradient along the inner mitochondrial membrane and this negatively affects mitochondrial ATP generation. Accidental electron transfer from highly reduced Fe-clusters of complex I onto O_2_ leads to the formation of superoxide (^•^O_2_^−^) [4, 5]. Accumulation of NADH leads to a feedback inhibition of key dehydrogenase enzymes of the Krebs cycle	Direct methods Complex I activity assay (forward): photometric detection of NADH consumption and electron acceptor reduction (e.g., nitroblue tetrazolium) [6, 7] Complex I activity assay (reverse): electron entry via complex II (succinate). Photometric detection of NADH [8] Indirect methods Under conditions favoring dependence on complex I O_2_ consumption: isolated mitochondria, intact cells Enzymatic detection of cellular ATP [8–13]	[1] Lenaz et al. (2004), [2] Brandt (1997), [3] Treberg and Brand (2011), [4] Spinazzi et al. (2012), [5] Long et al. (2009), [6] Ernster and Lee (1967), [7] Kirby et al. (2007), [8] Höllerhage et al. (2009), [9] Wang and Wolfbeis (2014), [10] Salabei et al. (2014), [11] Nguyen et al. (1988), [12] Leist et al. (1997)
KE 2: Mitochondrial dysfunction	Mitochondrial dysfunction is characterized by one or more of the following features: inhibition of the respiratory chain, loss of the mitochondrial transmembrane potential, decline in ATP production; elevated formation of ROS, disturbances in mitochondrial Ca^2+^ handling, deregulation of fission/fusion processes, opening of the mitochondrial permeability transition pore, release of pro-apoptotic factors (e.g., cytochrome *c*, apoptosis inducing factor). The state “dysfunction” is context-dependent. The individual features of dysfunction are related to one another by feed-forward loops [1–4]	Detection of oxygen consumption Assessment of mitochondrial membrane potential by fluorescent dyes that accumulate in mitochondria with intact membrane potential Detection of mitochondrial permeability transition pore opening by analysis of, e.g., cytochrome *c*, adenylate kinase, apoptosis inducing factor [5–10] Assessment of mitochondrial DNA damage [11] Detection of reactive oxygen species (ROS) by fluorescent dyes interacting with free radicals [12–15]	Different measures of mitochondrial dysfunction, different sensitivities. Not all features may be observed to the same degree. It is not clear which aspect of mitochondrial dysfunction triggers KE 3 and KE 4 [1] Lin and Beal (2006), [2] Graier et al. (2007), [3] Braun (2012), [4] Correia et al. (2012), [5] Hafner et al. (1990), [6] Ciapaite et al. (2005), [7] Petronilli et al. (1999), [8] Barrientos and Moraes (1999), [9] Llaudet et al. (2005), [10] Lemasters et al. (2009), [11] Sanders et al. (2014), [12] Grivennikova and Vinogradov (2006), [13] McCord and Fridovich (1968), [14] Zhou et al. (1997), [15] Ruch et al. (1983)
KE 3: Impaired proteostasis	Proteostasis describes a coordinated balance between the synthesis, modification, transport, and degradation of proteins in a cell. Disturbances in proteostasis can lead to a loss of the genuine function of a protein or to the gain of undesired properties. Two major controllers of proteostasis are protein degradation and cellular transport mechanisms. The two major degradation systems in a cell are the [1–3] Ubiquitin proteasomal system (UPS) [4–9], and the autophagy–lysosomal pathway (ALP); responsible for the degradation of proteins, protein aggregates, or even organelles. Disturbed protein degradation or trafficking often leads to inappropriate protein accumulation in cells, ranging from protein aggregates (e.g., alpha synuclein) to whole mitochondria [10–20]	Ubiquitin proteasomal system (UPS) Detection of ubiquitinated proteins by western blot UPS activity assay: degradation of fluorigenic substrates can be used to quantify enyzmatic UPS activity Staining aggregation of exemplary proteins (e.g., alpha synuclein) as readout for impaired protein degradation [21–24] Autophagy–lysosomal pathway (ALP) Staining of lysosomes as ALP marker Detection of autophagic flux: ^14^C labeled intracellular proteins; detection of ^14^C in supernatant over time Assessment of LC3-I to LC-3 II conversion: LC3-II (post translational modification) correlates with the number of autophagosomes [25–29]	Disturbed proteostasis is a broad KE, comprising various cellular reactions. As in several degenerative states, multiple reactions converge on a relatively homogenous system state. Here, it is the formation of protein precipitates and of intermediates to this state, and this is widely accepted as KE in parkinsonian pathology [1] Lee et al. (2012), [2] Korolchuk et al. (2010), [3] Kroemer et al. (2010), [4] Ciechanover (1998), [5] Ciechanover and Brundin (2003), [6] Li et al. (1997), [7] Spillantini et al. (1997), [8] Sulzer and Zecca (2000), [9] McNaught and Jenner (2001a), [10] Kuma et al. (2004), [11] Cuervo (2004), [12] Mizushima et al. (2008), [13] Bartels et al. (2011), [14] Bellucci et al. (2012), [15] Shacka et al. (2008), [16] Pivtoraiko et al. (2009), [17] Chartier-Harlin et al. (2004), [18] Kitada et al. (1998), [19] Leroy et al. (1998), [20] Plowey et al. (2008), [21] Bence et al. (2001), [22] Kisselev and Goldberg (2005), [23] Rideout et al. (2001), [24] Ortega and Lucas (2014), [25] Klionsky et al. (2008), [26] Munafó and Colombo (2002), [27] Rajapakshe et al. (2015), [28] Kadowaki and Karim (2009), [29] Bauvy et al. (2009)
KE 4: DA neurodegeneration in the nigrostriatum	Neurons of the substantia nigra project into the striatum to release dopamine. In the striatum, DA has an excitatory (D1 receptors) and inhibitory (D2 receptors) influence on GABAergic striatal interneurons; DA augments (by both pathways) the thalamic output to the motor cortex A decline of striatal DA, therefore, leads to a decreased thamalic input to the cortex from the basal ganglia motor control loop. In idiopathic and genetic forms of Parkinson’s disease, or after exposure to toxicants such as MPTP, a preferential degeneration of nigrostriatal DA neurons is observed [1–4]	Labeling/expression levels of DA markers [tyrosine hydroxylase (TH), DA transporter (DAT), vesicular monoamine transporter (VMAT-2)] by western blot, immunocytochemistry [5] Counting of TH, DAT, VMAT-positive neurons, or of dopaminergic terminals in the striatum [6–10] Detection of DA, and its degradation products DOPAC (3,4-dihydroxyphenylacetic acid) and HVA (homovanillic acid) by HPLC, mass spectrometry [11] ^18^F-Dopa-positron emission tomography (PET) based quantification of DA-transporters (DAT, VMAT-2) [12, 13]	Anatomy and function of the nigrostriatal system is similar in mammals. Evidence on this KE is particularly broad and solid across multiple situations, models and species. However, standard histology and standard 28/90-day studies do not measure this KE [1] Fujita et al. (2008), [2] Obeso et al. (2008a), [3] Obeso et al. (2008b), [4] Blandini et al. (2000), [5] Schmued et al. (1997), [6] Betarbet et al. (2000), [7] Fetissov and Marsais (1999), [8] Dauer and Przedborski (2003), [9] Hirata et al. (2008), [10] Tong et al. (2011), [11] Fornai et al. (2003), [12] Schapira (2013), [13] Leenders et al. (1986)
KE 5: Neuroinflammation	Neuroinflammation describes the activation of microglia and astrocytes, manifested by a shape change, induction of pro-inflammatory enzymes and cytokines, and a migration towards the site of damage. In response to pathogens or to damaged neurons, microglia are initially activated and subsequently they promote the reaction of astrocytes. Reactive glial cells represent rich sources of nitric oxide (^•^NO), superoxide (^•^O_2_^−^), and cytokines, thus possibly contributing to the damage of adjacent neurons. Chronic neurodegenerative diseases such as Parkinson’s disease are characterized by a persistent inflammatory activation of glial cells [1–10]	Detection of microglia per volume of brain mass (CD11b, Iba1, Isolectin B4 staining) [11, 12] Live detection by PET imaging of microglial markers, e.g., by [^11^C]-PK 11195 [13] Detection of shape change of microglia and astrocytes [14] Detection of the astrocyte marker GFAP, that is upregulated upon inflammatory activation [15, 16] Detection of pro-inflammatory markers in glial cells by PCR, western blot, staining (in vitro, in vivo) [17]	Glial activation is found in all neurodegenerative conditions, but the exact activation state is often undefined and may be heterogenous. For microglia, at least two major states (M1 and M2) can be distinguished. Neurotoxic astrocytes can, e.g., be induced by activated microglia [18], whereas an alternative activation of astrocytes by microglia via P2Y1 receptor downregulation leads to neuroprotective conditions [19]. Therefore, a more exact characterization of this KE is required [1] Aschner (1998), [2] Graeber and Streit (1990), [3] Monnet-Tschudi et al. (2007), [4] Streit et al. (1999), [5] Kraft and Harry (2011), [6] Claycomb et al. (2013), [7] Brown and Bal-Price (2003), [8] Nakajima and Kohsaka (2004), [9] Falsig et al. (2008), [10] Falsig et al. (2006), [11] Monnet-Tschudi et al. (2011), [12] von Tobel et al. (2014), [13] Banati (2002), [14] Falsig et al. (2004), [15] Eng et al. (2000), [16] Struzynska et al. (2007), [17] Kuegler et al. (2010), [18] Liddelow et al. (2017), [19] Shinozaki et al. (2017)
Adverse outcome: Parkinsonian motor deficits	Motor information is modulated by the basal ganglia of the extrapyramidal system and returned to the motor cortex from where the processed information is projected to the periphery. The striatum represents the key modulatory site. Levels of DA in the striatum influence the degree of stimulatory output of the basal ganglia system that returns to the motor cortex and hence positively affects motor output to the periphery. A reduction of striatal DA, as consequence of nigrostriatal DA neurodegeneration, results in an inhibition of the terminal output nucleus and hence to a reduced feedback loop signal back to the motor cortex. By its involvement in a complex series of interactions between various basal ganglia, a reduction of striatal DA leads to an impaired motor output [1–11]	Behavioral tests *Rotation*: unilateral lesion of nigrostriatal DA neurons by experimental toxicants; asymmetric motor behavior (rotations upon stimulation with amphetamine) [12] *Rotarod*: assessment of motor coordination. Animals are placed on a rotating rod. Detection of the latency to fall [13] *Hang test*: a grid is inverted with the animal hanging upside down: detection of the time the animal hangs on the grid [14] *Forepaw stride length*: the distance between single steps is measured [15] *Grid test*: mice hang upside down on a grid, the percentage of unsuccessful forepaw steps is detected [16] *Akinesia*: the animal is placed on a flat surface, the latency until movement of all four limbs is assessed *Open field test*: detection of locomotion, distance travelled, number of rearings *Pole test*: animal on a pole, head upwards. Detection of the time required for 180° turn and the time the animal requires to reach the floor	Note that the AOP is not Parkinson’s disease. It is rather a defined set of particular motor symptoms. These are found in PD together with other features and symptoms, but they are also found after exposure to various toxicants damaging the nigrostriatal system [17] [1] Barnes (1983), [2] Bernheimer et al. (1973), [3] Silverdale et al. (2003), [4] Smith et al. (1994), [5] Bolam et al. (2000), [6] Gerfen et al. (1990), [7] Mitchell et al. (1989), [8] Smith and Kieval (2000), [9] Yuan et al. (2010), [10] Heimer et al. (2006), [11] Odekerken et al. (2013), [12] Ungerstedt and Arbuthnott (1970), [13] Jones and Roberts (1968), [14] Tillerson and Miller (2002), [15] Klapdor et al. (1997), [16] Crawley (1999), [17] Tieu (2011)

The table provides a condensed overview on the underlying mechanisms of the key events, including the most widely applied analytical detection methods

**Table 2:** Essentiality of key events

Key event	Interventions	Weight of evidence	Essentiality
KE 1: Inhibition of complex I	Expression of the inhibitor-insensitive oxidoreductase NDI-1, or circumvention of complex I by alternative electron shuttles protect from complex I inhibitor-dependent mitochondrial dysfunction, impaired proteostasis, and degeneration of DA neurons [1–6]	A strong experimental basis indicates the activation of KE_downstream_ upon KE 1 activation as well as their absent activation upon inhibition of KE 1 activation	The available experimental basis allows rating of KE 1 essentiality as: STRONG
KE 2: Mitochondrial dysfunction	Antioxidants, or maintenance of cellular ATP by creatine/phosphocreatine, protects from impaired proteostasis and from neurodegeneration [7–19]	Mitochondrial dysfunction summarizes a set of complex processes (e.g., decline in respiration, ROS formation, etc.). Experimental interference with the most prominent features of mitochondrial dysfunction clearly shows absence of KE_downstream_ activation	The available experimental basis allows rating of KE 2 essentiality as: STRONG
KE 3: Impaired proteostasis	Stimulation of autophagy protects from DA neurodegeneration [20, 21]	Following complex I inhibition, no unifying picture on the role of autophagy (activation, inhibition) emerged so far. Only moderate experimental evidence for a causal relationship between KE 3 and KEs_downstream_ in the absence of KE 1 and KE 2 is currently available	The available experimental basis allows rating of KE 3 essentiality as: MODERATE
KE 4: DA Neurodegeneration	Supplementation with L-DOPA, or replacement of degenerated DA neurons by transplants reverses parkinsonian motor deficits [22–39]	A strong experimental basis is available for the association between nigrostriatal DA neurodegeneration and the onset of the AO in rodents, monkeys and humans exposed to complex I inhibitors. Strong evidence indicates the reversibility of AO effects by DA neuron replacement	The available experimental basis allows rating of KE 4 essentiality as: STRONG
KE 5: Neuroinflammation	Intervention with pro-inflammatory signaling cascades (e.g., IL-1β, IFN-γ, TNF-α) protects from neurodegeneration and from the onset of parkinsonian motor deficits [40–52]	Neuroinflammation is regularly observed in association with complex I inhibitor action in vivo. However, quantitative information on the extent and type of neuroinflammation are missing. Neuroinflammation acts as self-amplifying feed-forward mechanism that impedes its linear integration into the structure of the present AOP	The available experimental basis allows rating of KE 5 essentiality as: MODERATE

**Fig. 2:**
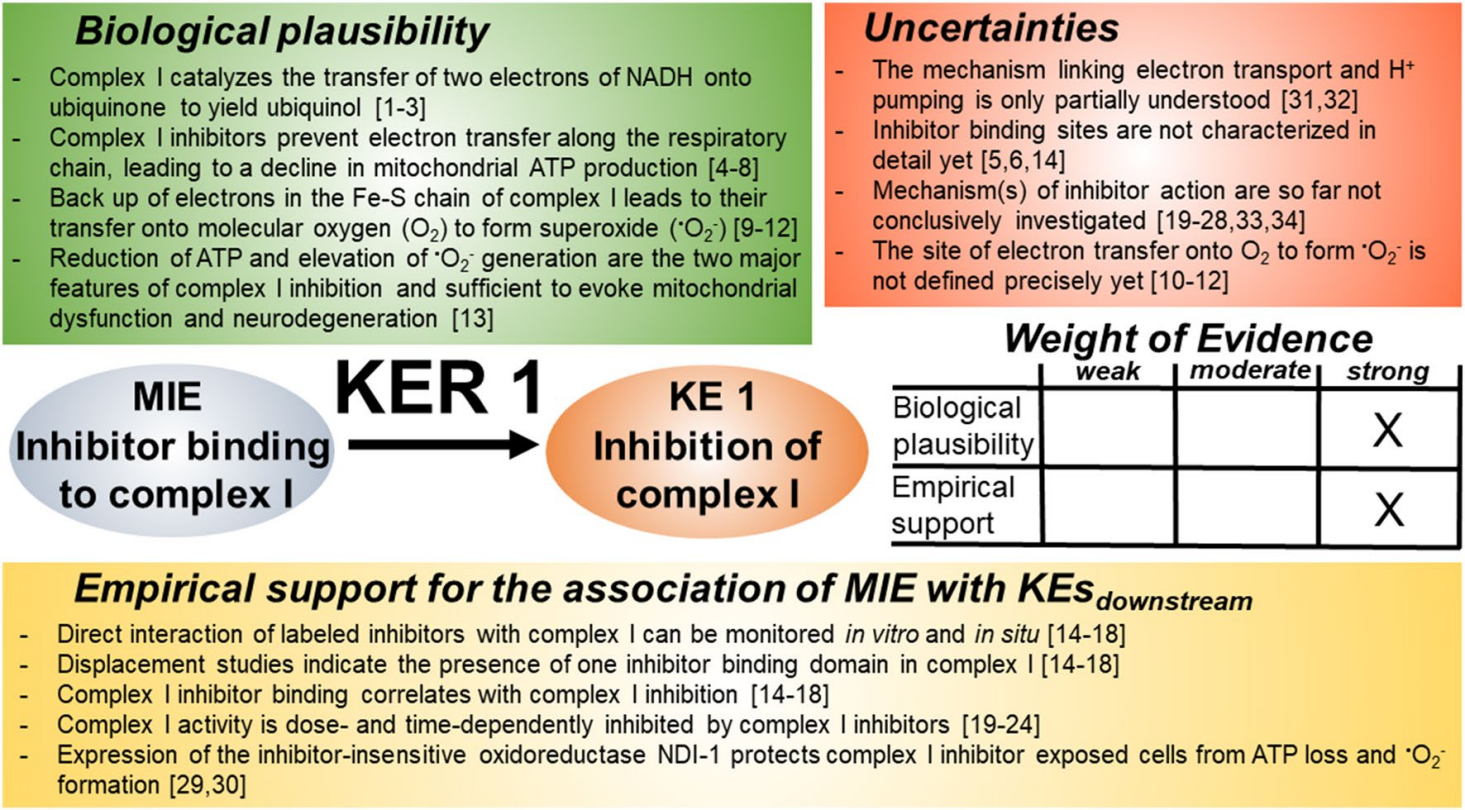
Key event relationship 1 (KER 1), linking inhibitor binding to complex I (MIE) and the inhibition of complex I (KE 1). The table shows the result of a qualitative assessment of KER 1 on a 3 point scale (weak, moderate, strong). Biological plausibility and experimental support were rated “strong”, according to the available body of experimental support in the literature. However, the molecular mechanisms associated with electron transfer along the respiratory chain, as well as the sites of inhibitor binding and the mechanisms underlying inhibitor-dependent inactivation of complex I, are not fully elucidated yet. *NADH* nicotinamide adenine dinucleotide, *ATP* adenosine triphosphate, *NDI-1* yeast NADH dehydrogenase. References: [1] Suzuki and King (1983), [2] Kotlyar et al. (1990), [3] van Belzen et al. (1997), [4] Palmer et al. (1968), [5] Degli Esposti et al. (1996), [6] Friedrich et al. (1994), [7] Ohnishi (1998), [8] Lümmen (1998), [9] Brand (2010), [10] Genova et al. (2001), [11] Galkin and Brandt (2005), [12] Lambert and Brand (2004), [13] Schildknecht et al. (2009), [14] Okun et al. (1999), [15] Talpade et al. (2000), [16] Ino et al. (2003), [17] Greenamyre et al. (1992), [18] Higgins and Greenamyre (1996), [19] Grivennikova et al. (1997), [20] Greenamyre et al. (2001), [21] Lambert and Brand (2004), [22] Ichimaru et al. (2008), [23] Okun et al. (1999), [24] Cleeter et al. (1992), [25] Friedrich et al. (1994), [26] Degli Esposti et al. (1993); [27] Degli Esposti and Ghelli (1994), Degli Esposti et al. (1994), [28] Höllerhage et al. (2009), [29] Seo et al. (1998), [30] Sherer et al. (2003), [31] Sharma et al. (2009), [32] Hirst (2013), [33] Vinogradov et al. (1995), [34] Albracht et al. (1997)

Assessment of the essentiality of the KEs is based on the availability of reliable assays for their quantitative detection (Fig. 2) and on their relevance in the progression of the biological perturbation, ultimately leading to the AO. The table lists the most robust intervention strategies at the respective KE that result in the absence of KE_downstream_ activation

References: [1] Seo et al. (1998), [2] Sherer et al. (2003), [3] Sharma et al. (2009), [4] Hirst (2013); [5] Vinogradov et al. (1995), [6] Albracht et al. (1997), [7] Beal (2011), [8] Przedborski et al. (1992), [9] Zhang et al. (2000), [10] Filomeni et al. (2012), [11] Wang et al. (2015), [12] Nataraj et al. (2016), [13] Lee et al. (2011), [14] Tseng et al. (2014), [15] Liu et al. (2015), [16] Thomas et al. (2012), [17] Pöltl et al. (2012), [18] Bose and Beal (2016), [19] Brownell et al. (1998), [20] Pan et al. (2009), [21] Seo et al. (2002), [22] Lloyd et al. (1975), [23] Yam et al. (1998), [24] Gilmour et al. (2011), [25] Heimer et al. (2002), [26] Papa et al. (1999), [27] Hutchinson et al. (1997), [28] Levy et al. (2001); [29] Parkinson Study Group (1993), [30] Pålhagen et al. (1998); [31] Pålhagen et al. (2006), [32] Parkinson Study Group (1996), [33] Olanow et al. (2008), [34] Widner et al. (1992), [35] Kordower et al. (1998), [36] Kordower et al. (1995), [37] Mendez et al. (2008), [38] Schumacher et al. (2000), [39] Ben-Hur et al. (2004), [40] Tanaka et al. (2013), [41] Mount et al. (2007), [42] Ferger et al. (2004), [43] Leng et al. (2005), [44] Sriram et al. (2002), [45] Sriram et al. (2006), [46] Qin et al. (2007), [47] McCoy et al. (2006), [48] Castaño et al. (2002), [49] Brochard et al. (2009), [50] Reynolds et al. (2007), [51] Laurie et al. (2007), [52] Liu et al. (2016)

Experimental studies illustrating a direct correlation between two adjacent KEs were also listed in the “experimental support” section of the respective KERs and in the respective figures. This organization of information is not fully in line with the recommendations of the AOP handbook. However, we opted for this solution for two reasons: (1) it allows a concise overview of the vast body of information included in the full version of the present AOP (AOP 3, AOP Wiki), as required for a journal article; (2) for many academic researchers, it is easier to understand the importance of a KER, if not only correlative data around the KER are present, but if this is combined with experimental evidence that modulation of the KE directly upstream of the KER leads to a modulation of the KE directly downstream of the respective KER.

## Key event relationships (KERs)

### KER 1: relationship between “binding of an inhibitor to mitochondrial complex I” (MIE) and “complex I inhibition” (KE 1) (Fig. 2)

#### Biological plausibility

Oxidation of nicotinamide adenine dinucleotide (NADH) is catalyzed by the flavine mononucleotide moiety of complex I (Vinogradov 1993; Degli and Ghelli 1994). In a sequential manner, the two electrons of NADH are transferred along a chain of eight Fe–S clusters to the ubiquinone-binding site where they reduce ubiquinone (Q), via ubisemiquinone (^•^Q) formation, to ubiquinol (QH_2_) (Kotlyar et al. 1990; Suzuki and King 1983; van Belzen et al. 1997). The majority of complex I inhibitors block the electron transfer onto ubiquinone (Palmer et al. 1968). Complex I inhibitors were categorized into three classes based on their potential binding site (Degli Esposti 1998; Friedrich et al. 1994). However, more recent research indicates the presence of a single inhibitor-binding pocket in the hydrophobic ubiquinone-binding region of complex I with several binding sites for structurally diverse inhibitors (Okun et al. 1999). The majority of currently described complex I inhibitors either prevent access of ubiquinone to its binding site, or the inhibitors act as electron acceptors interfering with the Fe–S cluster electron transport chain (Lümmen 1998; Ohnishi 1998). In all of these inhibitor-mediated cases, blockade causes electrons to back up, resulting in the full reduction of upstream Fe–S clusters (Brand 2010). These conditions promote an uncoordinated flux of electrons from reduced sites of complex I onto molecular oxygen to form the superoxide radical anion (^•^O_2_^−^) (Grivennikova and Vinogradov 2006; Liu et al. 2002), and they all prevent reduction of ubiquinone and thus the transfer of electrons through complexes III and IV to molecular oxygen. The N_2_ cluster, as well as flavine in its fully reduced or semiquinone form, have been suggested as molecular sites of superoxide formation upon complex I inhibition. These observations, however, are all dependent on the experimental system and procedures applied. Hence, they allow no generally accepted conclusion on the precise molecular site responsible for superoxide formation upon complex I inhibition (Brand 2010; Galkin and Brandt 2005; Genova et al. 2001; Lambert and Brand 2004).

#### Empirical support

The experimental basis for a causal relationship between inhibitor binding and complex I inhibition is based on experiments performed with submitochondrial particles, isolated mitochondria, and neuronal cell cultures. Real-time displacement tests using fluorescent (e.g., aminoquinazoline) or radioactively labeled complex I inhibitors and their derivatives (e.g., ^3^H-dihydrorotenone, ^3^H-AE F119209) provide direct evidence for the binding of complex I inhibitors (Greenamyre et al. 1992; Higgins and Greenamyre 1996; Ino et al. 2003; Okun et al. 1999; Talpade et al. 2000). Complex I activity is assessed by detection of NADH oxidation (Gluck et al. 1994; Höllerhage et al. 2009; Shimomura et al. 1989). Time- and concentration-dependent inhibition of complex I in submitochondrial particles or isolated mitochondria was observed with rotenoids, piercidines, myxobacterial antibiotics, and vanilloids such as capsaicin (Cleeter et al. 1992; Degli Esposti et al. 1993, 1994; Friedrich et al. 1994; Greenamyre et al. 2001; Grivennikova et al. 1997; Höllerhage et al. 2009; Ichimaru et al. 2008; Lambert and Brand 2004; Miyoshi 1998; Okun et al. 1999). The inhibitory action of complex I inhibitors on electron transfer onto ubiquinone was independently confirmed by the expression of the inhibitor-insensitive oxidoreductase NDI-1 from *Saccharomyces cerevisiae* in cell models, which circumvents complex I and allows maintenance of the normal respiratory chain electron flux (Seo et al. 1998; Sherer et al. 2003).

#### Uncertainties

The question of how electron transfer is coupled to proton pumping has still not been answered in a conclusive manner (Hirst 2013; Sharma et al. 2009). Electron paramagnetic resonance (EPR) analyses indicated the presence of two ubisemiquinone species during electron transport (Vinogradov et al. 1995). However, it is not known whether these species represent two independent ubisemiquinone molecules or two forms of the same semiquinone (Albracht et al. 1997). Inhibitor binding studies are performed with submitochondrial particles, containing membranes. Due to the lipophilicity of most complex I inhibitors, these investigations suffer from high background values as a result of unselective membrane binding (Horgan and Casida 1968). Although complex I inhibitors prevent ubiquinol formation, the precise inhibitor binding site(s) have not been identified yet. Furthermore, it is not evident whether ^•^O_2_^−^ generation upon complex I inhibition is mainly derived from F–S cluster or from semiquinone-dependent electron transfer onto molecular oxygen.

### KER 2: relationship between “complex I inhibition” (KE 1) and “mitochondrial dysfunction” (KE 2) (Fig. 3)

**Fig. 3:**
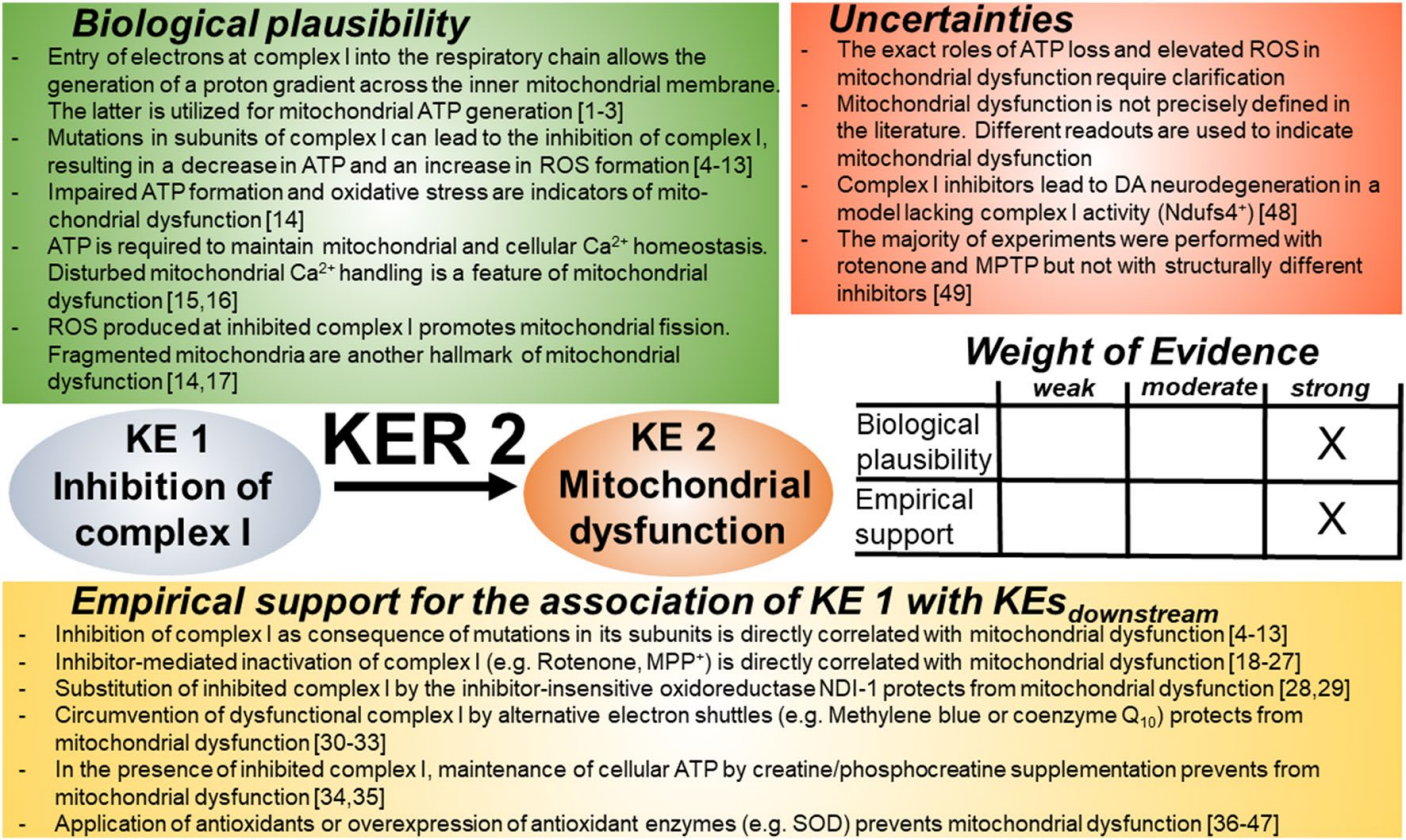
Key event relationship 2 (KER 2), linking the inhibition of complex I (KE 1) and mitochondrial dysfunction (KE 2). The table shows the result of a qualitative assessment of KER 2 on a 3 point scale (weak, moderate, strong). Biological plausibility and empirical evidence were rated “strong”, based on the vast body of experimental evidence available in the literature. A threshold of complex I inhibition, necessary for the induction of mitochondrial dysfunction, has so far not been defined in the literature. Similar limitations apply for the quantitative assessment, respectively, the definition, of mitochondrial dysfunction. *ATP* adenosine triphosphate, *DA* dopamine, *PD* Parkinson’s disease, *NDUFS* subunits of NADH-ubiquinone oxidoreductase (complex I), *ROS* reactive oxygen species, *NDI-1* yeast NADH dehydrogenase. References: [1] Wirth et al. (2016), [2] Friedrich et al. (1994), [3] Mailloux (2015), [4] Fernandez-Moreira et al. (2007), [5] Berger et al. (2008), [6] Hoefs et al. (2008), [7] Janssen et al. (2006), [8] Lazarou et al. (2009), [9] Dunning et al. (2007), [10] Ogilvie et al. (2005), [11] Saada et al. (2008), [12] Pagliarini et al. (2008), [13] Koopman et al. (2007), [14] Sheehan et al. (1997), [15] Willems et al. (2008), [16] Ye et al. (2015), [17] Han et al. (2016), [18] Dukes et al. (2016), [19] Wang et al. (2011), [20] Li et al. (2014), [21] Giordano et al. (2012), [22] Piao et al. (2012), [23] Wu et al. (2009), [24] Bi et al. (2008), [25] Nakai et al. (2003), [26] Brownell et al. (1998), [27] Koga et al. (2006), [28] Seo et al. (1998), [29] Sherer et al. (2003), [30] Shults et al. (2002), [31] Moon et al. (2005), [32] Wen et al. (2011), [33] Yang et al. (2009), [34] Matthews et al. (1999), [35] Beal (2011); [36] Przedborski et al. (1992), [37] Zhang et al. (2000), [38] Filomeni et al. (2012), [39] Wang et al. (2015), [40] Nataraj et al. (2016), [41] Lee et al. (2011), [42] Tseng et al. (2014), [43] Liu et al. (2015), [44] Thomas et al. (2012), [45] Pöltl et al. (2012), [46] Bose and Beal (2016), [47] Brownell et al. (1998), [48] Choi et al. (2008), [49] Höllerhage et al. (2009)

#### Biological plausibility

Complex I represents the principal gateway for the entry of electrons into the mitochondrial respiratory chain (Friedrich et al. 1994; Wirth et al. 2016). A functional respiratory chain generates a proton gradient across the inner mitochondrial membrane, exploited in a subsequent step by mitochondrial ATPases to generate ATP (Brandt 1997; Mailloux 2015). Disturbances in the electron transfer through complex I lead to an impaired proton gradient and reduced ATP generation. As a consequence of limited ATP availability, mitochondrial Ca^2+^ homeostasis is disturbed, thus contributing to mitochondrial dysfunction (high energy demand of Ca^2+^ ATPases) (Sheehan et al. 1997; Willems et al. 2008). In parallel to the reduction in ATP generation, blockade of the electron flow along the respiratory chain results in an accidental reduction of molecular oxygen to form superoxide (^•^O_2_^−^) (Mailloux 2015). Elevated reactive oxygen species (ROS) levels promote oxidative damage of mitochondrial DNA, proteins, and lipids, and trigger mitochondrial fragmentation (Koopman et al. 2007; Willems et al. 2009). The loss of the mitochondrial transmembrane potential, impaired mitochondrial ATP generation, disturbances in mitochondrial Ca^2+^ homeostasis, as well as the production of harmful ROS levels are features collectively referred to as mitochondrial dysfunction (Bose and Beal 2016). Deficiencies in complex I activity are regularly observed in association with mutations in mtDNA or nuclear DNA-encoded complex I genes. Mutations in nuclear-encoded complex I genes have been demonstrated for 12 structural subunits of complex I (Berger et al. 2008; Fernandez-Moreira et al. 2007; Hoefs et al. 2008) and for five complex I assembly factors (Dunning et al. 2007; Janssen et al. 2006; Lazarou et al. 2009; Ogilvie et al. 2005; Pagliarini et al. 2008; Saada et al. 2008). Fibroblasts of patients with such complex I mutations exhibit a decreased mitochondrial transmembrane potential and mitochondrial ATP generation, as well as elevated ^•^O_2_^−^ formation by complex I and hence meet the definition of mitochondrial dysfunction (Koopman et al. 2007).

#### Empirical support

Experimental support for a causal relationship between complex I inhibition and mitochondrial dysfunction is largely based on observations made with the complex I inhibitors rotenone and 1-methyl-4-phenyl-1,2,3,6-tetrahydropyridine (MPTP). A rich experimental basis indicates the direct correlation between complex I inhibition and the emergence of features of mitochondrial dysfunction in cellular and in vivo models exposed to rotenone or MPTP/MPP^+^ (Bi et al. 2008; Dukes et al. 2016; Giordano et al. 2012; Han et al. 2016; Li et al. 2014; Nakai et al. 2003; Piao et al. 2012; Schildknecht et al. 2009; Scholz et al. 2011; Wang et al. 2011; Wu et al. 2009; Ye et al. 2015). Initial studies using proton magnetic resonance spectroscopy (^1^H-MRS) and positron emission tomography (PET) have illustrated the onset of mitochondrial dysfunction by live measurements in living animals exposed to MPTP (Brownell et al. 1998; Koga et al. 2006). Experimental interventions to prevent impaired mitochondrial ATP generation, e.g., by expression of the inhibitor-insensitive oxidoreductase NDI-1 from *S. cerevisiae* to circumvent impaired endogenous complex I, protect from mitochondrial dysfunction (Seo et al. 1998; Sherer et al. 2003). Application of alternative electron shuttles, such as methylene blue or coenzyme Q_10_, (Moon et al. 2005; Shults et al. 2002; Wen et al. 2011) or boosting of cellular ATP levels by supplementation of cells exposed to complex I inhibitors with creatine/phosphocreatine (Beal 2011; Matthews et al. 1999; Yang et al. 2009) also protect from mitochondrial dysfunction and neuronal demise.

The second strategy to protect from impaired complex I-dependent mitochondrial dysfunction targets complex I-mediated ^•^O_2_^−^ formation. Overexpression of superoxide dismutase (SOD) protects from the toxic influence of MPTP (Przedborski et al. 1992) whereas knockdown of endogenous SOD elevates the sensitivity of mice towards MPTP-dependent mitochondrial dysfunction and nigrostriatal cell loss (Zhang et al. 2000). In cellular and in vivo models exposed to rotenone or MPTP, antioxidants protect from complex I inhibition-dependent mitochondrial dysfunction (Filomeni et al. 2012; Lee et al. 2011; Liu et al. 2015; Nataraj et al. 2016; Thomas et al. 2012; Tseng et al. 2014; Sherer et al. 2003; Wang et al. 2015).

A more detailed analysis indicates a mutual interaction between ROS and complex I. While complex I acts as a potent source of ^•^O_2_^−^ following its inhibition, an experimental decline of cellular glutathione levels (e.g., γ-glutamylcysteine synthetase knockdown, treatment with buthionine sulfoximine) (Jha et al. 2000; Chinta and Andersen 2006) correlates with a reduction of complex I activity, the onset of mitochondrial dysfunction, and ultimately with the demise of dopaminergic (DA) neurons. All of these KEs were prevented by the application of thiol antioxidants such as dithiothreitol (DTT) or *N*-acetylcysteine (NAC) (Chinta and Andersen 2006; Jha et al. 2000). Mechanistic investigations unraveled reversible S-nitrosation of complex I, leading to its inhibition (Dahm et al. 2006; Burwell et al. 2006). Thiol antioxidants evoke a de-nitrosation and are associated with a re-activation of complex I, preventing mitochondrial dysfunction and protecting from neurodegeneration (Dahm et al. 2006; Borutaite et al. 2000).

These observations indicate that targeting either impaired ATP generation or elevated ^•^O_2_^−^ formation as the two direct consequences of complex I inhibition, represents an effective intervention strategy, capable of preventing the activation of KEs downstream of complex I inhibition (KE 1).

#### Uncertainties

Complex I inhibition results in a reduction in mitochondrial ATP generation and an elevation of ^•^O_2_^−^ formation. To date, the respective contribution of these two factors to mitochondrial dysfunction has not been quantified. A cell model devoid of classical complex I activity (Choi 2008) still shows effects of rotenone or MPP^+^. This may be due to a contribution by off-target effects, e.g., on microtubules (Brinkley et al. 1974; Marshall and Himes 1978), but the data have not been confirmed by others. The vast majority of experimental evidence on the relationship between complex I inhibition and the onset of parkinsonian motor deficits is based on the use of the complex I inhibitors rotenone and MPTP/MPP^+^. A relatively wide spectrum of structurally different complex I inhibitors have been described over the course of recent decades. Prominent examples are acetogenins (Bermejo et al. 2005), tetrahydroisoquinolines (Morikawa et al. 1996), antibiotics such as piericidin A (Degli Esposti 1998; Friedrich et al. 1994; Kubota et al. 2003; Horgan et al. 1968; Singer 1979), insecticides such as quinazolines or acetogenins (Ahammadsahib et al. 1993; Hollingworth et al. 1994), quinones (Kean et al. 1971), and vanilloids (Shimomura et al. 1989). All of these structurally different complex I inhibitors have been characterized with isolated mitochondria or with submitochondrial particles. Robust *K*_i_ values and functional studies involving neuronal cell cultures or in vivo models are rather rare. A systematic comparison of the half maximal inhibitory concentration (IC_50_) values for complex I inhibition and half maximal binding concentration (EC_50_) values for the reduction of ATP levels was performed with rat fetal striatal neurons (Höllerhage et al. 2009). Due to the lipophilicity of most of the complex I inhibitors tested, the detected EC_50_ values were in most cases lower than the IC_50_ values detected for complex I inhibition.

### KER 3: relationship between “mitochondrial dysfunction” (KE 2) and “impaired proteostasis” (KE 3) (Fig. 4)

**Fig. 4:**
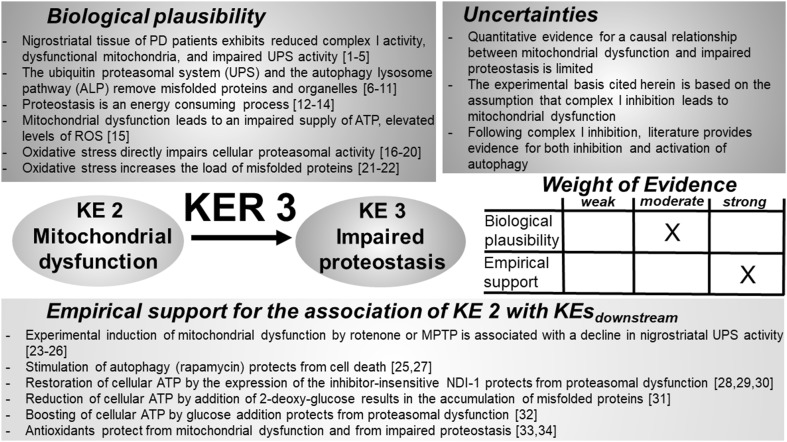
Key event relationship 3 (KER 3), linking mitochondrial dysfunction (KE 2) and impaired proteostasis (KE 3). The table shows the result of a qualitative assessment of KER 3 on a 3 point scale (weak, moderate, strong). While a strong experimental basis exists in the literature to justify the rating “strong” for the experimental support linking KE 2 and KE 3, mechanistic understanding on how mitochondrial dysfunction, respectively, its individual features such as a decline in ATP generation, or an elevated formation of free radical species, affect cellular proteostasis, are only incompletely understood. The situation is further complicated by mutual interactions between mitochondrial dysfunction, oxidative stress, and proteasomal stress that lead to self-amplifying futile cycles but allow no definition on an initiating event. *PD* Parkinson’s disease, *UPS* ubiquitin proteasomal system, *ALP* autophagy–lysosomal pathway, *ATP* adenosine triphosphate, *ROS* reactive oxygen species, *MPTP* 1-methyl-4-phenyl-1,2,3,6-tetrahydropyridine, *NDI-1* single subunit NADH dehydrogenase of *S. cerevisiae*. References: [1] Betarbet et al. (2005), [2] McNaught et al. (2003), [3] McNaught and Jenner (2001a, b), [4] Ambrosi et al. (2014), [5] Yu et al. (2009), [6] Martini-Stoica et al. (2016), [7] Komatsu et al. (2006), [8] Menzies et al. (2015), [9] Goldberg (2003), [10] Ding et al. (2003), [11] Zheng et al. (2016), [12] Pickart and Cohen (2004), [13] Finley (2009), [14] Voges et al. (1999), [15] Bose and Beal (2016), [16] Wang et al. (2010, b), [17] Farout et al. (2006), [18] Ishii et al. (2005), [19] Demasi et al. (2003), [20] Demasi et al. (2001), [21] Butterfield and Kanski (2001), [22] Sayre et al. (2001), [23] Fornai et al. (2005), [24] Wu et al. (2015), [25] Liu et al. (2013), [26] Yong-Kee et al. (2012), [27] Pan et al. (2009), [28] Seo et al. (2002), [29] Seo et al. (2000), [30] Seo et al. (1998), [31] Sherer et al. (2003), [32] Shamoto-Nagai et al. (2003), [33] Chou et al. (2010), [34] Filomeni et al. (2012)

#### Biological plausibility

The two main systems for the removal of misfolded proteins are: (1) the autophagy–lysosomal pathway (ALP), which removes dysfunctional proteins, aggregates, and even subcellular organelles; and (2) the ubiquitin proteasomal system (UPS), which catalyzes the tagging of target proteins by ubiquitination, followed by their degradation via the 26S proteasome (Ding et al. 2003; Goldberg 2003; Komatsu et al. 2006; Martini-Stoica et al. 2016; Menzies et al. 2015; Zheng et al. 2016). The correlation between mitochondrial dysfunction and impaired proteostasis is based on the considerations that (1) proteostasis is an energy-consuming process requiring ATP from mitochondria, and that (2) components of the proteasomal system are subject to inhibition by ROS, generated from dysfunctional mitochondria (Finley 2009; Pickart and Cohen 2004). The 26S proteasome catalyzes ATP-dependent protein degradation and consists of a 20S core, associated with a regulatory 19S particle (Kim et al. 2011; Murata et al. 2009; Voges et al. 1999). Oxidative stress causes the dissociation of the 20S core from the regulatory 19S particle, leading to the loss of 26S proteasome activity (Wang et al. 2010). Under conditions of impaired ATP synthesis and elevated ROS levels, the interaction of the 20S core with alternative activation proteins yields a 20S proteasome without de-ubiquitination and ATPase activities (Schmidt et al. 2005; Ma et al. 1992). The ATP-independent 20S proteasome is also subject to posttranslational modifications such as hydroxynonenal modifications, carbonylation, or S-glutathionylation (Demasi et al. 2001, 2003; Farout et al. 2006; Ishii et al. 2005), but displays a higher resistance to oxidative stress in comparison with the 26S proteasome (Reinheckel et al. 1998, 2000). In parallel to the direct inhibition of cellular protein degeneration systems, oxidative stress increases the load of modified and misfolded proteins as substrates of the degradation machinery (Butterfield and Kanski 2001; Sayre et al. 2001), similar to what occurs in mitochondrial dysfunction. Analysis of ALP activity upon inhibition of complex I provides a heterogeneous picture, with several reports illustrating an impairment of ALP activity (Lim et al. 2011; Mader et al. 2012; Pan et al. 2009; Sarkar et al. 2014), while others describe an activation (Chen et al. 2007; Chu et al. 2013; Zhu et al. 2007a). Autophagy has been suggested as a component of the cellular antioxidant system, based on its removal of oxidatively modified proteins (Giordano et al. 2013). It is hence speculated that activation of ALP represents a countermeasure of the cell in early stages of mitochondrial dysfunction, while later stages are characterized by a decline in autophagy activity and an associated decline in cell viability. The PD-associated protein alpha synuclein (ASYN) emerged as a key element connecting mitochondrial dysfunction and impaired proteostasis. Knockdown of ASYN protects from complex I inhibition-mediated neurodegeneration (Zharikov et al. 2015), while ASYN overexpression sensitizes neurons towards secondary stressors (Chartier-Harlin et al. 2004; Singleton et al. 2003). Mitochondrial dysfunction leads to an accumulation and an aggregation of oxidative modified ASYN (Betarbet et al. 2006; Cannon et al. 2009). Vice versa, elevated ASYN levels evoke mitochondrial dysfunction (Hsu et al. 2000). In conclusion, mitochondrial dysfunction is characterized by an impaired ATP generation and elevated levels of ROS. Oxidative stress not only increases the load of misfolded proteins, it also leads to an impairment in the cellular protein degradation machineries. These energy-consuming processes are further hampered by the limitations in ATP supply under these conditions, hence resulting in an inadequate removal of misfolded proteins.

#### Empirical support

Most empirical support comes from cellular models exposed to rotenone and MPTP/MPP^+^. Moreover, an impairment of the UPS activity in the nigrostriatal system parallels mitochondrial dysfunction in PD patients (Ambrosi et al. 2014; Betarbet et al. 2005; McNaught and Jenner 2001a, b, 2003; Yu et al. 2009). Experimental induction of mitochondrial dysfunction in mice and rats by rotenone or MPTP is associated with a decline in nigrostriatal UPS (Fornai et al. 2005; Liu et al. 2013; Wu et al. 2015). In vitro models revealed that complex I inhibition precedes the onset of proteasomal impairment and the accumulation of ubiquitinated proteins (Yong-Kee et al. 2012). Expression of the inhibitor-insensitive single subunit NADH dehydrogenase NDI-1 protects from rotenone-induced loss of proteasomal function, underlining the contribution of ATP for proteasomal degradation (Seo et al. 2000, 2002). The tight dependency of proteasomal function on metabolic activity was demonstrated by a glucose-dependent experimental boost of cellular ATP levels, resulting in elevated protein degradation (Höglinger et al. 2003a, b). To avoid an involvement of complex I inhibitor-mediated oxidative stress, ATP was alternatively lowered by supplementation of cell medium with 2-desoxy-glucose. This resulted in an accumulation of misfolded proteins (Sherer et al. 2003). Management of oxidative stress, as the second dominating feature of mitochondrial dysfunction, by application of antioxidants, protects from complex I inhibitor-evoked proteasomal impairment and from an accumulation of ubiquitinated proteins (Chou et al. 2010; Filomeni et al. 2012; Shamoto-Nagai et al. 2003). The ALP can be stimulated by pharmacological means, and ALP stimulation by rapamycin results in a protection of the cell from complex I-mediated neurotoxicity (Liu et al. 2013; Pan et al. 2009). Similar to the situation observed with the UPS, antioxidants protect from complex I inhibitor-dependent reduction in ALP activity (Filomeni et al. 2012).

In neurons, proteostasis is largely influenced by intracellular trafficking processes. Mitochondrial and vesicular trafficking is affected by dysregulated cytosolic Ca^2+^ levels (Chang et al. 2006; Saotome et al. 2008; Yi et al. 2004) that emerge as a consequence of complex I inhibitor-mediated mitochondrial dysfunction. ASYN expression levels are directly correlated with microtubule instability (Chen et al. 2007; Esposito et al. 2007; Lee et al. 2006). Accumulation of ASYN leads to elevated levels of hyperphosphorylated tau protein and consequently to microtubule depolymerization (Qureshi and Paudel 2011). As a result of inappropriate transport processes, misfolded proteins and organelles accumulate within the cell.

ASYN levels are elevated in response to complex I inhibition (Betarbet et al. 2006; Cannon et al. 2009; Fornai et al. 2005). Elevated cytosolic levels of Ca^2+^ as consequence of complex I inhibition promote aggregation of ASYN (Follett et al. 2013; Goodwin et al. 2013; Nath et al. 2011; Yuan et al. 2015). Mitochondrial dysfunction and dysfunction in cellular Ca^2+^ homeostasis leads to disturbances in neuronal DA handling, leading to DA-mediated oxidative stress. DA-modified ASYN not only prevents its own degradation by the chaperone-mediated autophagy (CMA) pathway, but it also prevents the degradation of other proteins (Martinez-Vicente et al. 2008). ASYN filament formation, promoted by ASYN overexpression, or the expression of ASYN mutants, directly impairs proteasomal activity (Stefanis et al. 2001). Knockdown of endogenous ASYN, treatment with antioxidants, and supplementation with ATP protect from mitochondrial dysfunction-mediated onset of proteasomal stress (Betarbet et al. 2006; Dauer et al. 2002; Drolet et al. 2004; Shamoto-Nagai et al. 2003).

#### Inconsistencies

Impaired proteostasis includes an imbalance or dysfunction of a very large number of diverse biochemical processes. These are again interlinked in complex ways. While this is not an inconsistency as such, it can lead to inconsistent results in the literature, when different processes, often measured at different times, are used as biomarkers of impaired proteostasis. Inhibition of mitochondrial complex I is mainly characterized by impaired ATP production and elevated ^•^O_2_^−^ formation. Although these processes result in an impairment of various proteostasis mechanisms, such as UPS activity, defined molecular events linking KE 2 and KE 3 need further investigation. The relationship between mitochondrial dysfunction and impaired proteostasis is furthermore characterized by several mutual interactions, ultimately leading to a self-amplifying vicious cycle. Misfolded ASYN, for example, accumulates as a consequence of impaired proteostasis, and this in turn negatively influences mitochondrial integrity and function via its binding to the inner mitochondrial membrane and to their import machinery (Devi et al. 2008; Robotta et al. 2014). DA-modified ASYN, on the other hand, not only blocks its own degradation by the CMA pathway but also prevents CMA-dependent degradation of other proteins (Martinez-Vicente et al. 2008). Literature provides evidence for both activation and inhibition of autophagy activity upon experimental complex I inhibition. However, time-dependent and quantitative information on autophagy activity under these conditions is not available yet. One of the cardinal features of PD is the formation of Lewy bodies in the brain. While proteinaceous ASYN aggregates are observed in rotenone-exposed rats, Lewy body-like structures are not observed in MPTP models (Dauer et al. 2002; Drolet et al. 2004).

### KER 4: relationship between “impaired proteostasis” (KE 3) and “degeneration of DA neurons” (KE 4) (Fig. 5)

**Fig. 5:**
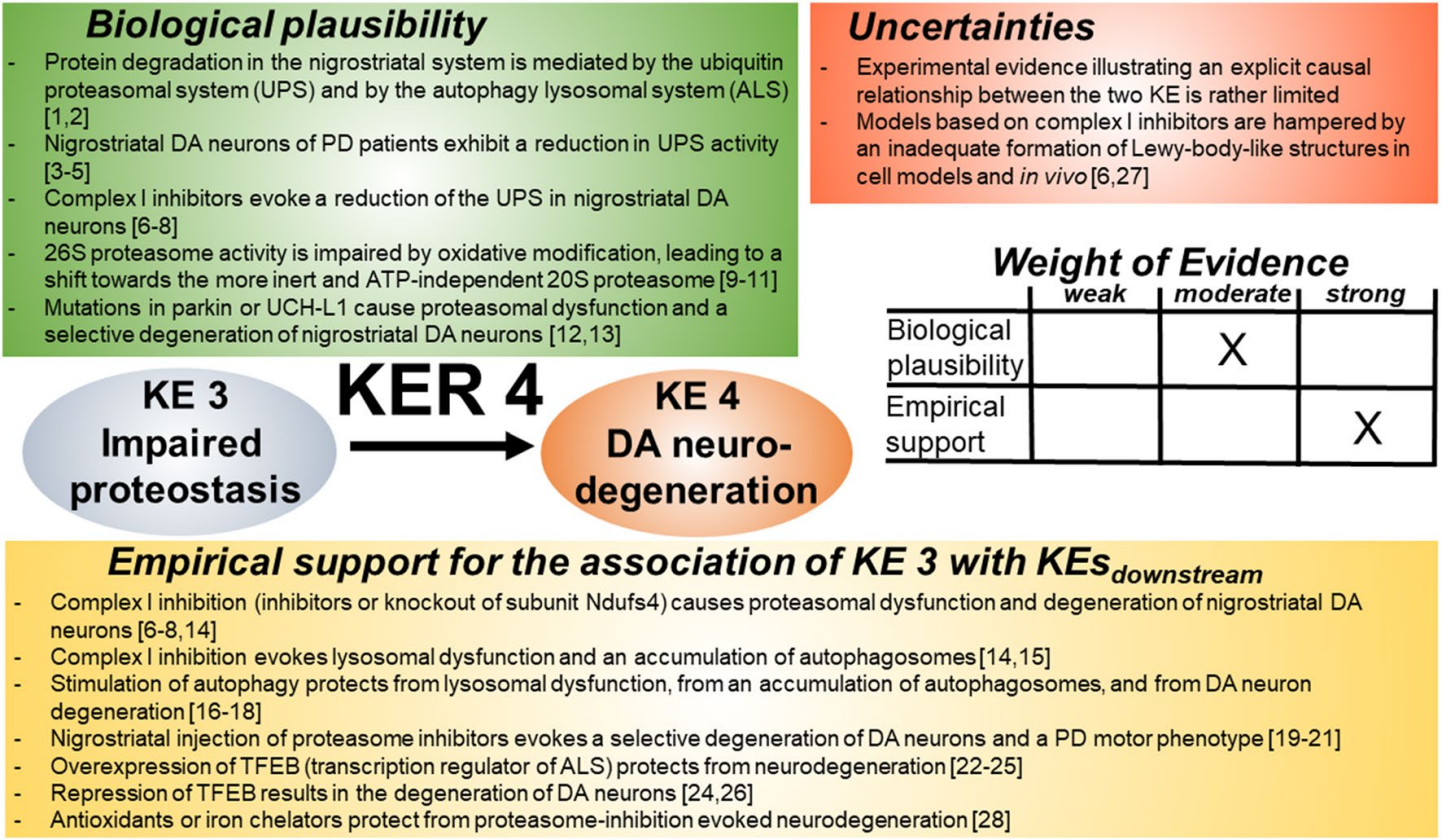
Key event relationship 4 (KER 4), linking impaired proteostasis (KE 3) and DA neurodegeneration (KE 4). The table shows the result of a qualitative assessment of KER 4 on a 3 point scale (weak, moderate, strong). Literature provides conclusive empirical support for a causal and quantitative relationship between KE 3 and KE 4. Insight into the molecular events responsible for DA neurodegeneration in response to impaired proteostasis, however, can only be classified “moderate” due to essential knowledge gaps. *UPS* ubiquitin proteasomal system, *ALS* autophagy–lysosomal system, *DA* dopamine, *UCH-L1* ubiquitin carboxy-terminal hydrolase L1, *Ndufs4* NADH:ubiquinone oxidoreductase subunit S4, *TFEB* transcription factor EB. References: [1] Martini-Stoica et al. (2016), [2] Menzies et al. (2015), [3] McNaught and Jenner (2001a, b), [4] McNaught et al. (2003), [5] Ambrosi et al. (2014), [6] Betarbet et al. (2000), [7] Betarbet et al. (2006), [8] Fornai et al. (2005), [9] Davies (2001), [10] Wang et al. (2010), [11] Schmidt et al. (2005), [12] Kitada et al. (1998), [13] Leroy et al. (1998), [14] Song and Cortopassi (2015), [15] Mader et al. (2012), [16] Dehay et al. (2010), [17] Wu et al. (2015), [18] Giordano et al. (2014), [19] Bentea et al. (2015), [20] Li et al. (2012), [21] Fornai et al. (2003), [22] Decressac et al. (2013), [23] Kilpatrick et al. (2015), [24] Decressac and Björklund (2013), [25] Ebrahimi-Fakhari and Wahlster (2013), [26] Decressac et al. (2012), [27] Shimoji et al. (2005), [28] Zhu et al. (2007b)

#### Biological plausibility

Impaired proteostasis leads to an accumulation of misfolded and modified proteins. These protein aggregates influence microtubule assembly and stability, resulting in a reduction in axonal transport of vesicles and mitochondria (Borland et al. 2008; Chen et al. 2007; O’Malley 2010) and a “dying back” degeneration pattern, starting in the periphery, ultimately leading to neurodegeneration (Braak et al. 2004; Grosch et al. 2016; Raff et al. 2002). ASYN is among the best-studied examples, linking proteostasis and neuronal degeneration. Accumulation of ASYN, either as a consequence of endogenous disturbances of proteostasis, experimental blockade of the proteasomal system, or by overexpression of its wild-type or mutant forms, leads to the disassembly of microtubules and, ultimately, to axonal damage (Esposito et al. 2007; Kirik et al. 2003; Masliah et al. 2000). Furthermore, ASYN protofibrils interact with intracellular organelles such as neurotransmitter vesicles or mitochondria, and lead to an uncontrolled release of DA and an impairment of mitochondrial function (Lotharius et al. 2002; Saha et al. 2004; Devi et al. 2008; Chinta et al. 2010). As mentioned above, DA-modified ASYN not only blocks its own degradation by the CMA pathway but also prevents the degradation of other proteins (Martinez-Vicente et al. 2008). Aggregates of wild-type or mutant forms of ASYN disturb controlled axonal transport of mitochondria (Li et al. 2013; Melo et al. 2017; Xie and Chung 2012). In neurons, key steps, such as mitochondrial fission/fusion or mitophagy, are conducted in the cell body. Impaired axonal transport of mitochondria hence leads to limited ATP supply and elevated levels of ROS, generated by dysfunctional mitochondria. The cellular 26S proteasome is a vulnerable target for free radical species originating from autoxidizing DA and mitochondria, leading to its inhibition and hence reinforcing proteasomal dysfunction (Davies 2001).

Analysis of nigrostriatal tissue of patients with PD has suggested an impairment in the activity of the 20/26S proteasome (McNaught and Jenner 2001a, b; McNaught et al. 2003). Similar observations were made in fibroblasts obtained from patients with PD, which exhibited elevated basal levels of ubiquitinated proteins and impaired 20S proteasomal activity (Ambrosi et al. 2014). The brain-region selective impairment of proteasomal activity correlates with the selective demise of DA neurons in this region (McNaught and Jenner 2001a, b; McNaught et al. 2003). Disturbances in the ubiquitin proteasomal system are also directly associated with prominent examples of mutations (e.g., parkin, ubiquitin C-terminal hydrolase L1) identified in genetic PD cases. Both are sufficient to cause preferential degeneration of nigrostriatal DA neurons (Leroy et al. 1998; Kitada et al. 1998).

#### Empirical support

Experimental evidence for a causal relationship between impaired proteostasis and DA neurodegeneration is based on in vitro and in vivo experiments involving complex I inhibitors and proteasome inhibitors. Several in vivo studies reported an impairment of the UPS, an accumulation of polyubiquitinated proteins, and the loss of nigrostriatal DA neurons upon exposure to complex I inhibitors (Betarbet et al. 2000, 2006; Fornai et al. 2005; Wang et al. 2006; Yong-Kee et al. 2012). An alternative complex I inactivation by conditional knockout of the complex I subunit Ndufs4 independently confirms the decrease in proteasomal activity and accumulation of polyubiquitinated proteins (Song and Cortopassi 2015). Exposure to complex I inhibitors leads to an accumulation of autophagosomes and a concomitant decrease in the number of lysosomes, as well as lysosomal dysfunction (Dehay et al. 2010; Mader et al. 2012). Up-regulation of autophagy, e.g., by rapamycin or trehalose, protects from lysosomal permeability and from neurodegeneration (Dehay et al. 2010; Giordano et al. 2014; Wu et al. 2015). A direct correlation between proteasomal dysfunction and neurodegeneration was observed by in vivo stereotaxic injection of proteasome inhibitors such as lactacystin or MG-132. Intracerebral proteasome inhibitor infusion evokes a preferential degeneration of nigrostriatal DA neurons, accompanied by the onset of PD-associated motor impairments (Bentea et al. 2015; Fornai et al. 2003; Li et al. 2012). Transcription factor EB (TFEB) is a key transcriptional regulator of the autophagy–lysosome pathway. Repression of TFEB expression in A9 and A10 DA neurons results in their accelerated degeneration (Decressac et al. 2013; Decressac and Björklund 2013). Overexpression of ASYN in vivo leads to lysosomal dysfunction and to cytoplasmic retention of TFEB. Overexpression of TFEB in the same model protects from DA neurodegeneration by clearance of ASYN oligomers (Decressac et al. 2013; Decressac and Björklund 2013; Ebrahimi-Fakhari and Wahlster 2013; Kilpatrick et al. 2015).

#### Inconsistencies

Mechanistic molecular information, as well as quantitative data on the direct causal relationship between impaired proteostasis and DA neurodegeneration is limited. Most of the information on the relationship of the two KEs is based on model systems treated with complex I inhibitors. However, MPTP/MPP^+^ does not recapitulate the formation of intracellular inclusions or aggregates. An increase in autophagy is reported as both protective and detrimental, most likely as a result of different degrees of activation or different observation times. Quantitative information on potential threshold activation levels for autophagy and their influence on cell integrity is currently not available.

### KER 5: relationship between “degeneration of DA neurons” (KE 4) and “neuroinflammation” (KE 5) (Fig. 6)

**Fig. 6:**
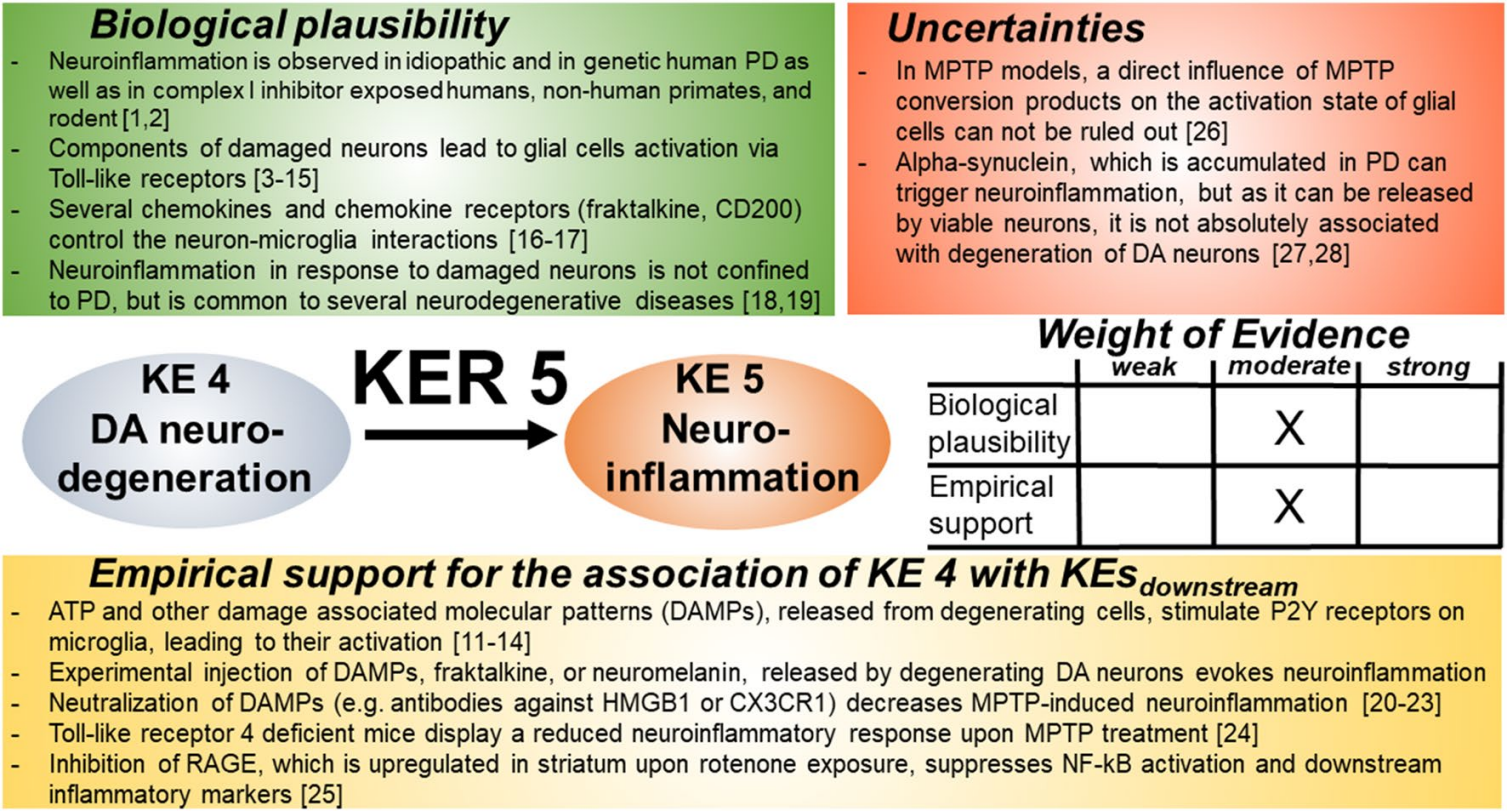
Key event relationship 5 (KER 5), linking DA neurodegeneration (KE 4) and neuroinflammation (KE 5). The table shows the result of a qualitative assessment of KER 5 on a 3 point scale (weak, moderate, strong). Both empirical support and biological plausibility were classified as “moderate”, based on the species-dependent variability of mediators originating from degenerating DA neurons. Experimental support for a causal link of KE 4 and KE 5 is mainly based on in vitro models, whereas in vivo information is rather limited. *DAMP* damage associated molecular patterns, *HMGB1* high mobility group box 1, *CX3CR1* fractalkine receptor, *MPTP* 1-methyl-4-phenyl-1,2,3,6-tetrahydropyridine, *RAGE* receptor for advanced glycation end products, *NF-kB* nuclear factor kappa B. References: [1] McGeer et al. (2003), [2] Miklossy et al. (2006), [3] Béraud et al. (2013), [4] Thundyil and Lim (2015), [5] Chao et al. (2014), [6] Fossati and Chiarugi (2007), [7] Liu et al. (2012), [8] Fellner et al. (2013), [9] Farina et al. (2007), [10] Efremova et al. (2015), [11] Davalos et al. (2005), [12] Haynes et al. (2006), [13] Koizumi et al. (2007), [14] Shinozaki et al. (2017), [15] Blank and Prinz (2013), [16] Chapman et al. (2000), [17] Streit et al. (2001), [18] Nayak et al. (2011), [19] Lopategui Cabezas et al. (2014), [20] Shan et al. (2011), [21] Zecca et al. (2008), [22] Santoro et al. (2016), [23] Sasaki et al. (2016), [24] Noelker et al. (2013), [25] Abdelsalam and Safar (2015), [26] Schildknecht et al. (2015), [27] Emmanouilidou et al. (2010), [28] Marques and Outeiro (2012)

#### Biological plausibility

In patients with PD and in MPTP-exposed humans or non-human primates, inflammation remains persistently activated in the nigrostriatal system, even years or decades after removal of the initiating toxicant (McGeer et al. 2003; Miklossy et al. 2006). The inflammatory response involves microgliosis and astrogliosis as well as the infiltration of peripheral CD4^+^ T lymphocytes (Appel 2009; Brochard et al. 2009). Damaged neurons expose cytosolic or nuclear proteins or non-protein molecules, collectively termed as damage-associated molecular patterns (DAMPs), which are capable to initiate and perpetuate an inflammatory response (Béraud et al. 2013; Thundyil and Lim 2015) by activating Toll-like receptors (TLRs) or receptors for advanced glycation end-products (RAGEs) (Chao et al. 2014). Microglial cells are equipped with TLRs such as TLR-2 or TLR-4 that sense targets such as high mobility group box 1 (HMGB1), amyloid beta peptide, or alpha synuclein and hence stimulate activation of nuclear factor kappa B (NF-κB) (Fellner et al. 2013; Fossati and chiarugi 2007; Liu et al. 2012; Santoro et al. 2016). Astrocytes are also able to sense tissue injury via e.g., TLR-3 (Farina et al. 2007). Moreover, neuronal injury promotes astrocyte activation (Efremova et al. 2015). ATP, released by challenged cells, is a prominent non-protein DAMP that stimulates an immune response by purinergic G protein-coupled receptors (P2Y receptors). P2Y receptor activation leads to the migration and polarization of microglial cells (Davalos et al. 2005; Haynes et al. 2006; Koizumi et al. 2007). Reactive microglial cells can in turn modulate astrocyte reactivity, involving P2Y receptors (Shinozaki et al. 2017). Neuron–microglia interactions are also controlled by several chemokines and chemokine receptors (e.g., fractalkine, CD200) and a loss of this control by challenged neurons can trigger microglial reactivity (Blank and Prinz 2013; Chapman et al. 2000; Streit et al. 2001). Activation of glial cells in response to damaged neurons, as well as infiltration of peripheral leukocytes, is not confined to PD, but also observed in other chronic neurodegenerative diseases (Lopategui Cabezas et al. 2014; Nayak et al. 2011).

#### Empirical support

The number of studies describing an explicit causal relationship between damaged DA neurons and the activation of glia is rather limited. In patients with PD, an increase in HMGB1—a protein released upon cell damage that signals danger and promotes neuroinflammation—was found in the *substantia nigra pars compacta* (SNpc) and in cerebrospinal fluid (CSF) (Santoro et al. 2016). In mice treated with MPTP/MPP^+^, administration of HMGB1-neutralizing antibodies partly inhibits DA cell death. The small molecule glycyrrhizin directly binds HMGB1 and reduces MPTP/MPP^+^-dependent DA cell death (Santoro et al. 2016; Sasaki et al. 2016). TLR-4-deficient mice are less vulnerable to MPTP/MPP^+^ intoxication and display a decreased number of reactive activated glial cells compared with MPTP/MPP^+^-treated wild-type animals (Noelker et al. 2013). Inhibition of RAGEs, which are upregulated in the striatum following rotenone exposure, suppresses NF-κB activation, as well as the expression of NF-κB-regulated inflammatory markers such as tumor necrosis factor alpha (TNF-α), inducible nitric oxide synthase (iNOS), and myeloperoxidase (Abdelsalam and Safar 2015). Injection of fractalkine—normally released by damaged neurons—into the SNpc causes microglia activation by binding to CX3C chemokine receptor 1 (CX3CR1). Pre-administration of an anti-CX3CR1 antibody before MPP^+^ injection into the SNpc protects from glial activation (Shan et al. 2011). Intracerebral injection of neuromelanin, a derivative of l-DOPA that accumulates in catecholaminergic neurons, causes an inflammatory activation of glial cells in the rat brain, indicating that degenerating DA neurons are leading to neuroinflammation (Zecca et al. 2008). Furthermore, DA neurons in the process of degeneration signal Ca^2+^ waves, attracting neighboring glial cells, hence contributing to the well-defined accumulation of activated glial cells at the sites of neurodegeneration (Sieger et al. 2012).

#### Inconsistencies

Studies investigating the role of DA neurodegeneration on glial activation often utilize toxicants such as MPTP/MPP^+^ or rotenone. Although no in vivo evidence exists, it cannot be ruled out that these toxicants directly influence glial activation, e.g., by the active conversion of the pro-toxicant MPTP (Schildknecht et al. 2015). A rich body of experimental evidence indicates an outstanding role of extracellular ASYN in the inflammatory activation of glial cells (Hoenen et al. 2016; Lee et al. 2010a, b). However, these factors are not necessarily associated with a degeneration of DA neurons, as ASYN can be excreted by viable neurons (Emmanouilidou et al. 2010; Marques and Outeiro 2012).

### KER 6: relationship between “neuroinflammation” (KE 5) and “degeneration of DA neurons” (KE 4) (Fig. 7)

**Fig. 7:**
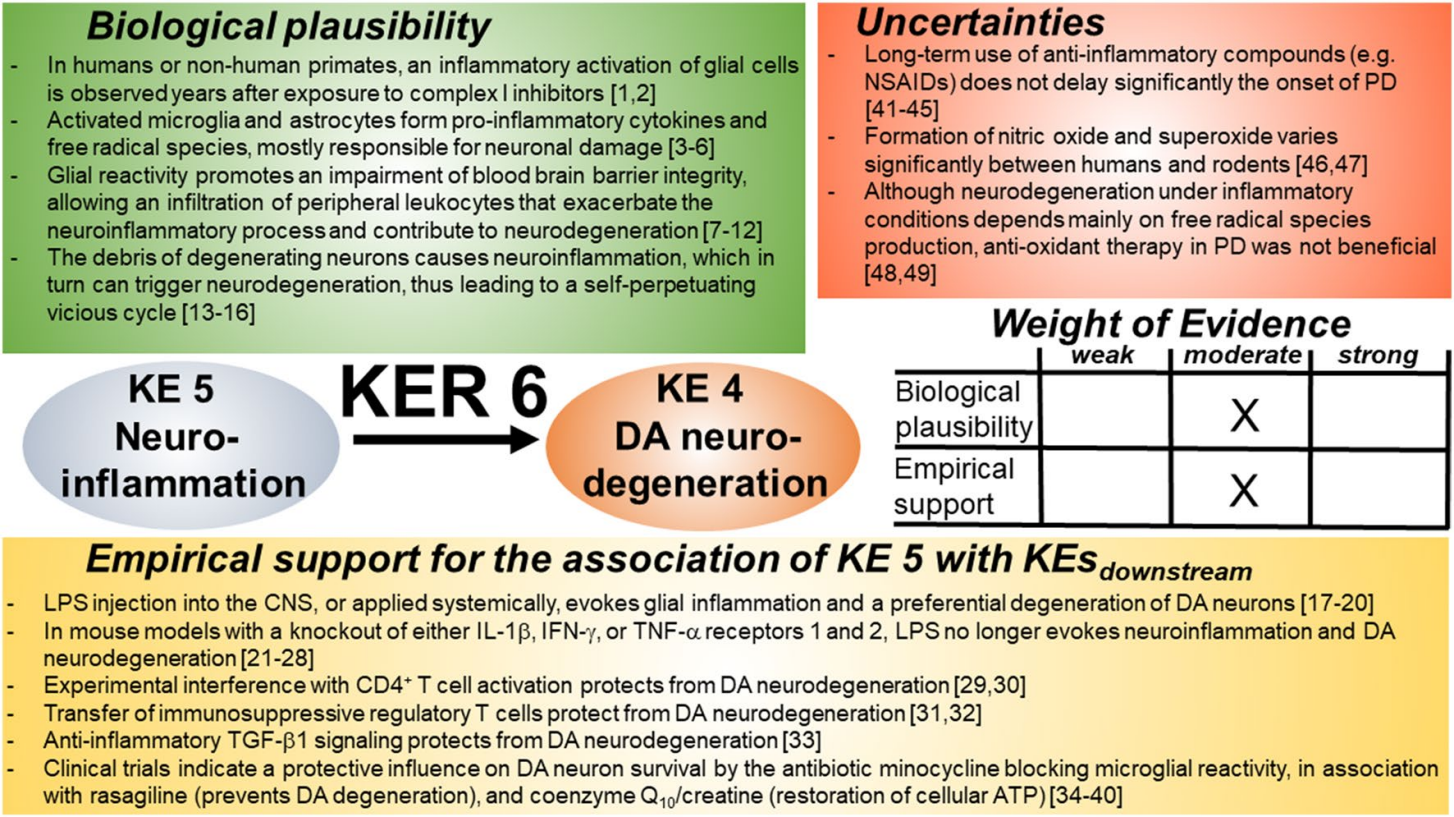
Key event relationship 6 (KER 6), linking DA neuroinflammation (KE 5) and DA neurodegeneration (KE 4). The table shows the result of a qualitative assessment of KER 6 on a 3 point scale (weak, moderate, strong). A causal relationship between neuroinflammation and DA neurodegeneration has been demonstrated. Biological plausibility and empirical support were both rated “moderate”, due to the lack of profound knowledge on the mediators that evoke neurodegeneration. Anti-inflammatory and antioxidant agents could not convincingly demonstrate a neuroprotective potential. *CNS* central nervous system, *DA* dopamine, *IL-1β* interleukin-1β, *IFN-γ* interferon-γ, *TNF-α* tumor necrosis factor α; TGF: transforming growth factor, *NSAID* non-steroidal anti-inflammatory drugs. References: [1] McGeer et al. (2003), [2] Miklossy et al. (2006), [3] Liberatore et al. (1999), [4] Norden et al. (2015), [5] Boka et al. (1994), [6] Dong and Benveniste (2001), [7] Lopez-Ramirez et al. (2014), [8] Pan and Kastin (2002), [9] Banks (2005), [10] Heráandez-Romero et al. (2012), [11] Pott Godoy et al. (2008), [12] Villarán et al. (2010), [13] Hirsch and Hunot (2009), [14] Griffin et al. (1998), [15] Blasko et al. (2004), [16] Barbeito et al. (2010), [17] Herrera et al. (2000), [18] Frank-Cannon et al. (2008), [19] He et al. (2013), [20] Ramsey and Tansey (2014), [21] Tanaka et al. (2013), [22] Mount et al. (2007), [23] Ferger et al. (2004), [24] Leng et al. (2005), [25] Sriram et al. (2002), [26] Sriram et al. (2006), [27] Qin et al. (2007), [28] McCoy et al. (2006), [29] Castaño et al. (2002), [30] Brochard et al. (2009), [31] Reynolds et al. (2007), [32] Laurie et al. (2007), [33] Liu et al. (2016), [34] Faust et al. (2009), [35] Du et al. (2001), [36] Tikka et al. (2001), [37] Wu et al. (2002), [38] Shults (2003), [39] NINDS NET-PD Investigators (2006), [40] NINDS-NET-PD Investigators (2008), [41] Chen et al. (2005), [42] Chen et al. (2003), [43] Hernán et al. (2006), [44] Ton et al. (2006), [45] Etminan et al. (2008), [46] Schildknecht et al. (2005), [47] Hoos et al. (2014), [48] Parkinson Study Group (1993), [49] Shoulson (1998)

#### Biological plausibility

Neuroinflammation, first described by McGeer et al. (1988), encompasses the activation of glial cells (microglia and astrocytes) and is regularly observed in association with chronic neurodegenerative diseases such as PD, Alzheimer’s disease, and Huntington’s disease (Bagyinszky et al. 2017; Falsig et al. 2004, 2006; McGeer and McGeer  2008; Vivekanantham et al. 2015). Both cell types contribute to a pro-inflammatory/neurotoxic environment by releasing cytokines such as interleukin (IL)-1β, TNF-α, or interferon gamma (IFN-γ), mediators such as nitric oxide (^•^NO) or superoxide (^•^O_2_^−^), ceramide, gangliosides, and components of the complement system (Boka et al. 1994; Brown and Bal-Price 2003; Dong and Benveniste 2001; Liberatore et al. 1999; Norden et al. 2015). Neuroinflammation-induced neuronal degeneration depends to a large extent on damage evoked by free radical species such as ^•^NO, ^•^O_2_^−, •^OH, H_2_O_2_, N_2_O_3_, or peroxynitrite, which are actively formed by activated glia (Daiber et al. 2009; Hunot et al. 1996; Knott et al. 2000; Le et al. 1999; Mogi et al. 1994). These free radicals harm neuronal mitochondria and challenge neuronal proteostasis and redox equilibria. Once a certain threshold of radical-mediated damage is reached, neurodegeneration is observed (Chen et al. 2015; Khan et al. 2016). In addition, activated glial cells can alter the integrity of the blood–brain barrier (BBB) and hence allow an infiltration of peripheral immune cells into the CNS (Lopez-Ramirez et al. 2014). Moreover, peripheral TNF-α and IL-1α can traverse the BBB (Banks 2005; Pan and Kastin 2002). As a consequence of their passage, chronic peripheral inflammation can contribute to the selective demise of nigrostriatal DA neurons in the brain (Hernández-Romero et al. 2012; Pott Godoy et al. 2008; Villarán et al. 2010).

Neuronal injury/death triggers neuroinflammation (see KER 5), which in turn can lead to neuronal degeneration, contributing to a self-perpetuating vicious circle, which is assumed to be a key element in the pathogenesis of several neurodegenerative diseases including PD (Barbeito et al. 2010; Blasko et al. 2004; Griffin et al. 1998; Hirsch and Hunot 2009).

#### Empirical support

Nigrostriatal neurodegeneration can be evoked by stereotaxic injection or systemic application of lipopolysaccharide (LPS), a known activator of microglia (Frank-Cannon et al. 2008; He et al. 2013; Herrera et al. 2000; Ramsey and Tansey 2014).

Strategies to dampen neuroinflammation and protect DA neurons have either focused on inhibiting the pro-inflammatory (M1) phenotype of microglia and/or on supporting their anti-inflammatory activation state (M2) (Hernández-Romero et al. 2008; Lecca et al. 2015; Lu et al. 2000; Moehle and West 2015; Moon et al. 2009; Pisanu et al. 2014; Roy et al. 2012; Wu et al. 2002). In comparison to control mice, intra-nigrostriatal injection of LPS largely failed to initiate a sustained neuroinflammatory response in an IL-1β knockdown mouse model that exhibits significantly less DA neurodegeneration (Tanaka et al. 2013). In an MPTP/MPP^+^ model, IFN-γ depletion completely prevents microglial activation and protects from the loss of nigrostriatal DA neurons (Mount et al. 2007). Deletion of TNF-α confers only a partial protection from MPTP/MPP^+^-dependent neuroinflammation and DA neurodegeneration (Ferger et al. 2004), while a double knockout mouse (Leng et al. 2005) of TNF-α receptors 1 and 2 exhibits an almost complete protection from MPTP/MPP^+^-dependent glial activation and DA neurodegeneration (Sriram et al. 2002, 2006). Comparable protection in this TNF-α receptor double knockout mouse was also reported when LPS was applied instead of MPTP (Qin et al. 2007). Blocking TNF-α by expression of TNF-α inhibitor protein protects from DA neuron loss in animal models of PD (McCoy et al. 2006).

In mixed neuron/glia co-cultures, pretreatment with anti-inflammatory TGF-β1 prevents from neurodegeneration evoked by MPP^+^ (Liu et al. 2016). Genetic silencing of the TGF-β receptor 1 in microglia reverses this protective effect, indicating a significant role of pro-inflammatory glial activation in the observed degeneration of neurons (Liu et al. 2016). Another example is the PPAR-γ agonist MDG548 that decreases NF-κB activation in microglia evoked by LPS (Lecca et al. 2015). When mice are exposed to MPTP instead of LPS, MDG548 reduces microglial activation and protects from DA neurodegeneration (Lecca et al. 2015).

Other strategies interfering with the infiltration of peripheral CD4^+^/CD8^+^ T lymphocytes, which was reported as a contributing factor of DA neurodegeneration (Brochard et al. 2009; Appel 2009; Stone et al. 2009), also revealed neuroprotection. The corticosteroid dexamethasone, by acting as an inhibitor of T-cell infiltration, dampens glial activation and DA neurodegeneration (Castaño et al. 2002). In a MPTP/MPP^+^ model, a mutation in the functional receptor of CD4^+^ T cells protects from DA neurodegeneration (Brochard et al. 2009). The adoptive transfer of immunosuppressive CD4^+^/CD25^+^ regulatory T cells was sufficient for the protection from DA neuronal death (Laurie et al. 2007; Reynolds et al. 2007). Besides these experimental models, current clinical trials involving patients with PD, strongly suggest minocycline, an inhibitor of microglial reactivity (Du et al. 2001; Faust et al. 2009; Schildknecht et al. 2011; Tikka et al. 2001; Wu et al. 2002), as a promising agent for the protection of nigrostriatal DA neurons when used in combination with other therapies such as antioxidants or MAO-B inhibitors (Galpern and Cudkowicz 2007; Matthews et al. 1999; NINDS NET-PD Investigators 2006, 2008; Shults et al. 1997, 1999, 2002; Shults 2003, 2004; Yang et al. 2009).

#### Inconsistencies

The majority of studies focusing on the contribution of pro-inflammatory mediators such as IL-1β, TNF-α, or IFN-γ were performed in MPTP models. Hence, in addition to the inflammatory response, MPP^+^-dependent mitochondrial inhibition and ROS formation were still present in these studies. Mice with quiescent microglia are still susceptible to MPTP toxicity (Kinugawa et al. 2013), indicating a rather minor contribution of inflammation to the observed neurodegeneration in the MPTP models. Studies involving LPS injections for the induction of inflammation were almost exclusively conducted in rodents. In comparison to the situation in humans, rodents display greater amounts of ^•^NO and ^•^O_2_^−^ upon inflammatory activation (Bachschmid et al. 2005; Hoos et al. 2014; Schildknecht et al. 2004, 2005), indicating that these radical species contribute to a larger extend to neurodegeneration in rodents compared to the situation in humans. This concept received substantial support by the outcome of clinical studies involving antioxidant therapy over extended periods of time that exhibited no signs of a significant delay in disease progression (Chen 2003, 2005; Etminan et al. 2008; Hernán et al. 2006; Ton et al. 2006; Parkinson Study Group 1993; Shoulson 1998).

### KER 7: relationship between “mitochondrial dysfunction” (KE 2) and “degeneration of DA neurons” (KE 4) (Fig. 8)

**Fig. 8:**
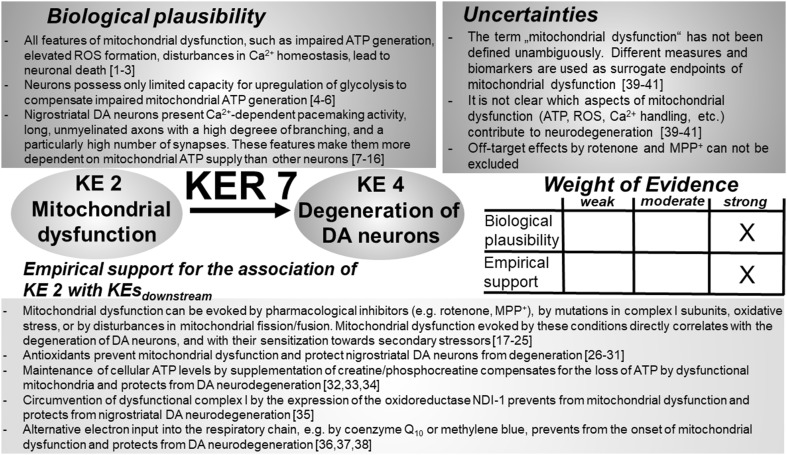
Key event relationship 7 (KER 7), linking mitochondrial dysfunction (KE 2) and DA neurodegeneration (KE 4). The table shows the result of a qualitative assessment of KER 7 on a 3 point scale (low, moderate, strong). The literature is currently lacking a generally accepted definition of mitochondrial dysfunction. There is currently no consensus on the contribution of individual processes (e.g. mitochondrial membrane potential loss, ROS formation, drop in ATP formation, release of pro-apoptotic factors, etc.) to overall mitochondrial dysfunction nor a quantitative assessment of these processes for threshold definition. However, for some endpoints, semi-quantitative information is available. Notably, the support that KER 7 prevails over KER 3 and KER 4 is limited to few experimental situations, and human evidence has not been established. *ATP* adenosine triphosphate, *ROS* reactive oxygen species, *MPP* ^*+*^ 1-methyl-4-phenylpyridinium, *NDI-1* single subunit NADH dehydrogenase of *S. cerevisiae*. References: [1] Bose and Beal (2016), [2] Banerjee et al. (2009), [3] Subramaniam and Chesselet (2013), [4] Herrero-Mendez et al. (2009), [5] Almeida et al. (2001), [6] Almeida et al. (2004), [7] Nedergaard et al. (1993), [8] Guzman et al. (2009), [9] Chan et al. (2007), [10] Surmeier et al. (2011), [11] Surmeier and Schumacker (2013), [12] Bolam and Pissadaki (2012), [13] Matsuda et al. (2009), [14] Pissadaki and Bolam (2013), [15] Pacelli et al. (2015), [16] Schildknecht et al. (2017), [17] Chan et al. (1991), [18] Fabre et al. (1999), [19] Hasegawa et al. (1990), [20] Nicklas et al. (1985), [21] Przedborski et al. (1996), [22] Sherer et al. (2003), [23] Sherer et al. (2007), [24] Marella et al. (2008), [25] Ekstrand et al. (2007), [26] Du et al. (2001), [27] Choi et al. (2014), [28] Hajieva et al. (2009), [29] Chen et al. (2015), [30] Marella et al. (2008), [31] Wen et al. (2011), [32] Beal et al. (1998), [33] Adhihetty and Beal (2008), [34] Cunha et al. (2014), [35] Seo et al. (1998, 2000, 2002), [36] Shults et al. (2002), [37] Moon et al. (2005), [38] Wen et al. (2011), [39] Wang et al. (2012), [40] Leist et al. (1998), [41] Leist et al. (1997)

#### Biological plausibility

KER 7 is an extraordinary element in the present AOP inasmuch it circumvents KE 3. Impaired proteostasis (KE 3) is observed under conditions of moderate and chronic inhibition of complex I. In response to an instant and complete inhibition of complex I by high concentrations of rotenone or MPP^+^, the instant termination of ATP supply can lead to a rapid (1–2 h) degeneration without significant involvement of impaired proteostasis. Incorporation of these time- and concentration-dependent differences in neurodegeneration upon complex I inhibition was the rationale to specify KER 7.

Mitochondria serve as the main source of ATP in eukaryotic cells, and they are vitally involved in the regulation of cellular Ca^2+^ homeostasis (Baughman et al. 2011; Brini et al. 2014; Calì et al. 2014; De Stefani et al. 2011) as well as in apoptotic processes (Charan et al. 2014; Hu et al. 2015; Liu et al. 2015; Rasheed et al. 2017). Mitochondrial dysfunction is characterized by dysfunctional cellular Ca^2+^ handling (Orrenius et al. 2003), reduced mitochondrial ATP levels, and increased ROS (Banerjee et al. 2009; Bose and Beal 2016; Subramaniam and Chesselet 2013). In contrast to other cell types, neurons have only a moderate capacity to upregulate their rate of glycolysis upon inhibition of mitochondria (Almeida et al. 2001, 2004; Herrero-Mendez et al. 2009). Therefore, they are more vulnerable towards dysfunctional mitochondria than other cell types. Among the different neuronal types, nigrostriatal DA neurons display a preferential sensitivity towards complex I inhibition (Betarbet et al. 2000; Jackson-Lewis et al. 1995) as a consequence of a set of unique intrinsic features. First, nigrostriatal DA neurons possess autonomous pacemaking activity, relying on L-type Ca^2+^ channel (Ca_V_1.3)-dependent Ca^2+^ influx for membrane depolarization (Chan et al. 2007; Guzman et al. 2009; Nedergaard et al. 1993). The relevance of Ca^2+^-dependent pacemaking as a sensitizing factor becomes evident in comparison with DA neurons of the ventral tegmental area (VTA). These are significantly less sensitive to complex I inhibition, and they differ from nigrostriatal DA neurons by their reliance on extracellular Na^+^ for pacemaking (Khaliq and Bean 2010). The constant influx of extracellular Ca^2+^ represents an energy- demanding strategy (Surmeier et al. 2011; Surmeier and Schumacker 2013). The energy balance of nigrostriatal DA neurons, and hence their dependence on proper mitochondrial function, is furthermore challenged by their unique architecture (Bolam and Pissadaki 2012; Matsuda et al. 2009) comprising long unmyelinated axons and higher numbers of energy-consuming synapses, compared with catecholaminergic neurons of other brain regions (Pacelli et al. 2015; Pissadaki and Bolam 2013). As a consequence, total cell surface and the energy required to maintain the membrane potential is higher in nigrostriatal DA neurons (Pacelli et al. 2015; Bolam and Pissadaki 2012; Brichta and Greengard 2014). In comparison with neurons of other brain areas, mitochondria of nigrostriatal DA neurons can hence barely meet the energy requirement of the cell, even under normal conditions. It becomes apparent that even moderate impairments in mitochondrial function can lead to a preferential damage and demise of nigrostriatal DA neurons, while other neuronal populations under the same conditions are still spared. An additional sensitizing factor is the presence of DA, which pre-disposes neuronal cells to oxidative stress and renders ASYN particularly cytotoxic (Pacelli et al. 2015; Schildknecht et al. 2009, 2013, 2017).

#### Empirical support

The experimental support for the direct relationship between mitochondrial dysfunction and the degeneration of nigrostriatal DA neurons is based on observations made with neuronal cell cultures and with genetically modified in vivo models. Mitochondrial dysfunction can be initiated by complex I inhibitors that prevent mitochondrial ATP generation and concomitantly stimulate mitochondrial ROS formation (Chan et al. 1991; Fabre et al. 1999; Hasegawa et al. 1990; Nicklas et al. 1985; Przedborski et al. 1996). Alternative experimental means to evoke mitochondrial dysfunction are e.g., transfer of mtDNA from patients with PD into mtDNA-free cells (cybrids) (Marella et al. 2008; Sherer et al. 2003, 2007) or knockdown of the regulator of mitogenesis Tfam (Ekstrand et al. 2007). In all of these examples, the advent of mitochondrial dysfunction was directly correlated with the demise of neurons, and an elevated sensitivity of neurons harboring dysfunctional mitochondria towards secondary stressors. The degeneration of DA neurons is prevented by treatment with antioxidants (Chen et al. 2015; Choi et al. 2014; Hajieva et al. 2009; Sherer et al. 2003, 2007). To exemplify the present AOP, mitochondrial dysfunction can be evoked by application of complex I inhibitors such as MPTP/MPP^+^ or rotenone both in vitro and in vivo. Such complex I inhibitor-mediated mitochondrial dysfunction is directly correlated with the dysfunction of nigrostriatal DA neurons (Hantraye et al. 1993; Langston et al. 1999; Moratalla et al. 1992; Varastet et al. 1994). Experimental expression of the inhibitor-insensitive complex I surrogate NDI-1, either in neuronal cell cultures or in vivo by unilateral injection of adeno-associated virus into the nigrostriatal system (Marella et al. 2008; Sherer et al. 2003, 2007) protects against complex I inhibitor-dependent mitochondrial dysfunction and prevents the demise of nigrostriatal DA neurons. Complex I-independent electron input into the respiratory chain, e.g., by application of methylene blue (Wen et al. 2011) or coenzyme Q_10_ (Beal et al. 1998), reduces mitochondrial dysfunction and protects from DA neurodegeneration. These examples illustrate that protection from the loss of mitochondrial ATP, or from conditions of oxidative stress, i.e., features of mitochondrial dysfunction, are effective means to prevent the demise of nigrostriatal neurons.

#### Uncertainties

Mitochondrial dysfunction comprises a series of adverse processes such as the decline of the mitochondrial membrane potential, opening of the mtPTP, elevated ROS formation, or the release of cytochrome *c* (Gandhi et al. 2009; Heo et al. 2012; Irrcher et al. 2010; Leist et al. 1998; Pöltl et al. 2012; Toulorge et al. 2016; Wang et al. 2012). Currently, there is no consensus on how many of these changes need to occur to meet the criteria for mitochondrial dysfunction. A decline in ATP generation and elevated ^•^O_2_^−^ formation are the two main consequences of complex I inhibition (Lambert and Brand 2004; Schildknecht et al. 2009). Although experimental restoration of ATP and management of elevated ROS by antioxidants have been suggested as protective means, the respective quantitative contribution of ATP and ROS to the observed neurodegeneration has not been fully addressed in the literature. Mitochondrial dysfunction leads to oxidative stress, but oxidative stress in turn also leads to mitochondrial dysfunction (Hasegawa et al. 1990; Jana et al. 2011; Khan et al. 2005). Thus, empirical support based on antioxidants can be ambiguous. In KER 7, it is assumed that KE 2 directly leads to KE 4 and KE 5, especially at high intensities of insult. However, it is unclear whether such conditions are found in humans exposed to toxicants.

### KER 8: relationship between the “degeneration of DA neurons” (KE 4) and the onset of “parkinsonian motor deficits” (AO) (Fig. 9)

**Fig. 9:**
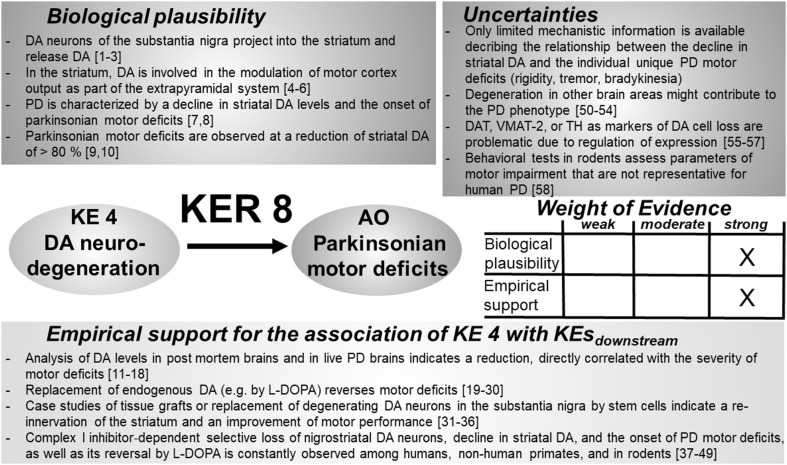
Key event relationship 8 (KER 8), linking DA neurodegeneration (KE 4) and parkinsonian motor deficits (AO). The table shows the result of a qualitative assessment of KER 8 on a 3 point scale (weak, moderate, strong). Literature provides strong evidence for a causal correlation between the levels of striatal dopamine and the onset of parkinsonian motor deficits. These correlations can be observed in MPTP exposed rodents, primates, including humans, and in human PD. A potential contribution of other brain areas, respectively, their demise, to parkinsonian motor deficits, was only inadequately investigated so far. *DA* dopamine, *PD* Parkinson’s disease, *l* *-DOPA* l-3,4-dihydroxyphenylalanine, *DAT* dopamine transporter, *VMAT-2* vesicular monoamine transporter 2, *TH* tyrosine hydroxylase. References: [1] Lynd-Balta and Haber (1994a), [2] Lynd-Balta and Haber (1994b), [3] Joel and Weiner (2000), [4] Alexander et al. (1986), [5] Obeso et al. (2008a), [6] Blandini et al. (2000), [7] Ehringer et al. (1960), [8] Bernheimer et al. (1973), [9] Koller (1992), [10] Kirik et al. (1998), [11] Earle (1968), [12] Lloyd et al. (1975), [13] Benamer et al. (2000), [14] Rakshi et al. (1999), [15] Lin et al. (2014), [16] Pirker (2003), [17] Rinne et al. (1995), [18] Tissingh et al. (1998), [19] Lloyd et al. (1975), [20] Yam et al. (1998), [21] Gilmour et al. (2011), [22] Heimer et al. (2002), [23] Papa et al. (1999), [24] Hutchinson et al. (1997), [25] Levy et al. (2001), [26] Parkinson Study Group (1993), [27] Pålhagen et al. (1998), [28] Pålhagen et al. (2006); [29] Parkinson Study Group (1996), [30] Olanow et al. (2008), [31] Widner et al. (1992), [32] Kordower et al. (1998), [33] Kordower et al. (1995), [34] Mendez et al. (2008), [35] Schumacher et al. (2000), [36] Ben-Hur et al. (2004), [37] Bezard et al. (2001), [38] Blesa et al. (2012), [39] Mitchell et al. (1989), [40] Filion and Tremblay (1991), [41] Bergman et al. (1990), [42] Aziz et al. (1991), [43] Porras et al. (2012), [44] Jenner (2008), [45] Bédard et al. (1986), [46] Clarke et al. (1987), [47] Langston et al. (2000), [48] Smith et al. (2003), [49] Kuoppamäki et al. (2007), [50] Seniuk et al. (1990), [51] Muthane et al. (1994), [52] Moratalla et al. (1992), [53] Snow et al. (2000), [54] Forno et al. (1986), [55] Petzinger et al. (2006), [56] Jakowec et al. (2004), [57] Rothblat et al. (2001), [58] Meredith and Kang (2006)

#### Biological plausibility

DA neurons of the substantia nigra project into the striatum, where they release DA (Joel and Weiner 2000; Lynd-Balta and Haber 1994a, b). The loss of nigrostriatal DA neurons observed in PD leads to a reduction in striatal DA levels (Bernheimer et al. 1973; Ehringer et al. 1960). All PD forms are characterized by the loss of striatal DA, which is directly correlated with the onset of PD motor symptoms (Ehringer et al. 1960). Striatal DA is a main regulator of motor output from the cortex to the periphery. Basal ganglia modulate motor output information that is looped back via the thalamus to the motor output cortex (Alexander et al. 1986; Blandini et al. 2000; Obeso et al. 2008a, b). A decline in striatal DA leads to disturbances in this feedback loop and reflects key parkinsonian symptoms such as rigidity, bradykinesia, and tremor (Bain 2007; Jankovic 2008; Rodriguez-Oroz et al. 2009). An experimental knockdown of tyrosine hydroxylase as the key enzyme in catecholamine synthesis leads to reduced motor coordination (Korner et al. 2015), while a hyperdopaminergic tone, evoked by genetic deletion of the DA transporter, results in motor hyperactivity (Gainetdinov et al. 1999). Characteristic PD-associated motor deficits are usually observed at a reduction of striatal DA by ca. 80% (Kirik et al. 1998; Koller 1992).

#### Empirical support

Experimental support for a causal relationship between the loss of nigrostriatal DA neurons and the onset of parkinsonian motor deficits comes from patients with PD, humans accidentally exposed to MPTP, in vivo studies with rodents and non-human primates, and from in vitro models.

Analysis of brains from patients with PD reveals a significant reduction of striatal DA that correlates with the degeneration of nigrostriatal DA neurons (Earle 1968; Lloyd et al. 1975). Live assessment of DA neuron content in patients with PD indicates a causal correlation between nigrostriatal DA content and the severity of PD motor deficits (Benamer et al. 2000; Lin et al. 2014; Pirker 2003; Rakshi et al. 1999; Rinne et al. 1995; Tissingh et al. 1998). Substitution of endogenous striatal DA by l-DOPA leads to improved motor performance (Gilmour et al. 2011; Heimer et al. 2002; Hutchinson et al. 1997; Levy et al. 2001; Lloyd et al. 1975; Papa et al. 1999; Yam et al. 1998). Elevation of endogenous striatal DA, by application of inhibitors targeting its degradation enzyme MAO-B, is also correlated with improved motor performance (Pålhagen et al. 1998, 2006; Olanow et al. 2008; Parkinson Study Group 1993, 1996, 2002; Rascol et al. 2005). Case reports further indicate the re-innervation of the striatum with projections of transplanted DA neurons, a restoration of striatal DA levels, and a subsequent improvement of motor performance (Ben-Hur et al. 2004; Kordower et al. 1995, 1998; Mendez et al. 2008; Schumacher et al. 2000; Widner et al. 1992).

Non-human primates represent a model, highly reflective for the situation in humans with respect to brain architecture and DA motor deficits. Studies with MPTP-exposed monkeys reveal a correlation between striatal DA, nigrostriatal DA neuron numbers, and the onset of a PD motor phenotype (Bezard et al. 2001). Similar to the situation in humans, a reduction of striatal DA by ca. 80% leads to the manifestation of PD motor deficits (Aziz et al. 1991; Bergman et al. 1990; Blesa et al. 2012; Filion and Tremblay 1991; Mitchell et al.1989; Porras et al. 2012). Supplementation of endogenous DA by l-DOPA application in these models reverses the loss of motor output performance (Bédard et al. 1986; Clarke et al. 1987; Jenner 2008; Kuoppamäki et al. 2007; Langston et al. 2000; Smith et al. 2003).

In rodents, systemic administration of rotenone leads to loss of striatal DA and DA neurons; this loss is associated with the onset of motor deficits, reminiscent of those motor impairments observed in patients with PD (Alam and Schmidt 2002, 2004; Cannon et al. 2009; Fleming et al. 2004; Höglinger et al. 2003a, b). In mice, MPTP is the most widely applied experimental PD toxicant. It provides results comparable to those obtained from rotenone experiments in rats (Alvarez-Fischer et al. 2008; Fornai et al. 2005; Gibrat et al. 2009; Hung and Lee 1996; Petroske et al. 2001; Rozas et al. 1998). Application of l-DOPA, inhibition of endogenous DA degradation by MAO-B inhibitors, deep brain stimulation, and transplantation of precursor cells into the nigrostriatal system all lead to a restoration of striatal DA content and an improvement of motor performance (Altarche-Xifro et al. 2016; Kong et al. 2015; Shaw et al. 2010; Schierle et al. 1999; Shin et al. 2009).

#### Inconsistencies

Striatal DA is a key modulator of extrapyramidal motor output control. Although a close correlation between striatal DA and the onset of motor deficits is apparent, the specificity of motor abnormalities observed in PD has not been fully explained. Neuronal loss in PD or in MPP^+^/rotenone-treated animals is not confined to the nigrostriatal system. Other areas such as the locus coeruleus also undergo neurodegeneration (Forno et al. 1986; Moratalla et al. 1992; Muthane et al. 1994; Seniuk et al. 1990; Snow et al. 2000). It cannot be excluded that additional brain regions might significantly contribute to the parkinsonian motor phenotype. In subacute treatment schemes (rotenone, MPTP), a significant, sometimes complete, recovery of motor deficits can be observed (Petroske et al. 2001). For the assessment of DA neuron numbers, DA markers such as TH, DAT, and VMAT-2 are often employed using western blot, immunohistochemical staining, and polymerase chain reaction (PCR). However, the expression levels of these targets can be transiently regulated and might, therefore, provide misleading information on the survival of DA neurons (Jakowec et al. 2004; Petzinger et al. 2006; Rothblat et al. 2001). For the assessment of motor performance in rodents, a variety of different behavioral assays emerged in the course of recent decades. The parameters assessed in these assays are not directly representative for those features observed in human PD (Meredith and Kang 2006). In addition, it has not been established for all endpoints whether deficits can be fully rescued by l-DOPA or DA agonists.

## AOP uncertainties and evaluation in an overall context

### Use of example compounds for the AOP

Per definition, AOPs are compound agnostic and consequently include no ADME considerations. However, for the assembly of AOPs, and in particular for the empirical support of KERs, the behavior of model compounds plays a significant role and is important for the assessment of plausibility of an AOP. Model toxicants show a distinct toxicokinetic behavior that needs to be taken into account for the evaluation of the consistency of the AOP. In this context, the AOP presented herein relies very heavily on its exemplification by two model toxicants: rotenone and MPTP. For MPTP, the evaluation of the dose and KE sequence (response–response) consistency is particularly difficult, because: (1) MPTP needs to be enzymatically activated into the active toxicant MPP^+^ by brain glial cells; (2) once the active metabolite MPP^+^ has been formed, and the MIE occurred, the following KE can be initiated within a very narrow time window. Moreover, information on its uptake, conversion rates, transport within the brain, and its excretion, can have a significant influence on the AO, and such toxicokinetic factors might explain the intra- (mouse strains) and inter-species (rodents versus non-human primates) differences observed.

Altogether, if data are combined from multiple published studies, both rotenone and MPTP show a good response–response and temporal concordance. However, it remains unclear for MPTP, whether there are doses that trigger only early KEs without activating the AO (Table 3). Although a wide variety of other complex I inhibitors have been described in the literature, these compounds were only rarely applied in studies linking information from isolated mitochondria, cell culture, and in vivo models.

**Table 3:** Response–response and temporal concordance table for rotenone and MPTP/MPP^+^

Rotenone concentration	KE 1 inhibition of C I	KE 2 mitochondrial dysfunction	KE 3 impaired proteostasis	KE 4 degeneration of DA neurons	AO Parkinsonian motor deficits	
5–10 nM in vitro [1]	[+] 4–72 h [1]	[+] 4–72 h [4]	[+] 24 h [3]	–	–	
20–30 nM ex vivo, rat brain concentration [4–5–2–6]	[++] 4–72 h (4–5)	[++] 4–72 h [4–5]	[++] 24 h [3–2–6]	[++] 5 weeks [2–6]	[+++] 5 weeks [2–6]	
100 nM in vitro [4]	[+++] 4–72 h [4]	[+++] 4–72 h [4]	[+++] 24 h [3]	Above the maximum tolerated dose in vivo [2-6]	Above the maximum tolerated dose in vivo [2-6]	

MPTP administered dose	MPP^+^ brain concentration	KE 1 inhibition of C I	KE 2 Mitochondrial dysfunction	KE 3 impaired proteostasis	KE 4 degeneration of DA neurons of nigrostriatal pathway	AO Parkinsonian motor symptoms
1 mg/kg sc infusion [1]	–	–	–	[+] 4 weeks[1]	[+] 4 weeks [1]	No effect
5 mg/kg sc infusion [1]	–	–	–	[++] 4 weeks [1]	[++] 4 weeks [1]	[+++] 4 weeks [1]
20–30 mg/kg sporadic ip injection (4 times every 2 h) [2, 1]	47 μM [2] 12 μM [1]	[+++] 4 h [2]	[+++] 4 h [2]	[+++] 4 weeks [1]	[+++] 1–4 weeks [2,1]	[+++] 4 weeks [1]

Overview on the sequential concentration and time-dependent initiation of the individual key events. +, low severity score; ++, intermediate severity score; +++, high severity score. References: [1] Choi et al. (2008), [2] Betarbet et al. (2006), [3] Chou et al. (2010), [4] Barrientos and Moraes (1999), [5] Okun et al. (1999), [6] Betarbet et al. (2000), [7] Fornai et al. (2005), [8] Thomas et al. (2012)

### Link of the MIE to the downstream events of the AOP

The MIE involves binding of an inhibitor to complex I, leading to the inhibition of complex I as KE 1. This appears as a rather unambiguous biochemical event but on closer inspection, these events are highly complex and a complete description and measurement in the context of the overall AOP is still missing. Mapping of the exact binding site would require confirmation from several independent laboratories, and the same applies to the type of changes in the mitochondrial respiratory chain that have a direct influence on downstream events of the AOP. In this context, it is important to stress again that complex I inhibition not only results in a reduction of mitochondrial electron transport, but also in an increase in superoxide formation. It is not clear whether all complex I inhibitors trigger these events at similar potency and efficacy ratios, and thus, whether all complex I inhibitors would lead to a similar activation of KE 2 and the AO. Low concentrations of complex I inhibitors were reported to evoke elevated superoxide formation by isolated mitochondria without significant influence on mitochondrial ATP generation. This aspect is of importance in light of observations indicating that in vivo models best reflect molecular events typical for PD following low-dose and chronic inhibitor infusion (Crawley 1999). These examples illustrate the necessity for a quantitative assessment of the respective contribution of declined mitochondrial ATP generation and elevated mitochondrial superoxide formation upon complex I inhibition to neurodegeneration in models of different complexity (isolated mitochondria, cell cultures, in vivo models). The involvement of superoxide formation deserves even more attention, considering reports that illustrate a negative feedback inhibition of complex I by superoxide. As a consequence of the current inadequate knowledge on the roles of ATP and superoxide, defined no-effect levels for complex I inhibitors have not yet been established.

### Potential branching of the AOP downstream of mitochondrial events

Impaired proteostasis (KE 3) comprises several complex biological processes involved in the formation, localization, and removal of proteins, in protein assembly (up to organelles), and in the removal of misfolded protein aggregates. Disturbance of proteostasis may follow different patterns. For example, axonal transport may be disturbed, while processes such as proteasomal degradation and removal of aggregated proteins function well. Alternatively, CMA may be disturbed, while other processes such as axonal transport maintain their functionality. At present, the literature provides mainly information on isolated aspects of KE 3, such as UPS and CMA activities. In the future, studies that focus on the particular role of these events in the AOP and compare different proteostasis processes would be desirable. At present, the KER linking mitochondrial dysfunction (KE 2) and impaired proteostasis (KE 3) and the KER linking impaired proteostasis (KE 3) and degeneration of DA neurons (KE 4) are characterized by a lack of quantitative threshold data from independent laboratories and complementary model systems. Such quantitative data would allow a sharper definition of the relationship between the respective KEs (when KE_up_ triggers KE_down_). The majority of information on impaired proteostasis included in the present AOP is based on studies with an explicit focus on the PD-associated protein ASYN, but not on the global cellular imbalance between the formation and degradation of misfolded proteins.

A further notable aspect is that KER 7 provides a direct link between mitochondrial dysfunction (KE 2) and the degeneration of DA neurons (KE 4), hereby circumventing KE 3 (impaired proteostasis). The rationale for KER 7 is based on observations indicating a direct link between KE 2 and KE 4 under conditions of severe mitochondrial dysfunction, e.g., evoked by an almost complete inhibition of complex I.

### Feed-forward loop involving neuroinflammation

In contrast to the standard unidirectional chain of events requested by OECD guidelines for the organization of an AOP, the present AOP included a positive feed-forward loop involving neuroinflammation (KE 5). The inclusion of neuroinflammation as an independent KE was necessary and justified, as neuroinflammation alone is sufficient to evoke DA neurodegeneration, and neuroinflammation can be triggered by complex I inhibitors. However, quantitative information on the extent and type of neuroinflammation is currently not available. Furthermore, significant species differences with respect to the quantitative contribution of neuroinflammation and the mediators involved in DA neurodegeneration have been reported. In rodents, MPTP/MPP^+^-evoked neuroinflammation is mainly self-limiting after the acute phase of neurodegeneration, while it persists for years and even decades in monkey models and in humans. Despite serious attempts, it has not been possible to determine whether neuroinflammation occurs before or after neurodegeneration. The most likely reason for this is that low levels of neurodegeneration trigger neuroinflammation, and that neuroinflammation then triggers more neurodegeneration, so that these two events form a self-perpetuating vicious cycle (Schildknecht et al. 2017). The feed-forward loop depicted in the AOP is the best representation of this pathogenic situation. In this context, it is important to note that other feed-forward loops play a role in this AOP. They are somewhat less prominent and have not been graphically represented, as they could be considered as modulatory events that are covered in the text descriptions. For instance, a self-amplifying feed-forward loop includes mutual interactions between mitochondrial dysfunction and impaired proteostasis. Experimental evidence suggests that disturbed proteostasis affects mitochondrial function (Sherer et al. 2002). Such effects require more attention, if this AOP is used as basis for construction of a quantitative AOP or for a systems biology model.

### Human disease symptom as AO

It is important to distinguish between PD (a complex human disease with multiple symptoms and most likely multiple etiologic factors) and parkinsonian motor deficits (one distinct and sharply circumscribed feature of PD, but also of poisoning). A disease cannot be an AO, but a defined defect, just as a certain type of motor disturbance can be an AO. The type of motor dysfunction of interest here is characterized by problems with movement initiation and termination, much more than problems with the movement process as such. It is called here ‘parkinsonian’ as it is observed in PD, but similar defects are also found in other, not PD-related situations. PD is considered as a multifactorial disease. Consequently, the phenotype (pattern of symptoms) of individuals affected by parkinsonian motor deficits can vary significantly. Therefore, the AO of the present AOP does not cover the entire spectrum of PD-associated phenotypes. Other AOP would need to cover these, and they may have entirely different MIE and KEs.

### Overall judgment and incorporation in larger networks

One of the cardinal aspects in the evaluation of an AOP is the question whether activation of downstream KEs, or the AO, can be prevented by experimental interference with an upstream KE. The essentiality evidence is important to assess the relative level of confidence of the AOP and, when considering all the elements on a rank order, it is secondary only to the biological plausibility for the KERs. Evidence of essentiality was considered “strong” if direct evidence was available in the literature from specifically designed experimental studies illustrating that KE_up_ was essential for at least one KE_down_ or the AO. Evidence was considered “moderate” if only indirect observations were available in the literature, illustrating that KE_up_ was essential for at least one KE_down_ or the AO. In this case, the experiments could not directly address the essentiality of KE_up_, but instead e.g., a modulatory factor for KE_up_. In other cases, a KE_up_ was directly modified, but the tool used was somehow ambiguous or only shown to work by correlation (not intervention). For instance, different approaches to block events associated with (or modulating) neuroinflammation by knockout of mediators (e.g., TNF-α) or by enzyme inhibition (e.g., cyclooxygenase) were found to prevent neurodegeneration. Evidence was considered “weak” if the available observations were contradicting, or if the approaches chosen were indirect and could be interpreted in different ways. Description of the elements supporting essentiality are here embedded in the text for empirical support, a summary of the key evidence and the trend for their weight is reported in Table 2. According to this rule-set, the evidence for essentiality of KEs 1, 2, and 4 was considered “strong”, while the evidence for essentiality of KEs 3 and 5 was considered “moderate” (Table 2).

Although an AOP is an independent unit of information, networks of AOP formed by overlapping KEs are envisaged as a foundation for toxicological evaluations in the future. Quantitative information provided by the individual AOPs of an integrated network could provide a valuable basis for in silico modeling of cellular networks. For this reason, it is important to consider where the present AOP overlaps with other AOPs. KE 2 (mitochondrial dysfunction) was identified in nine other AOPs, where it plays different roles compared with the one described herein. KE 5 (neuroinflammation) is shared by three other AOPs (Fig. 1). Notably, none of these other AOPs has the same AO as described herein.

## Regulatory and scientific context

One of the primary objectives of the AOP framework is knowledge assembly, i.e., gathering information, attained through scientific research by subject-matter experts, for its accessibility to regulators during the decision-making process. To avoid any misunderstanding, it is important to clarify misconceptions about AOPs: (1) risk assessments cannot be based on AOPs alone, as they do not address exposure and toxicokinetic issues. AOPs inform on a potential hazard, and in this sense, AOP information can be integrated together with ADME information in an IATA approach; (2) AOPs are not testing strategies. AOPs are mainly assemblies of knowledge. This structured and quality-controlled information can aid the interpretation of high-throughput testing or pathway-based data, in the context of relevant apical hazards; (3) AOPs are not mode of action (MoA) analyses. The MoA framework, as applied in human health risk assessment, represents a systematic description and analysis of the means through which a specific chemical elicits an adverse effect in an organism. AOPs, which are intended to be generalizable to any chemical acting on a particular MIE (chemical agnostic) can be applied to MoA analysis, but the terms are not synonymous; (4) AOPs are not computational models. Rather, they are intended to promote qualitative understanding of how an alteration in a KE_upstream_ impacts downstream KEs, and consequently provide information that may be represented in the form of one or more computational models (Wittwehr et al. 2017).

With regard to safety evaluation, AOPs may not comprehensively predict all toxicological outcomes. They do not solve all the challenges of in vitro to in vivo extrapolation. AOPs do not describe every detail of adverse and adaptive biology underlying an organism’s response to a stressor. They cannot account for every aspect of individual variability nor for every environmental or life-history variable that may affect a toxicological outcome in real-world settings. They are, in short, simply a means to help organize what we know about how biological perturbations can lead to apical adverse outcomes, and use that information to aid regulatory decision-making. In this context, this would mean that the present AOP is useful as guidance to judge the action of complex I inhibitors, but it cannot help to understand other neurotoxicants or pathways that cause motor degeneration. As for any complex tool, there is a main intended application, which one needs to be aware of. Use of the tool for other purposes may yield bad results, but this does not mean that the tool is bad for its original purpose.

The regulatory implications of this AOP have already been discussed in detail (Ockleford et al. 2017). Based on this AOP, it was concluded that if a chemical/pesticide triggers the MIE, it should be considered a risk factor for the development of PD.

## Outlook

The inclusion of an AOP into the AOP Wiki platform allows an integration of novel findings into an existing AOP. However, this flexibility requires the commitment not only from the original authors but also from other experts in the field, to continuously adjust the existing AOPs. Due to the laborious and time-consuming update process, without the “reward” in form of a publication authorship, a realistic approach to ensure a contemporary integration of novel observations into existing AOPs needs to involve a funding program to motivate comprehensive and qualified updates in defined intervals. For the motivation of scientists to participate in the development of new, or in the update of existing AOPs, promotion of the AOP concept to boost its reputation and awareness by the scientific community is another essential prerequisite for the successful establishment of AOPs as an integral tool in modern toxicology. As a consequence of this, regular revision of AOPs should be envisioned to keep them updated in line with the progress made in science, knowledge, and methodologies. Without such measures, there is a high risk that scientific development and regulatory needs advance while individual AOPs become outdated. As this may affect regulatory decision-making, it is a serious concern that needs to be considered in the future.

## References

[CR1] Abdelsalam RM, Safar MM (2015). Neuroprotective effects of vildagliptin in rat rotenone Parkinson’s disease model: role of RAGE-NFκB and Nrf2-antioxidant signaling pathways. J Neurochem.

[CR2] Adhihetty PJ, Beal MF (2008). Creatine and its potential therapeutic value for targeting cellular energy impairment in neurodegenerative diseases. Neuromol Med.

[CR3] Ahammadsahib KI, Hollingworth RM, McGovren JP, Hui YH, McLaughlin JL (1993). Mode of action of bullatacin: a potent antitumor and pesticidal annonaceous acetogenin. Life Sci.

[CR4] Alam M, Schmidt WJ (2002). Rotenone destroys dopaminergic neurons and induces parkinsonian symptoms in rats. Behav Brain Res.

[CR5] Alam M, Schmidt WJ (2004). L-DOPA reverses the hypokinetic behaviour and rigidity in rotenone-treated rats. Behav Brain Res.

[CR6] Albracht SP, Mariette A, de Jong P (1997). Bovine-heart NADH:ubiquinone oxidoreductase is a monomer with 8 Fe–S clusters and 2 FMN groups. Biochim Biophys Acta.

[CR7] Alexander GE, DeLong MR, Strick PL (1986). Parallel organization of functionally segregated circuits linking basal ganglia and cortex. Annu Rev Neurosci.

[CR8] Almeida A, Almeida J, Bolaños JP, Moncada S (2001). Different responses of astrocytes and neurons to nitric oxide: the role of glycolytically generated ATP in astrocyte protection. Proc Natl Acad Sci USA.

[CR9] Almeida A, Moncada S, Bolaños JP (2004). Nitric oxide switches on glycolysis through the AMP protein kinase and 6-phosphofructo-2-kinase pathway. Nat Cell Biol.

[CR10] Altarche-Xifro W, di Vicino U, Muñoz-Martin MI, Bortolozzi A, Bové J, Vila M, Cosma MP (2016). Functional rescue of dopaminergic neuron loss in Parkinson’s disease mice after transplantation of hematopoietic stem and progenitor cells. EBioMedicine.

[CR11] Alvarez-Fischer D, Guerreiro S, Hunot S, Saurini F, Marien M, Sokoloff P, Hirsch EC, Hartmann A, Michel PP (2008). Modelling Parkinson-like neurodegeneration via osmotic minipump delivery of MPTP and probenecid. J Neurochem.

[CR12] Ambrosi G, GhezziSepe S, Milanese C, Payan-Gomez C, Bombardieri CR, Armentero MT, Zangaglia R, Pacchetti C, Mastroberardino PG, Blandini F (2014). Bioenergetic and proteolytic defects in fibroblasts from patients with sporadic Parkinson’s disease. Biochim Biophys Acta.

[CR13] Appel SH (2009). CD4^+^ T cells mediate cytotoxicity in neurodegenerative diseases. J Clin Investig.

[CR14] Aschner M (1998). Immune and inflammatory responses in the CNS: modulation by astrocytes. Toxicol Lett.

[CR15] Aziz TZ, Peggs D, Sambrook MA, Crossman AR (1991). Lesion of the subthalamic nucleus for the alleviation of 1-methyl-4-phenyl-1,2,3,6-tetrahydropyridine (MPTP)-induced parkinsonism in the primate. Mov Disord.

[CR16] Bachschmid M, Schildknecht S, Ullrich V (2005). Redox regulation of vascular prostanoid synthesis by the nitric oxide-superoxide system. Biochem Biophys Res Commun.

[CR17] Bagyinszky E, Giau VV, Shim K, Suk K, An SSA, Kim S (2017). Role of inflammatory molecules in the Alzheimer’s disease progression and diagnosis. J Neurol Sci.

[CR18] Bain PG (2007). Parkinsonism and related disorders. Tremor Parkinsonism Relat Disord.

[CR19] Bal-Price A, Lein PJ, Keil KP, Sethi S, Shafer T, Barenys M, Fritsche E, Sachana M, Meek ME (2017). Developing and applying the adverse outcome pathway concept for understanding and predicting neurotoxicity. Neurotoxicology.

[CR20] Baltazar MT, Dinis-Oliveira RJ, de Lourdes Bastos M, Tsatsakis AM, Duarte JA, Carvalho F (2014). Pesticides exposure as etiological factors of Parkinson’s disease and other neurodegenerative diseases—a mechanistic approach. Toxicol Lett.

[CR21] Banati RB (2002). Visualising microglial activation in vivo. Glia.

[CR22] Banerjee R, Starkov AA, Beal MF, Thomas B (2009). Mitochondrial dysfunction in the limelight of Parkinson’s disease pathogenesis. Biochim Biophys Acta.

[CR23] Banks WA (2005). Blood-brain barrier transport of cytokines: a mechanism for neuropathology. Curr Pharm Des.

[CR24] Barbeito AG, Mesci P, Boillée S (2010). Motor neuron-immune interactions: the vicious circle of ALS. J Neural Transm (Vienna).

[CR25] Barnes CD (1983). The basal ganglia in extrapyramidal dysfunction. Brain Res Bull.

[CR26] Barrientos A, Moraes CT (1999). Titrating the effects of mitochondrial complex I impairment in the cell physiology. J Biol Chem.

[CR27] Bartels T, Choi JG, Selkoe DJ (2011). α-Synuclein occurs physiologically as a helically folded tetramer that resists aggregation. Nature.

[CR28] Baughman JM, Perocchi F, Girgis HS, Plovanich M, Belcher-Timme CA, Sancak Y, Bao XR, Strittmatter L, Goldberger O, Bogorad RL, Koteliansky V, Mootha VK (2011). Integrative genomics identifies MCU as an essential component of the mitochondrial calcium uniporter. Nature.

[CR29] Bauvy C, Meijer AJ, Codogno P (2009). Assaying of autophagic protein degradation. Methods Enzymol.

[CR30] Beal MF (2011). Neuroprotective effects of creatine. Amino Acids.

[CR31] Beal MF, Matthews RT, Tieleman A, Shults CW (1998). Coenzyme Q10 attenuates the 1-methyl-4-phenyl-1,2,3,tetrahydropyridine (MPTP) induced loss of striatal dopamine and dopaminergic axons in aged mice. Brain Res.

[CR32] Bédard PJ, Di Paolo T, Falardeau P, Boucher R (1986). Chronic treatment with L-DOPA, but not bromocriptine induces dyskinesia in MPTP-parkinsonian monkeys. Correlation with [^3^H]spiperone binding. Brain Res.

[CR33] Bellucci A, Zaltieri M, Navarria L, Grigoletto J, Missale C, Spano P (2012). From α-synuclein to synaptic dysfunctions: new insights into the pathophysiology of Parkinson’s disease. Brain Res.

[CR34] Benamer HT, Patterson J, Wyper DJ, Hadley DM, Macphee GJ, Grosset DG (2000). Correlation of Parkinson’s disease severity and duration with ^123^I-FP-CIT SPECT striatal uptake. Mov Disord.

[CR35] Bence NF, Sampat RM, Kopito RR (2001). Impairment of the ubiquitin-proteasome system by protein aggregation. Science.

[CR36] Ben-Hur T, Idelson M, Khaner H, Pera M, Reinhartz E, Itzik A, Reubinoff BE (2004). Transplantation of human embryonic stem cell-derived neural progenitors improves behavioral deficit in Parkinsonian rats. Stem Cells.

[CR37] Bentea E, Van der Perren A, Van Liefferinge J, El Arfani A, Albertini G, Demuyser T, Merckx E, Michotte Y, Smolders I, Baekelandt V, Massie A (2015). Nigral proteasome inhibition in mice leads to motor and non-motor deficits and increased expression of Ser129 phosphorylated α-synuclein. Front Behav Neurosci.

[CR38] Béraud D, Hathaway HA, Trecki J, Chasovskikh S, Johnson DA, Johnson JA, Federoff HJ, Shimoji M, Mhyre TR, Maguire-Zeiss KA (2013). Microglial activation and antioxidant responses induced by the Parkinson’s disease protein α-synuclein. J Neuroimmune Pharmacol.

[CR39] Berger I, Hershkovitz E, Shaag A, Edvardson S, Saada A, Elpeleg O (2008). Mitochondrial complex I deficiency caused by a deleterious NDUFA11 mutation. Ann Neurol.

[CR40] Bergman H, Wichmann T, DeLong MR (1990). Reversal of experimental parkinsonism by lesions of the subthalamic nucleus. Science.

[CR41] Bermejo A, Figadere B, Zafra-Polo MC, Barrachina I, Estornell E, Cortes D (2005). Acetogenins from Annonaceae: recent progress in isolation, synthesis and mechanisms of action. Nat Prod Rep.

[CR42] Bernheimer H, Birkmayer W, Hornykiewicz O, Jellinger K, Seitelberger F (1973). Brain dopamine and the syndromes of Parkinson and Huntington. Clinical, morphological and neurochemical correlations. J Neurol Sci.

[CR43] Betarbet R, Sherer TB, MacKenzie G, Garcia-Osuna M, Panov AV, Greenamyre JT (2000). Chronic systemic pesticide exposure reproduces features of Parkinson’s disease. Nat Neurosci.

[CR44] Betarbet R, Sherer TB, Greenamyre JT (2005). Ubiquitin-proteasome system and Parkinson’s diseases. Exp Neurol.

[CR45] Betarbet R, Canet-Aviles RM, Sherer TB, Mastroberardino PG, McLendon C, Kim JH, Lund S, Na HM, Taylor G, Bence NF, Kopito R, Seo BB, Yagi T, Yagi A, Klinefelter G, Cookson MR, Greenamyre JT (2006). Intersecting pathways to neurodegeneration in Parkinson’s disease: effects of the pesticide rotenone on DJ-1, alpha-synuclein, and the ubiquitin-proteasome system. Neurobiol Dis.

[CR46] Bezard E, Dovero S, Prunier C, Ravenscroft P, Chalon S, Guilloteau D, Crossman AR, Bioulac B, Brotchie JM, Gross CE (2001). Relationship between the appearance of symptoms and the level of nigrostriatal degeneration in a progressive 1-methyl-4-phenyl-1,2,3,6-tetrahydropyridine-lesioned macaque model of Parkinson’s disease. J Neurosci.

[CR47] Bi J, Wang XB, Chen L, Hao S, An LJ, Jiang B, Guo L (2008). Catalpol protects mesencephalic neurons against MPTP induced neurotoxicity via attenuation of mitochondrial dysfunction and MAO-B activity. Toxicol In Vitro.

[CR48] Blandini F, Nappi G, Tassorelli C, Martignoni E (2000). Functional changes of the basal ganglia circuitry in Parkinson’s disease. Prog Neurobiol.

[CR49] Blank T, Prinz M (2013). Microglia as modulators of cognition and neuropsychiatric disorders. Glia.

[CR50] Blasko I, Stampfer-Kountchev M, Robatscher P, Veerhuis R, Eikelenboom P, Grubeck-Loebenstein B (2004). How chronic inflammation can affect the brain and support the development of Alzheimer’s disease in old age: the role of microglia and astrocytes. Aging Cell.

[CR51] Blesa J, Pifl C, Sánchez-González MA, Juri C, García-Cabezas MA, Adánez R, Iglesias E, Collantes M, Peñuelas I, Sánchez-Hernández JJ, Rodríguez-Oroz MC, Avendaño C, Hornykiewicz O, Cavada C, Obeso JA (2012). The nigrostriatal system in the presymptomatic and symptomatic stages in the MPTP monkey model: a PET, histological and biochemical study. Neurobiol Dis.

[CR52] Boka G, Anglade P, Wallach D, Javoy-Agid F, Agid Y, Hirsch EC (1994). Immunocytochemical analysis of tumor necrosis factor and its receptors in Parkinson’s disease. Neurosci Lett.

[CR53] Bolam JP, Pissadaki EK (2012). Living on the edge with too many mouths to feed: why dopamine neurons die. Mov Disord.

[CR54] Bolam JP, Hanley JJ, Booth PA, Bevan MD (2000). Synaptic organisation of the basal ganglia. J Anat.

[CR55] Borland MK, Trimmer PA, Rubinstein JD, Keeney PM, Mohanakumar K, Liu L, Bennett JP (2008). Chronic, low-dose rotenone reproduces Lewy neurites found in early stages of Parkinson’s disease, reduces mitochondrial movement and slowly kills differentiated SH-SY5Y neural cells. Mol Neurodegener.

[CR56] Borutaite V, Budriunaite A, Brown GC (2000). Reversal of nitric oxide-, peroxynitrite- and S-nitrosothiol-induced inhibition of mitochondrial respiration or complex I activity by light and thiols. Biochim Biophys Acta.

[CR57] Bose A, Beal MF (2016). Mitochondrial dysfunction in Parkinson’s disease. J Neurochem.

[CR58] Braak H, Ghebremedhin E, Rüb U, Bratzke H, Del Tredici K (2004). Stages in the development of Parkinson’s disease-related pathology. Cell Tissue Res.

[CR59] Brand MD (2010). The sites and topology of mitochondrial superoxide production. Exp Gerontol.

[CR60] Brandt U (1997). Proton-translocation by membrane-bound NADH:ubiquinone-oxidoreductase (complex I) through redox-gated ligand conduction. Biochim Biophys Acta.

[CR61] Braun RJ (2012). Mitochondrion-mediated cell death: dissecting yeast apoptosis for a better understanding of neurodegeneration. Front Oncol.

[CR62] Breckenridge CB, Berry C, Chang ET, Sielken RL, Mandel JS (2016). Association between Parkinson’s disease and cigarette smoking, rural living, well-water consumption, farming and pesticide use: systematic review and meta-analysis. PLoS One.

[CR63] Brichta L, Greengard P (2014). Molecular determinants of selective dopaminergic vulnerability in Parkinson’s disease: an update. Front Neuroanat.

[CR64] Brini M, Calì T, Ottolini D, Carafoli E (2014). Neuronal calcium signaling: function and dysfunction. Cell Mol Life Sci.

[CR65] Brinkley BR, Barham SS, Barranco SC, Fuller GM (1974). Rotenone inhibition of spindle microtubule assembly in mammalian cells. Exp Cell Res.

[CR66] Brochard V, Combadière B, Prigent A, Laouar Y, Perrin A, Beray-Berthat V, Bonduelle O, Alvarez-Fischer D, Callebert J, Launay JM, Duyckaerts C, Flavell RA, Hirsch EC, Hunot S (2009). Infiltration of CD4^+^ lymphocytes into the brain contributes to neurodegeneration in a mouse model of Parkinson disease. J Clin Investig.

[CR67] Brown GC, Bal-Price A (2003). Inflammatory neurodegeneration mediated by nitric oxide, glutamate, and mitochondria. Mol Neurobiol.

[CR68] Brownell AL, Jenkins BG, Elmaleh DR, Deacon TW, Spealman RD, Isacson O (1998). Combined PET/MRS brain studies show dynamic and long-term physiological changes in a primate model of Parkinson disease. Nat Med.

[CR69] Burwell LS, Nadtochiy SM, Tompkins AJ, Young S, Brookes PS (2006). Direct evidence for S-nitrosation of mitochondrial complex I. Biochem J.

[CR70] Butterfield DA, Kanski J (2001). Brain protein oxidation in age-related neurodegenerative disorders that are associated with aggregated proteins. Mech Ageing Dev.

[CR71] Calì T, Ottolini D, Brini M (2014). Calcium signaling in Parkinson’s disease. Cell Tissue Res.

[CR72] Cannon JR, Tapias V, Na HM, Honick AS, Drolet RE, Greenamyre JT (2009). A highly reproducible rotenone model of Parkinson’s disease. Neurobiol Dis.

[CR73] Castaño A, Herrera AJ, Cano J, Machado A (2002). The degenerative effect of a single intranigral injection of LPS on the dopaminergic system is prevented by dexamethasone, and not mimicked by rh-TNF-alpha, IL-1beta and IFN-gamma. J Neurochem.

[CR74] Chan P, DeLanney LE, Irwin I, Langston JW, Di Monte D (1991). Rapid ATP loss caused by 1-methyl-4-phenyl-1,2,3,6-tetrahydropyridine in mouse brain. J Neurochem.

[CR75] Chan CS, Guzman JN, Ilijic E, Mercer JN, Rick C, Tkatch T, Meredith GE, Surmeier DJ (2007). ‘Rejuvenation’ protects neurons in mouse models of Parkinson’s disease. Nature.

[CR76] Chang DT, Honick AS, Reynolds IJ (2006). Mitochondrial trafficking to synapses in cultured primary cortical neurons. J Neurosci.

[CR77] Chao Y, Wong SC, Tan EK (2014). Evidence of inflammatory system involvement in Parkinson’s disease. Biomed Res Int.

[CR78] Chapman GA, Moores K, Harrison D, Campbell CA, Stewart BR, Strijbos PJ (2000). Fractalkine cleavage from neuronal membranes represents an acute event in the inflammatory response to excitotoxic brain damage. J Neurosci.

[CR79] Charan RA, Johnson BN, Zaganelli S, Nardozzi JD, LaVoie MJ (2014). Inhibition of apoptotic Bax translocation to the mitochondria is a central function of parkin. Cell Death Dis.

[CR80] Chartier-Harlin MC, Kachergus J, Roumier C, Mouroux V, Douay X, Lincoln S, Levecque C, Larvor L, Andrieux J, Hulihan M, Waucquier N, Defebvre L, Amouyel P, Farrer M, Destée A (2004). Alpha-synuclein locus duplication as a cause of familial Parkinson’s disease. Lancet.

[CR81] Chen H, Zhang SM, Hernán MA, Schwarzschild MA, Willett WC, Colditz GA, Speizer FE, Ascherio A (2003). Nonsteroidal anti-inflammatory drugs and the risk of Parkinson disease. Arch Neurol.

[CR82] Chen H, Jacobs E, Schwarzschild MA, McCullough ML, Calle EE, Thun MJ, Ascherio A (2005). Nonsteroidal antiinflammatory drug use and the risk for Parkinson’s disease. Ann Neurol.

[CR83] Chen L, Jin J, Davis J, Zhou Y, Wang Y, Liu J, Lockhart PJ, Zhang J (2007). Oligomeric alpha-synuclein inhibits tubulin polymerization. Biochem Biophys Res Commun.

[CR84] Chen Y, Zhang DQ, Liao Z, Wang B, Gong S, Wang C, Zhang MZ, Wang GH, Cai H, Liao FF, Xu JP (2015). Anti-oxidant polydatin (piceid) protects against substantia nigral motor degeneration in multiple rodent models of Parkinson’s disease. Mol Neurodegener.

[CR85] Chinta SJ, Andersen JK (2006). Reversible inhibition of mitochondrial complex I activity following chronic dopaminergic glutathione depletion in vitro: implications for Parkinson’s disease. Free Radic Biol Med.

[CR86] Chinta SJ, Mallajosyula JK, Rane A, Andersen JK (2010). Mitochondrial α-synuclein accumulation impairs complex I function in dopaminergic neurons and results in increased mitophagy in vivo. Neurosci Lett.

[CR87] Choi WS, Kruse SE, Palmiter RD, Xia Z (2008). Mitochondrial complex I inhibition is not required for dopminergic neuron death induced by rotenone, MPP^+^, or paraquat. Proc Natl Acad Sci USA.

[CR88] Choi BS, Kim H, Lee HJ, Sapkota K, Park SE, Kim S, Kim SJ (2014). Celastrol from ‘Thunder God Vine’ protects SH-SY5Y cells through the preservation of mitochondrial function and inhibition of p38 MAPK in a rotenone model of Parkinson’s disease. Neurochem Res.

[CR89] Chou AP, Li S, Fitzmaurice AG, Bronstein JM (2010). Mechanisms of rotenone-induced proteasome inhibition. Neurotoxicology.

[CR90] Chu CT, Ji J, Dagda RK, Jiang JF, Tyurina YY, Kapralov AA, Tyurin VA, Yanamala N, Shrivastava IH, Mohammadyani D, Qiang Wang KZ, Zhu J, Klein-Seetharaman J, Balasubramanian K, Amoscato AA, Borisenko G, Huang Z, Gusdon AM, Cheikhi A, Steer EK, Wang R, Baty C, Watkins S, Bahar I, Bayır H, Kagan VE (2013). Cardiolipin externalization to the outer mitochondrial membrane acts as an elimination signal for mitophagy in neuronal cells. Nat Cell Biol.

[CR91] Ciapaite J, Van Eikenhorst G, Bakker SJ, Diamant M, Heine RJ, Wagner MJ, Westerhoff HV, Krab K (2005). Modular kinetic analysis of the adenine nucleotide translocator-mediated effects of palmitoyl-CoA on the oxidative phosphorylation in isolated rat liver mitochondria. Diabetes.

[CR92] Ciechanover A (1998). The ubiquitin-proteasome pathway: on protein death and cell life. EMBO J.

[CR93] Ciechanover A, Brundin P (2003). The ubiquitin proteasome system in neurodegenerative diseases: sometimes the chicken, sometimes the egg. Neuron.

[CR94] Clarke CE, Sambrook MA, Mitchell IJ, Crossman AR (1987). Levodopa-induced dyskinesia and response fluctuations in primates rendered parkinsonian with 1-methyl-4-phenyl-1,2,3,6-tetrahydropyridine (MPTP). J Neurol Sci.

[CR95] Claycomb KI, Johnson KM, Winokur PN, Sacino AV, Crocker SJ (2013). Astrocyte regulation of CNS inflammation and remyelination. Brain Sci.

[CR96] Cleeter MW, Cooper JM, Schapira AH (1992). Irreversible inhibition of mitochondrial complex I by 1-methyl-4-phenylpyridinium: evidence for free radical involvement. J Neurochem.

[CR97] Correia SC, Santos RX, Perry G, Zhu X, Moreira PI, Smith MA (2012). Mitochondrial importance in Alzheimer’s, Huntington’s and Parkinson’s diseases. Adv Exp Med Biol.

[CR98] Crawley JN (1999). Behavioral phenotyping of transgenic and knockout mice: experimental design and evaluation of general health, sensory functions, motor abilities, and specific behavioral tests. Brain Res.

[CR99] Cuervo AM (2004). Autophagy: many paths to the same end. Mol Cell Biochem.

[CR100] Cunha MP, Martín-de-Saavedra MD, Romero A, Egea J, Ludka FK, Tasca CI, Farina M, Rodrigues AL, López MG (2014) Both creatine and its product phosphocreatine reduce oxidative stress and afford neuroprotection in an in vitro Parkinson’s model. ASN Neuro 6(6) 10.1177/1759091414554945 PMC4357608 25424428

[CR101] Dahm CC, Moore K, Murphy MP (2006). Persistent S-nitrosation of complex I and other mitochondrial membrane proteins by S-nitrosothiols but not nitric oxide or peroxynitrite: implications for the interaction of nitric oxide with mitochondria. J Biol Chem.

[CR102] Daiber A, Schildknecht S, Müller J, Kamuf J, Bachschmid MM, Ullrich V (2009). Chemical model systems for cellular nitros(yl)ation reactions. Free Radic Biol Med.

[CR103] Dauer W, Przedborski S (2003). Parkinson’s disease: mechanisms and models. Neuron.

[CR104] Dauer W, Kholodilov N, Vila M, Trillat AC, Goodchild R, Larsen KE, Staal R, Tieu K, Schmitz Y, Yuan CA, Rocha M, Jackson-Lewis V, Hersch S, Sulzer D, Przedborski S, Burke R, Hen R (2002). Resistance of alpha -synuclein null mice to the parkinsonian neurotoxin MPTP. Proc Natl Acad Sci USA.

[CR105] Davalos D, Grutzendler J, Yang G, Kim JV, Zuo Y, Jung S, Littman DR, Dustin ML, Gan WB (2005). ATP mediates rapid microglial response to local brain injury in vivo. Nat Neurosci.

[CR106] Davies KJ (2001). Degradation of oxidized proteins by the 20S proteasome. Biochimie.

[CR107] De Stefani D, Raffaello A, Teardo E, Szabò I, Rizzuto R (2011). A forty-kilodalton protein of the inner membrane is the mitochondrial calcium uniporter. Nature.

[CR108] Decressac M, Björklund A (2013). TFEB: Pathogenic role and therapeutic target in Parkinson disease. Autophagy.

[CR109] Decressac M, Kadkhodaei B, Mattsson B, Laguna A, Perlmann T, Björklund A (2012). α-Synuclein-induced down-regulation of Nurr1 disrupts GDNF signaling in nigral dopamine neurons. Sci Transl Med.

[CR110] Decressac M, Mattsson B, Weikop P, Lundblad M, Jakobsson J, Björklund A (2013). TFEB-mediated autophagy rescues midbrain dopamine neurons from α-synuclein toxicity. Proc Natl Acad Sci USA.

[CR111] Degli Esposti M (1998). Inhibitors of NADH-ubiquinone reductase: an overview. Biochim Biophys Acta.

[CR112] Degli Esposti M, Ghelli A (1994). The mechanism of proton and electron transport in mitochondrial complex I. Biochim Biophys Acta.

[CR113] Degli Esposti M, Ghelli A, Crimi M, Estornell E, Fato R, Lenaz G (1993). Complex I and complex III of mitochondria have common inhibitors acting as ubiquinone antagonists. Biochem Biophys Res Commun.

[CR114] Degli Esposti M, Ghelli A, Ratta M, Cortes D, Estornell E (1994). Natural substances (acetogenins) from the family Annonaceae are powerful inhibitors of mitochondrial NADH dehydrogenase (Complex I). Biochem J.

[CR115] Degli Esposti M, Ngo A, McMullen GL, Ghelli A, Sparla F, Benelli B, Ratta M, Linnane AW (1996). The specificity of mitochondrial complex I for ubiquinones. Biochem J.

[CR116] Dehay B, Bové J, Rodríguez-Muela N, Perier C, Recasens A, Boya P, Vila M (2010). Pathogenic lysosomal depletion in Parkinson’s disease. J Neurosci.

[CR117] Demasi M, Shringarpure R, Davies KJ (2001). Glutathiolation of the proteasome is enhanced by proteolytic inhibitors. Arch Biochem Biophys.

[CR118] Demasi M, Silva GM, Netto LE (2003). 20 S proteasome from *Saccharomyces cerevisiae* is responsive to redox modifications and is S-glutathionylated. J Biol Chem.

[CR119] Devi L, Raghavendran V, Prabhu BM, Avadhani NG, Anandatheerthavarada HK (2008). Mitochondrial import and accumulation of alpha-synuclein impair complex I in human dopaminergic neuronal cultures and Parkinson disease brain. J Biol Chem.

[CR120] Ding Q, Dimayuga E, Martin S, Bruce-Keller AJ, Nukala V, Cuervo AM, Keller JN (2003). Characterization of chronic low-level proteasome inhibition on neural homeostasis. J Neurochem.

[CR121] Dong Y, Benveniste EN (2001). Immune function of astrocytes. Glia.

[CR122] Drolet RE, Behrouz B, Lookingland KJ, Goudreau JL (2004). Mice lacking alpha-synuclein have an attenuated loss of striatal dopamine following prolonged chronic MPTP administration. Neurotoxicology.

[CR123] Du Y, Ma Z, Lin S, Dodel RC, Gao F, Bales KR, Triarhou LC, Chernet E, Perry KW, Nelson DL, Luecke S, Phebus LA, Bymaster FP, Paul SM (2001). Minocycline prevents nigrostriatal dopaminergic neurodegeneration in the MPTP model of Parkinson’s disease. Proc Natl Acad Sci USA.

[CR124] Dukes AA, Bai Q, Van Laar VS, Zhou Y, Ilin V, David CN, Agim ZS, Bonkowsky JL, Cannon JR, Watkins SC, Croix CM, Burton EA, Berman SB (2016). Live imaging of mitochondrial dynamics in CNS dopaminergic neurons in vivo demonstrates early reversal of mitochondrial transport following MPP(+) exposure. Neurobiol Dis.

[CR125] Dunning CJ, McKenzie M, Sugiana C, Lazarou M, Silke J, Connelly A, Fletcher JM, Kirby DM, Thorburn DR, Ryan MT (2007). Human CIA30 is involved in the early assembly of mitochondrial complex I and mutations in its gene cause disease. EMBO J.

[CR126] Earle KM (1968). Studies on Parkinson’s disease including X-ray fluorescent spectroscopy of formalin fixed brain tissue. J Neuropathol Exp Neurol.

[CR127] Ebrahimi-Fakhari D, Wahlster L (2013). Restoring impaired protein metabolism in Parkinson’s disease—TFEB-mediated autophagy as a novel therapeutic target. Mov Disord.

[CR128] Efremova L, Schildknecht S, Adam M, Pape R, Gutbier S, Hanf B, Bürkle A, Leist M (2015). Prevention of the degeneration of human dopaminergic neurons in an astrocyte co-culture system allowing endogenous drug metabolism. Br J Pharmacol.

[CR129] Ehringer H, Hornykiewicz OHRINGERH, HORNYKIEWICZ O (1960). Distribution of noradrenaline and dopamine (3-hydroxytyramine) in the human brain and their behavior in diseases of the extrapyramidal system. Klin Wochenschr.

[CR130] Ekstrand MI, Terzioglu M, Galter D, Zhu S, Hofstetter C, Lindqvist E, Thams S, Bergstrand A, Hansson FS, Trifunovic A, Hoffer B, Cullheim S, Mohammed AH, Olson L, Larsson NG (2007). Progressive parkinsonism in mice with respiratory-chain-deficient dopamine neurons. Proc Natl Acad Sci USA.

[CR131] Emmanouilidou E, Melachroinou K, Roumeliotis T, Garbis SD, Ntzouni M, Margaritis LH, Stefanis L, Vekrellis K (2010). Cell-produced alpha-synuclein is secreted in a calcium-dependent manner by exosomes and impacts neuronal survival. J Neurosci.

[CR132] Eng LF, Ghirnikar RS, Lee YL (2000). Glial fibrillary acidic protein: GFAP-thirty-one years (1969–2000). Neurochem Res.

[CR133] Ernster L, Lee C (1967). Energy-linked reduction of nAD^+^ by succinate. Methods Enzym.

[CR134] Esposito A, Dohm CP, Kermer P, Bähr M, Wouters FS (2007). alpha-Synuclein and its disease-related mutants interact differentially with the microtubule protein tau and associate with the actin cytoskeleton. Neurobiol Dis.

[CR135] Etminan M, Carleton BC, Samii A (2008). Non-steroidal anti-inflammatory drug use and the risk of Parkinson disease: a retrospective cohort study. J Clin Neurosci.

[CR136] Fabre E, Monserrat J, Herrero A, Barja G, Leret ML (1999). Effect of MPTP on brain mitochondrial H2O2 and ATP production and on dopamine and DOPAC in the striatum. J Physiol Biochem.

[CR137] Falsig J, Latta M, Leist M (2004). Defined inflammatory states in astrocyte cultures: correlation with susceptibility towards CD95-driven apoptosis. J Neurochem.

[CR138] Falsig J, Pörzgen P, Lund S, Schrattenholz A, Leist M (2006). The inflammatory transcriptome of reactive murine astrocytes and implications for their innate immune function. J Neurochem.

[CR139] Falsig J, van Beek J, Hermann C, Leist M (2008). Molecular basis for detection of invading pathogens in the brain. J Neurosci Res.

[CR140] Farina C, Aloisi F, Meinl E (2007). Astrocytes are active players in cerebral innate immunity. Trends Immunol.

[CR141] Farout L, Mary J, Vinh J, Szweda LI, Friguet B (2006). Inactivation of the proteasome by 4-hydroxy-2-nonenal is site specific and dependant on 20S proteasome subtypes. Arch Biochem Biophys.

[CR142] Faust K, Gehrke S, Yang Y, Yang L, Beal MF, Lu B (2009). Neuroprotective effects of compounds with antioxidant and anti-inflammatory properties in a Drosophila model of Parkinson’s disease. BMC Neurosci.

[CR143] Fellner L, Irschick R, Schanda K, Reindl M, Klimaschewski L, Poewe W, Wenning GK, Stefanova N (2013). Toll-like receptor 4 is required for α-synuclein dependent activation of microglia and astroglia. Glia.

[CR144] Ferger B, Leng A, Mura A, Hengerer B, Feldon J (2004). Genetic ablation of tumor necrosis factor-alpha (TNF-alpha) and pharmacological inhibition of TNF-synthesis attenuates MPTP toxicity in mouse striatum. J Neurochem.

[CR145] Fernandez-Moreira D, Ugalde C, Smeets R, Rodenburg RJ, Lopez-Laso E, Ruiz-Falco ML, Briones P, Martin MA, Smeitink JA, Arenas J (2007). X-linked NDUFA1 gene mutations associated with mitochondrial encephalomyopathy. Ann Neurol.

[CR146] Fetissov SO, Marsais F (1999). Combination of immunohistochemical and in situ hybridization methods to reveal tyrosine hydroxylase and oxytocin and vasopressin mRNAs in magnocellular neurons of obese Zucker rats. Brain Res Brain Res Protoc.

[CR147] Filion M, Tremblay L (1991). Abnormal spontaneous activity of globus pallidus neurons in monkeys with MPTP-induced parkinsonism. Brain Res.

[CR148] Filomeni G, Graziani I, De Zio D, Dini L, Centonze D, Rotilio G, Ciriolo MR (2012). Neuroprotection of kaempferol by autophagy in models of rotenone-mediated acute toxicity: possible implications for Parkinson’s disease. Neurobiol Aging.

[CR149] Finley D (2009). Recognition and processing of ubiquitin-protein conjugates by the proteasome. Annu Rev Biochem.

[CR150] Fleming SM, Zhu C, Fernagut PO, Mehta A, DiCarlo CD, Seaman RL, Chesselet MF (2004). Behavioral and immunohistochemical effects of chronic intravenous and subcutaneous infusions of varying doses of rotenone. Exp Neurol.

[CR151] Follett J, Darlow B, Wong MB, Goodwin J, Pountney DL (2013). Potassium depolarization and raised calcium induces α-synuclein aggregates. Neurotox Res.

[CR152] Fornai F, Lenzi P, Gesi M, Ferrucci M, Lazzeri G, Busceti CL, Ruffoli R, Soldani P, Ruggieri S, Alessandri MG, Paparelli A (2003). Fine structure and biochemical mechanisms underlying nigrostriatal inclusions and cell death after proteasome inhibition. J Neurosci.

[CR153] Fornai F, Schlüter OM, Lenzi P, Gesi M, Ruffoli R, Ferrucci M, Lazzeri G, Busceti CL, Pontarelli F, Battaglia G, Pellegrini A, Nicoletti F, Ruggieri S, Paparelli A, Südhof TC (2005). Parkinson-like syndrome induced by continuous MPTP infusion: convergent roles of the ubiquitin-proteasome system and alpha-synuclein. Proc Natl Acad Sci USA.

[CR154] Forno LS, Langston JW, DeLanney LE, Irwin I, Ricaurte GA (1986). Locus ceruleus lesions and eosinophilic inclusions in MPTP-treated monkeys. Ann Neurol.

[CR155] Fossati S, Chiarugi A (2007). Relevance of high-mobility group protein box 1 to neurodegeneration. Int Rev Neurobiol.

[CR156] Frank-Cannon TC, Tran T, Ruhn KA, Martinez TN, Hong J, Marvin M, Hartley M, Treviño I, O’Brien DE, Casey B, Goldberg MS, Tansey MG (2008). Parkin deficiency increases vulnerability to inflammation-related nigral degeneration. J Neurosci.

[CR157] Friedrich T, van Heek P, Leif H, Ohnishi T, Forche E, Kunze B, Jansen R, Trowitzsch-Kienast W, Höfle G, Reichenbach H (1994). Two binding sites of inhibitors in NADH: ubiquinone oxidoreductase (complex I). Relationship of one site with the ubiquinone-binding site of bacterial glucose:ubiquinone oxidoreductase. Eur J Biochem.

[CR158] Fujita S, Kiguchi M, Lee J, Terakado M, Suga K, Hatanaka H, Koshikawa N (2008). 5-HT(1A) and 5-HT(1B) receptors in the ventrolateral striatum differentially modulate apomorphine-induced jaw movements in rats. J Oral Sci.

[CR159] Gainetdinov RR, Jones SR, Caron MG (1999). Functional hyperdopaminergia in dopamine transporter knock-out mice. Biol Psychiatry.

[CR160] Galkin A, Brandt U (2005). Superoxide radical formation by pure complex I (NADH:ubiquinone oxidoreductase) from Yarrowia lipolytica. J Biol Chem.

[CR161] Galpern WR, Cudkowicz ME (2007). Coenzyme Q treatment of neurodegenerative diseases of aging. Mitochondrion.

[CR162] Gandhi S, Wood-Kaczmar A, Yao Z, Plun-Favreau H, Deas E, Klupsch K, Downward J, Latchman DS, Tabrizi SJ, Wood NW, Duchen MR, Abramov AY (2009). PINK1-associated Parkinson’s disease is caused by neuronal vulnerability to calcium-induced cell death. Mol Cell.

[CR163] Genova ML, Ventura B, Giuliano G, Bovina C, Formiggini G, Parenti Castelli G, Lenaz G (2001). The site of production of superoxide radical in mitochondrial Complex I is not a bound ubisemiquinone but presumably iron–sulfur cluster N_2_. FEBS Lett.

[CR164] Gerfen CR, Engber TM, Mahan LC, Susel Z, Chase TN, Monsma FJ, Sibley DR (1990). D1 and D2 dopamine receptor-regulated gene expression of striatonigral and striatopallidal neurons. Science.

[CR165] Gibrat C, Saint-Pierre M, Bousquet M, Lévesque D, Rouillard C, Cicchetti F (2009). Differences between subacute and chronic MPTP mice models: investigation of dopaminergic neuronal degeneration and alpha-synuclein inclusions. J Neurochem.

[CR166] Gilmour TP, Lieu CA, Nolt MJ, Piallat B, Deogaonkar M, Subramanian T (2011). The effects of chronic levodopa treatments on the neuronal firing properties of the subthalamic nucleus and substantia nigra reticulata in hemiparkinsonian rhesus monkeys. Exp Neurol.

[CR167] Giordano S, Lee J, Darley-Usmar VM, Zhang J (2012). Distinct effects of rotenone, 1-methyl-4-phenylpyridinium and 6-hydroxydopamine on cellular bioenergetics and cell death. PLoS One.

[CR168] Giordano S, Darley-Usmar V, Zhang J (2013). Autophagy as an essential cellular antioxidant pathway in neurodegenerative disease. Redox Biol.

[CR169] Giordano S, Dodson M, Ravi S, Redmann M, Ouyang X, Darley Usmar VM, Zhang J (2014). Bioenergetic adaptation in response to autophagy regulators during rotenone exposure. J Neurochem.

[CR170] Gluck MR, Krueger MJ, Ramsay RR, Sablin SO, Singer TP, Nicklas WJ (1994). Characterization of the inhibitory mechanism of 1-methyl-4-phenylpyridinium and 4-phenylpyridine analogs in inner membrane preparations. J Biol Chem.

[CR171] Goldberg AL (2003). Protein degradation and protection against misfolded or damaged proteins. Nature.

[CR172] Goodwin J, Nath S, Engelborghs Y, Pountney DL (2013). Raised calcium and oxidative stress cooperatively promote alpha-synuclein aggregate formation. Neurochem Int.

[CR173] Graeber MB, Streit WJ (1990). Microglia: immune network in the CNS. Brain Pathol.

[CR174] Graier WF, Frieden M, Malli R (2007). Mitochondria and Ca(2+) signaling: old guests, new functions. Pflugers Arch.

[CR175] Greenamyre JT, Higgins DS, Eller RV (1992). Quantitative autoradiography of dihydrorotenone binding to complex I of the electron transport chain. J Neurochem.

[CR176] Greenamyre JT, Sherer TB, Betarbet R, Panov AV (2001). Complex I and Parkinson’s disease. IUBMB Life.

[CR177] Griffin WS, Sheng JG, Royston MC, Gentleman SM, McKenzie JE, Graham DI, Roberts GW, Mrak RE (1998). Glial-neuronal interactions in Alzheimer’s disease: the potential role of a ‘cytokine cycle’ in disease progression. Brain Pathol.

[CR178] Grivennikova VG, Vinogradov AD (2006). Generation of superoxide by the mitochondrial Complex I. Biochim Biophys Acta.

[CR179] Grivennikova VG, Maklashina EO, Gavrikova EV, Vinogradov AD (1997). Interaction of the mitochondrial NADH-ubiquinone reductase with rotenone as related to the enzyme active/inactive transition. Biochim Biophys Acta.

[CR180] Grosch J, Winkler J, Kohl Z (2016). Early degeneration of both dopaminergic and serotonergic axons—a common mechanism in Parkinson’s disease. Front Cell Neurosci.

[CR181] Guzman JN, Sánchez-Padilla J, Chan CS, Surmeier DJ (2009). Robust pacemaking in substantia nigra dopaminergic neurons. J Neurosci.

[CR182] Hafner RP, Brown GC, Brand MD (1990). Analysis of the control of respiration rate, phosphorylation rate, proton leak rate and protonmotive force in isolated mitochondria using the ‘top-down’ approach of metabolic control theory. Eur J Biochem.

[CR183] Hajieva P, Mocko JB, Moosmann B, Behl C (2009). Novel imine antioxidants at low nanomolar concentrations protect dopaminergic cells from oxidative neurotoxicity. J Neurochem.

[CR184] Han Y, Gao P, Qiu S, Zhang L, Yang L, Zuo J, Zhong C, Zhu S, Liu W (2016). MTERF2 contributes to MPP(+)-induced mitochondrial dysfunction and cell damage. Biochem Biophys Res Commun.

[CR185] Hantraye P, Varastet M, Peschanski M, Riche D, Cesaro P, Willer JC, Maziere M (1993). Stable parkinsonian syndrome and uneven loss of striatal dopamine fibres following chronic MPTP administration in baboons. Neuroscience.

[CR186] Hasegawa E, Takeshige K, Oishi T, Murai Y, Minakami S (1990). 1-Methyl-4-phenylpyridinium (MPP^+^) induces NADH-dependent superoxide formation and enhances NADH-dependent lipid peroxidation in bovine heart submitochondrial particles. Biochem Biophys Res Commun.

[CR187] Haynes SE, Hollopeter G, Yang G, Kurpius D, Dailey ME, Gan WB, Julius D (2006). The P2Y12 receptor regulates microglial activation by extracellular nucleotides. Nat Neurosci.

[CR188] He Q, Yu W, Wu J, Chen C, Lou Z, Zhang Q, Zhao J, Wang J, Xiao B (2013). Intranasal LPS-mediated Parkinson’s model challenges the pathogenesis of nasal cavity and environmental toxins. PLoS One.

[CR189] Heimer G, Bar-Gad I, Goldberg JA, Bergman H (2002). Dopamine replacement therapy reverses abnormal synchronization of pallidal neurons in the 1-methyl-4-phenyl-1,2,3,6-tetrahydropyridine primate model of parkinsonism. J Neurosci.

[CR190] Heimer G, Rivlin M, Israel Z, Bergman H (2006) Synchronizing activity of basal ganglia and pathophysiology of Parkinson’s disease. J Neural Transm Suppl. (70):17–20 10.1007/978-3-211-45295-0_4 17017503

[CR191] Heo JY, Park JH, Kim SJ, Seo KS, Han JS, Lee SH, Kim JM, Park JI, Park SK, Lim K, Hwang BD, Shong M, Kweon GR (2012). DJ-1 null dopaminergic neuronal cells exhibit defects in mitochondrial function and structure: involvement of mitochondrial complex I assembly. PLoS One.

[CR192] Hernán MA, Logroscino G, García Rodríguez LA (2006). Nonsteroidal anti-inflammatory drugs and the incidence of Parkinson disease. Neurology.

[CR193] Hernández AF, González-Alzaga B, López-Flores I, Lacasaña M (2016). Systematic reviews on neurodevelopmental and neurodegenerative disorders linked to pesticide exposure: Methodological features and impact on risk assessment. Environ Int.

[CR194] Hernández-Romero MC, Argüelles S, Villarán RF, de Pablos RM, Delgado-Cortés MJ, Santiago M, Herrera AJ, Cano J, Machado A (2008). Simvastatin prevents the inflammatory process and the dopaminergic degeneration induced by the intranigral injection of lipopolysaccharide. J Neurochem.

[CR195] Hernández-Romero MC, Delgado-Cortés MJ, Sarmiento M, de Pablos RM, Espinosa-Oliva AM, Argüelles S, Bández MJ, Villarán RF, Mauriño R, Santiago M, Venero JL, Herrera AJ, Cano J, Machado A (2012). Peripheral inflammation increases the deleterious effect of CNS inflammation on the nigrostriatal dopaminergic system. Neurotoxicology.

[CR196] Herrera AJ, Castaño A, Venero JL, Cano J, Machado A (2000). The single intranigral injection of LPS as a new model for studying the selective effects of inflammatory reactions on dopaminergic system. Neurobiol Dis.

[CR197] Herrero-Mendez A, Almeida A, Fernández E, Maestre C, Moncada S, Bolaños JP (2009). The bioenergetic and antioxidant status of neurons is controlled by continuous degradation of a key glycolytic enzyme by APC/C-Cdh1. Nat Cell Biol.

[CR198] Higgins DS, Greenamyre JT (1996). [3H]dihydrorotenone binding to NADH: ubiquinone reductase (complex I) of the electron transport chain: an autoradiographic study. J Neurosci.

[CR199] Hirata Y, Suzuno H, Tsuruta T, Oh-hashi K, Kiuchi K (2008). The role of dopamine transporter in selective toxicity of manganese and rotenone. Toxicology.

[CR200] Hirsch EC, Hunot S (2009). Neuroinflammation in Parkinson’s disease: a target for neuroprotection?. Lancet Neurol.

[CR201] Hirst J (2013). Mitochondrial complex I. Annu Rev Biochem.

[CR202] Hoefs SJ, Dieteren CE, Distelmaier F, Janssen RJ, Epplen A, Swarts HG, Forkink M, Rodenburg RJ, Nijtmans LG, Willems PH, Smeitink JA, van den Heuvel LP (2008). NDUFA2 complex I mutation leads to Leigh disease. Am J Hum Genet.

[CR203] Hoenen C, Gustin A, Birck C, Kirchmeyer M, Beaume N, Felten P, Grandbarbe L, Heuschling P, Heurtaux T (2016). Alpha-synuclein proteins promote pro-inflammatory cascades in microglia: stronger effects of the A53T mutant. PLoS One.

[CR204] Höglinger GU, Carrard G, Michel PP, Medja F, Lombès A, Ruberg M, Friguet B, Hirsch EC (2003). Dysfunction of mitochondrial complex I and the proteasome: interactions between two biochemical deficits in a cellular model of Parkinson’s disease. J Neurochem.

[CR205] Höglinger GU, Féger J, Prigent A, Michel PP, Parain K, Champy P, Ruberg M, Oertel WH, Hirsch EC (2003). Chronic systemic complex I inhibition induces a hypokinetic multisystem degeneration in rats. J Neurochem.

[CR206] Höllerhage M, Matusch A, Champy P, Lombès A, Ruberg M, Oertel WH, Höglinger GU (2009). Natural lipophilic inhibitors of mitochondrial complex I are candidate toxins for sporadic neurodegenerative tau pathologies. Exp Neurol.

[CR207] Hollingworth RM, Ahammadsahib KI, Gadelhak G, McLaughlin JL (1994). New inhibitors of complex I of the mitochondrial electron transport chain with activity as pesticides. Biochem Soc Trans.

[CR208] Hoos MD, Vitek MP, Ridnour LA, Wilson J, Jansen M, Everhart A, Wink DA, Colton CA (2014). The impact of human and mouse differences in NOS2 gene expression on the brain’s redox and immune environment. Mol Neurodegener.

[CR209] Horgan DJ, Casida JE (1968). Specific binding of [14C] piericidin A in the reduced nicotinamide–adenine dinucleotide dehydrogenase segment of the mitochondrial respiratory chain. Biochem J.

[CR210] Horgan DJ, Singer TP, Casida JE (1968). Studies on the respiratory chain-linked reduced nicotinamide adenine dinucleotide dehydrogenase. 13. Binding sites of rotenone, piericidin A, and amytal in the respiratory chain. J Biol Chem.

[CR211] Hsu LJ, Sagara Y, Arroyo A, Rockenstein E, Sisk A, Mallory M, Wong J, Takenouchi T, Hashimoto M, Masliah E (2000). alpha-synuclein promotes mitochondrial deficit and oxidative stress. Am J Pathol.

[CR212] Hu HI, Chang HH, Sun DS (2015). Differential regulation of caspase-2 in MPP^+^-induced apoptosis in primary cortical neurons. Exp Cell Res.

[CR213] Hung HC, Lee EH (1996). The mesolimbic dopaminergic pathway is more resistant than the nigrostriatal dopaminergic pathway to MPTP and MPP^+^ toxicity: role of BDNF gene expression. Brain Res Mol Brain Res.

[CR214] Hunot S, Boissière F, Faucheux B, Brugg B, Mouatt-Prigent A, Agid Y, Hirsch EC (1996). Nitric oxide synthase and neuronal vulnerability in Parkinson’s disease. Neuroscience.

[CR215] Hutchinson WD, Levy R, Dostrovsky JO, Lozano AM, Lang AE (1997). Effects of apomorphine on globus pallidus neurons in parkinsonian patients. Ann Neurol.

[CR216] Ichimaru N, Murai M, Kakutani N, Kako J, Ishihara A, Nakagawa Y, Nishioka T, Yagi T, Miyoshi H (2008). Synthesis and characterization of new piperazine-type inhibitors for mitochondrial NADH-ubiquinone oxidoreductase (complex I). Biochemistry.

[CR217] Li K, Ito H, Tanaka K, Hirano A (1997). Immunocytochemical co-localization of the proteasome in ubiquitinated structures in neurodegenerative diseases and the elderly. J Neuropathol Exp Neurol.

[CR218] Ino T, Nishioka T, Miyoshi H (2003). Characterization of inhibitor binding sites of mitochondrial complex I using fluorescent inhibitor. Biochim Biophys Acta.

[CR219] Irrcher I, Aleyasin H, Seifert EL, Hewitt SJ, Chhabra S, Phillips M, Lutz AK, Rousseaux MW, Bevilacqua L, Jahani-Asl A, Callaghan S, MacLaurin JG, Winklhofer KF, Rizzu P, Rippstein P, Kim RH, Chen CX, Fon EA, Slack RS, Harper ME, McBride HM, Mak TW, Park DS (2010). Loss of the Parkinson’s disease-linked gene DJ-1 perturbs mitochondrial dynamics. Hum Mol Genet.

[CR220] Ishii T, Sakurai T, Usami H, Uchida K (2005). Oxidative modification of proteasome: identification of an oxidation-sensitive subunit in 26 S proteasome. Biochemistry.

[CR221] Jackson-Lewis V, Jakowec M, Burke RE, Przedborski S (1995). Time course and morphology of dopaminergic neuronal death caused by the neurotoxin 1-methyl-4-phenyl-1,2,3,6-tetrahydropyridine. Neurodegeneration.

[CR222] Jakowec MW, Nixon K, Hogg E, McNeill T, Petzinger GM (2004). Tyrosine hydroxylase and dopamine transporter expression following 1-methyl-4-phenyl-1,2,3,6-tetrahydropyridine-induced neurodegeneration of the mouse nigrostriatal pathway. J Neurosci Res.

[CR223] Jana S, Sinha M, Chanda D, Roy T, Banerjee K, Munshi S, Patro BS, Chakrabarti S (2011). Mitochondrial dysfunction mediated by quinone oxidation products of dopamine: Implications in dopamine cytotoxicity and pathogenesis of Parkinson’s disease. Biochim Biophys Acta.

[CR224] Jankovic J (2008). Parkinson’s disease and movement disorders: moving forward. Lancet Neurol.

[CR225] Janssen RJ, Nijtmans LG, van den Heuvel LP, Smeitink JA (2006). Mitochondrial complex I: structure, function and pathology. J Inherit Metab Dis.

[CR226] Jenner P (2008). Functional models of Parkinson’s disease: a valuable tool in the development of novel therapies. Ann Neurol.

[CR227] Jha N, Jurma O, Lalli G, Liu Y, Pettus EH, Greenamyre JT, Liu RM, Forman HJ, Andersen JK (2000). Glutathione depletion in PC12 results in selective inhibition of mitochondrial complex I activity. Implications for Parkinson’s disease. J Biol Chem.

[CR228] Joel D, Weiner I (2000). The connections of the dopaminergic system with the striatum in rats and primates: an analysis with respect to the functional and compartmental organization of the striatum. Neuroscience.

[CR229] Jones BJ, Roberts DJ (1968). The quantiative measurement of motor inco-ordination in naive mice using an acelerating rotarod. J Pharm Pharmacol.

[CR230] Kadowaki M, Karim MR (2009). Cytosolic LC3 ratio as a quantitative index of macroautophagy. Methods Enzymol.

[CR231] Kean EA, Gutman M, Singer TP (1971). Studies on the respiratory chain-linked nicotinamide adenine dinucleotide dehydrogenase. XXII. Rhein, a competitive inhibitor of the dehydrogenase. J Biol Chem.

[CR232] Khaliq ZM, Bean BP (2010). Pacemaking in dopaminergic ventral tegmental area neurons: depolarizing drive from background and voltage-dependent sodium conductances. J Neurosci.

[CR233] Khan FH, Sen T, Maiti AK, Jana S, Chatterjee U, Chakrabarti S (2005). Inhibition of rat brain mitochondrial electron transport chain activity by dopamine oxidation products during extended in vitro incubation: implications for Parkinson’s disease. Biochim Biophys Acta.

[CR234] Khan TA, Hassan I, Ahmad A, Perveen A, Aman S, Quddusi S, Alhazza IM, Ashraf GM1, Aliev G (2016). Recent updates on the dynamic association between oxidative stress and neurodegenerative disorders. CNS Neurol Disord Drug Targets.

[CR235] Kilpatrick K, Zeng Y, Hancock T, Segatori L (2015). Genetic and chemical activation of TFEB mediates clearance of aggregated α-synuclein. PLoS One.

[CR236] Kim HM, Yu Y, Cheng Y (2011). Structure characterization of the 26S proteasome. Biochim Biophys Acta.

[CR237] Kinugawa K, Monnet Y, Béchade C, Alvarez-Fischer D, Hirsch EC, Bessis A, Hunot S (2013). DAP12 and CD11b contribute to the microglial-induced death of dopaminergic neurons in vitro but not in vivo in the MPTP mouse model of Parkinson’s disease. J Neuroinflammation.

[CR238] Kirby DM, Thorburn DR, Turnbull DM, Taylor RW (2007). Biochemical assays of respiratory chain complex activity. Methods Cell Biol.

[CR239] Kirik D, Rosenblad C, Björklund A (1998). Characterization of behavioral and neurodegenerative changes following partial lesions of the nigrostriatal dopamine system induced by intrastriatal 6-hydroxydopamine in the rat. Exp Neurol.

[CR240] Kirik D, Annett LE, Burger C, Muzyczka N, Mandel RJ, Björklund A (2003). Nigrostriatal alpha-synucleinopathy induced by viral vector-mediated overexpression of human alpha-synuclein: a new primate model of Parkinson’s disease. Proc Natl Acad Sci USA.

[CR241] Kisselev AF, Goldberg AL (2005). Monitoring activity and inhibition of 26S proteasomes with fluorogenic peptide substrates. Methods Enzymol.

[CR242] Kitada T, Asakawa S, Hattori N, Matsumine H, Yamamura Y, Minoshima S, Yokochi M, Mizuno Y, Shimizu N (1998). Mutations in the parkin gene cause autosomal recessive juvenile parkinsonism. Nature.

[CR243] Klapdor K, Dulfer BG, Hammann A, Van der Staay FJ (1997). A low-cost method to analyse footprint patterns. J Neurosci Methods.

[CR244] Klionsky DJ (2008). Guidelines for the use and interpretation of assays for monitoring autophagy in higher eukaryotes. Autophagy.

[CR245] Knott C, Stern G, Wilkin GP (2000). Inflammatory regulators in Parkinson’s disease: iNOS, lipocortin-1, and cyclooxygenases-1 and -2. Mol Cell Neurosci.

[CR246] Koga K, Mori A, Ohashi S, Kurihara N, Kitagawa H, Ishikawa M, Mitsumoto Y, Nakai M (2006). H MRS identifies lactate rise in the striatum of MPTP-treated C57BL/6 mice. Eur J Neurosci.

[CR247] Koizumi S, Shigemoto-Mogami Y, Nasu-Tada K, Shinozaki Y, Ohsawa K, Tsuda M, Joshi BV, Jacobson KA, Kohsaka S, Inoue K (2007). UDP acting at P2Y6 receptors is a mediator of microglial phagocytosis. Nature.

[CR248] Koller WC (1992). When does Parkinson’s disease begin?. Neurology.

[CR249] Komatsu M, Waguri S, Chiba T, Murata S, Iwata J, Tanida I, Ueno T, Koike M, Uchiyama Y, Kominami E, Tanaka K (2006). Loss of autophagy in the central nervous system causes neurodegeneration in mice. Nature.

[CR250] Kong P, Zhang B, Lei P, Kong X, Zhang S, Li D, Zhang Y (2015). Neuroprotection of MAO-B inhibitor and dopamine agonist in Parkinson disease. Int J Clin Exp Med.

[CR251] Koopman WJ, Verkaart S, Visch HJ, van Emst-de Vries S, Nijtmans LG, Smeitink JA, Willems PH (2007). Human NADH:ubiquinone oxidoreductase deficiency: radical changes in mitochondrial morphology?. Am J Physiol Cell Physiol.

[CR252] Kordower JH, Freeman TB, Snow BJ, Vingerhoets FJ, Mufson EJ, Sanberg PR, Hauser RA, Smith DA, Nauert GM, Perl DP (1995). Neuropathological evidence of graft survival and striatal reinnervation after the transplantation of fetal mesencephalic tissue in a patient with Parkinson’s disease. N Engl J Med.

[CR253] Kordower JH, Freeman TB, Chen EY, Mufson EJ, Sanberg PR, Hauser RA, Snow B, Olanow CW (1998). Fetal nigral grafts survive and mediate clinical benefit in a patient with Parkinson’s disease. Mov Disord.

[CR254] Korner G, Noain D, Ying M, Hole M, Flydal MI, Scherer T, Allegri G, Rassi A, Fingerhut R, Becu-Villalobos D, Pillai S, Wueest S, Konrad D, Lauber-Biason A, Baumann CR, Bindoff LA, Martinez A, Thöny B (2015). Brain catecholamine depletion and motor impairment in a Th knock-in mouse with type B tyrosine hydroxylase deficiency. Brain.

[CR255] Korolchuk VI, Menzies FM, Rubinsztein DC (2010). Mechanisms of cross-talk between the ubiquitin-proteasome and autophagy-lysosome systems. FEBS Lett.

[CR256] Kotlyar AB, Sled VD, Burbaev DS, Moroz IA, Vinogradov AD (1990). Coupling site I and the rotenone-sensitive ubisemiquinone in tightly coupled submitochondrial particles. FEBS Lett.

[CR257] Kraft AD, Harry GJ (2011). Features of microglia and neuroinflammation relevant to environmental exposure and neurotoxicity. Int J Environ Res Public Health.

[CR258] Kroemer G, Mariño G, Levine B (2010). Autophagy and the integrated stress response. Mol Cell.

[CR259] Kubota NK, Ohta E, Ohta S, Koizumi F, Suzuki M, Ichimura M, Ikegami S (2003). Piericidins C5 and C6: new 4-pyridinol compounds produced by* Streptomyces* sp. and* Nocardioides* sp. Bioorg Med Chem.

[CR260] Kuegler PB, Zimmer B, Waldmann T, Baudis B, Ilmjärv S, Hescheler J, Gaughwin P, Brundin P, Mundy W, Bal-Price AK, Schrattenholz A, Krause KH, van Thriel C, Rao MS, Kadereit S, Leist M (2010). Markers of murine embryonic and neural stem cells, neurons and astrocytes: reference points for developmental neurotoxicity testing. ALTEX.

[CR261] Kuma A, Hatano M, Matsui M, Yamamoto A, Nakaya H, Yoshimori T, Ohsumi Y, Tokuhisa T, Mizushima N (2004). The role of autophagy during the early neonatal starvation period. Nature.

[CR262] Kuoppamäki M, Al-Barghouthy G, Jackson MJ, Smith LA, Quinn N, Jenner P (2007). L-dopa dose and the duration and severity of dyskinesia in primed MPTP-treated primates. J Neural Transm (Vienna).

[CR263] Lambert AJ, Brand MD (2004). Inhibitors of the quinone-binding site allow rapid superoxide production from mitochondrial NADH:ubiquinone oxidoreductase (complex I). J Biol Chem.

[CR264] Langston JW, Forno LS, Tetrud J, Reeves AG, Kaplan JA, Karluk D (1999). Evidence of active nerve cell degeneration in the substantia nigra of humans years after 1-methyl-4-phenyl-1,2,3,6-tetrahydropyridine exposure. Ann Neurol.

[CR265] Langston JW, Quik M, Petzinger G, Jakowec M, Di Monte DA (2000). Investigating levodopa-induced dyskinesias in the parkinsonian primate. Ann Neurol.

[CR266] Laurie C, Reynolds A, Coskun O, Bowman E, Gendelman HE, Mosley RL (2007) CD4^+^ T cells from Copolymer-1 immunized mice protect dopaminergic neurons in the 1-methyl-4-phenyl-1,2,3,6-tetrahydropyridine model of Parkinson’s disease. J Neuroimmunol 183(1–2):60–68 10.1016/j.jneuroim.2006.11.009 17196666

[CR267] Lazarou M, Thorburn DR, Ryan MT, McKenzie M (2009). Assembly of mitochondrial complex I and defects in disease. Biochim Biophys Acta.

[CR268] Le WD, Rowe DB, Jankovic J, Xie W, Appel SH (1999). Effects of cerebrospinal fluid from patients with Parkinson disease on dopaminergic cells. Arch Neurol.

[CR269] Lecca D, Nevin DK, Mulas G, Casu MA, Diana A, Rossi D, Sacchetti G, Carta AR (2015). Neuroprotective and anti-inflammatory properties of a novel non-thiazolidinedione PPARγ agonist in vitro and in MPTP-treated mice. Neuroscience.

[CR270] Lee HJ, Khoshaghideh F, Lee S, Lee SJ (2006). Impairment of microtubule-dependent trafficking by overexpression of alpha-synuclein. Eur J Neurosci.

[CR271] Lee HJ, Kim C, Lee SJ (2010). Alpha-synuclein stimulation of astrocytes: Potential role for neuroinflammation and neuroprotection. Oxid Med Cell Longev.

[CR272] Lee HJ, Suk JE, Patrick C, Bae EJ, Cho JH, Rho S, Hwang D, Masliah E, Lee SJ (2010). Direct transfer of alpha-synuclein from neuron to astroglia causes inflammatory responses in synucleinopathies. J Biol Chem.

[CR273] Lee DH, Kim CS, Lee YJ (2011). Astaxanthin protects against MPTP/MPP^+^-induced mitochondrial dysfunction and ROS production in vivo and in vitro. Food Chem Toxicol.

[CR274] Lee J, Giordano S, Zhang J (2012). Autophagy, mitochondria and oxidative stress: cross-talk and redox signalling. Biochem J.

[CR275] Leenders KL, Poewe WH, Palmer AJ, Brenton DP, Frackowiak RS (1986). Inhibition of l-[^18^F]fluorodopa uptake into human brain by amino acids demonstrated by positron emission tomography. Ann Neurol.

[CR276] Leist M, Single B, Castoldi AF, Kühnle S, Nicotera P (1997). Intracellular adenosine triphosphate (ATP) concentration: a switch in the decision between apoptosis and necrosis. J Exp Med.

[CR277] Leist M, Volbracht C, Fava E, Nicotera P (1998). 1-Methyl-4-phenylpyridinium induces autocrine excitotoxicity, protease activation, and neuronal apoptosis. Mol Pharmacol.

[CR278] Leist M, Ghallab A, Graepel R, Marchan R, Hassan R, Bennekou SH, Limonciel A, Vinken M, Schildknecht S, Waldmann T, Danen E, van Ravenzwaay B, Kamp H, Gardner I, Godoy P, Bois FY, Braeuning A, Reif R, Oesch F, Drasdo D, Höhme S, Schwarz M, Hartung T, Braunbeck T, Beltman J, Vrieling H, Sanz F, Forsby A, Gadaleta D, Fisher C, Kelm J, Fluri D, Ecker G, Zdrazil B, Terron A, Jennings P, van der Burg B, Dooley S, Meijer AH, Willighagen E, Martens M, Evelo C, Mombelli E, Taboureau O, Mantovani A, Hardy B, Koch B, Escher S, van Thriel C, Cadenas C, Kroese D, van de Water B, Hengstler JG (2017) Adverse outcome pathways: opportunities, limitations and open questions. Arch Toxicol. 10.1007/s00204-017-2045-3 10.1007/s00204-017-2045-3 29051992

[CR279] Lemasters JJ, Theruvath TP, Zhong Z, Nieminen AL (2009). Mitochondrial calcium and the permeability transition in cell death. Biochim Biophys Acta.

[CR280] Lenaz G, Fato R, Baracca A, Genova ML (2004). Mitochondrial quinone reductases: complex I. Methods Enzymol.

[CR281] Leng A, Mura A, Feldon J, Ferger B (2005). Tumor necrosis factor-alpha receptor ablation in a chronic MPTP mouse model of Parkinson’s disease. Neurosci Lett.

[CR282] Leroy E, Boyer R, Auburger G, Leube B, Ulm G, Mezey E, Harta G, Brownstein MJ, Jonnalagada S, Chernova T, Dehejia A, Lavedan C, Gasser T, Steinbach PJ, Wilkinson KD, Polymeropoulos MH (1998). The ubiquitin pathway in Parkinson’s disease. Nature.

[CR283] Levy R, Dostrovsky JO, Lang AE, Sime E, Hutchison WD, Lozano AM (2001). Effects of apomorphine on subthalamic nucleus and globus pallidus internus neurons in patients with Parkinson’s disease. J Neurophysiol.

[CR284] Li XP, Xie WJ, Zhang Z, Kansara S, Jankovic J, Le WD (2012). A mechanistic study of proteasome inhibition-induced iron misregulation in dopamine neuron degeneration. Neurosignals.

[CR285] Li L, Nadanaciva S, Berger Z, Shen W, Paumier K, Schwartz J, Mou K, Loos P, Milici AJ, Dunlop J, Hirst WD (2013). Human A53T α-synuclein causes reversible deficits in mitochondrial function and dynamics in primary mouse cortical neurons. PLoS One.

[CR286] Li DW, Yao M, Dong YH, Tang MN, Chen W, Li GR, Sun BQ (2014). Guanosine exerts neuroprotective effects by reversing mitochondrial dysfunction in a cellular model of Parkinson’s disease. Int J Mol Med.

[CR287] Liberatore GT, Jackson-Lewis V, Vukosavic S, Mandir AS, Vila M, McAuliffe WG, Dawson VL, Dawson TM, Przedborski S (1999) Inducible nitric oxide synthase stimulates dopaminergic neurodegeneration in the MPTP model of Parkinson disease. 5(12):1403–1409 10.1038/70978 10581083

[CR288] Liddelow SA, Guttenplan KA, Clarke LE, Bennett FC, Bohlen CJ, Schirmer L, Bennett ML, Münch AE, Chung WS, Peterson TC, Wilton DK, Frouin A, Napier BA, Panicker N, Kumar M, Buckwalter MS, Rowitch DH, Dawson VL, Dawson TM, Stevens B, Barres BA (2017). Neurotoxic reactive astrocytes are induced by activated microglia. Nature.

[CR289] Lim J, Kim HW, Youdim MB, Rhyu IJ, Choe KM, Oh YJ (2011). Binding preference of p62 towards LC3-ll during dopaminergic neurotoxin-induced impairment of autophagic flux. Autophagy.

[CR290] Lin MT, Beal MF (2006). Mitochondrial dysfunction and oxidative stress in neurodegenerative diseases. Nature.

[CR291] Lin SC, Lin KJ, Hsiao IT, Hsieh CJ, Lin WY, Lu CS, Wey SP, Yen TC, Kung MP, Weng YH (2014). In vivo detection of monoaminergic degeneration in early Parkinson disease by (18)F-9-fluoropropyl-(+)-dihydrotetrabenzazine PET. J Nucl Med.

[CR292] Liu Y, Fiskum G, Schubert D (2002). Generation of reactive oxygen species by the mitochondrial electron transport chain. J Neurochem.

[CR293] Liu S, Liu Y, Hao W, Wolf L, Kiliaan AJ, Penke B, Rübe CE, Walter J, Heneka MT, Hartmann T, Menger MD, Fassbender K (2012). TLR2 is a primary receptor for Alzheimer’s amyloid β peptide to trigger neuroinflammatory activation. J Immunol.

[CR294] Liu K, Shi N, Sun Y, Zhang T, Sun X (2013). Therapeutic effects of rapamycin on MPTP-induced Parkinsonism in mice. Neurochem Res.

[CR295] Liu W, Kong S, Xie Q, Su J, Li W, Guo H, Li S, Feng X, Su Z, Xu Y, Lai X (2015). Protective effects of apigenin against 1-methyl-4-phenylpyridinium ion‑induced neurotoxicity in PC12 cells. Int J Mol Med.

[CR296] Liu Z, Chen HQ, Huang Y, Qiu YH, Peng YP (2016). Transforming growth factor-β1 acts via TβR-I on microglia to protect against MPP(+)-induced dopaminergic neuronal loss. Brain Behav Immun.

[CR297] Llaudet E, Hatz S, Droniou M, Dale N (2005). Microelectrode biosensor for real-time measurement of ATP in biological tissue. Anal Chem.

[CR298] Lloyd KG, Davidson L, Hornykiewicz O (1975). The neurochemistry of Parkinson’s disease: effect of L-dopa therapy. J Pharmacol Exp Ther.

[CR299] Long J, Ma J, Luo C, Mo X, Sun L, Zang W, Liu J (2009). Comparison of two methods for assaying complex I activity in mitochondria isolated from rat liver, brain and heart. Life Sci.

[CR300] Lopategui Cabezas I, Herrera Batista A, Pentón Rol G (2014). The role of glial cells in Alzheimer disease: potential therapeutic implications. Neurologia.

[CR301] Lopez-Ramirez MA, Wu D, Pryce G, Simpson JE, Reijerkerk A, King-Robson J, Kay O, de Vries HE, Hirst MC, Sharrack B, Baker D, Male DK, Michael GJ, Romero IA (2014). MicroRNA-155 negatively affects blood-brain barrier function during neuroinflammation. FASEB J.

[CR302] Lotharius J, Barg S, Wiekop P, Lundberg C, Raymon HK, Brundin P (2002). Effect of mutant alpha-synuclein on dopamine homeostasis in a new human mesencephalic cell line. J Biol Chem.

[CR303] Lu X, Bing G, Hagg T (2000). Naloxone prevents microglia-induced degeneration of dopaminergic substantia nigra neurons in adult rats. Neuroscience.

[CR304] Lümmen P (1998). Complex I inhibitors as insecticides and acaricides. Biochim Biophys Acta.

[CR305] Lynd-Balta E, Haber SN (1994). The organization of midbrain projections to the striatum in the primate: sensorimotor-related striatum versus ventral striatum. Neuroscience.

[CR306] Lynd-Balta E, Haber SN (1994). The organization of midbrain projections to the ventral striatum in the primate. Neuroscience.

[CR307] Ma CP, Slaughter CA, DeMartino GN (1992). Identification, purification, and characterization of a protein activator (PA28) of the 20 S proteasome (macropain). J Biol Chem.

[CR308] Mader BJ, Pivtoraiko VN, Flippo HM, Klocke BJ, Roth KA, Mangieri LR, Shacka JJ (2012). Rotenone inhibits autophagic flux prior to inducing cell death. ACS Chem Neurosci.

[CR309] Mailloux RJ (2015) Teaching the fundamentals of electron transfer reactions in mitochondria and the production and detection of reactive oxygen species. Redox Biol 4:381–398 10.1016/j.redox.2015.02.001 PMC4348434 25744690

[CR310] Marella M, Seo BB, Nakamaru-Ogiso E, Greenamyre JT, Matsuno-Yagi A, Yagi T (2008). Protection by the NDI1 gene against neurodegeneration in a rotenone rat model of Parkinson’s disease. PLoS One.

[CR311] Marques O, Outeiro TF (2012). Alpha-synuclein: from secretion to dysfunction and death. Cell Death Dis.

[CR312] Marshall LE, Himes RH (1978). Rotenone inhibition of tubulin self-assembly. Biochim Biophys Acta.

[CR313] Martinez-Vicente M, Talloczy Z, Kaushik S, Massey AC, Mazzulli J, Mosharov EV, Hodara R, Fredenburg R, Wu DC, Follenzi A, Dauer W, Przedborski S, Ischiropoulos H, Lansbury PT, Sulzer D, Cuervo AM (2008). Dopamine-modified alpha-synuclein blocks chaperone-mediated autophagy. J Clin Investig.

[CR314] Martini-Stoica H, Xu Y, Ballabio A, Zheng H (2016). The Autophagy-Lysosomal Pathway in Neurodegeneration: A TFEB Perspective. Trends Neurosci.

[CR315] Masliah E, Rockenstein E, Veinbergs I, Mallory M, Hashimoto M, Takeda A, Sagara Y, Sisk A, Mucke L (2000). Dopaminergic loss and inclusion body formation in alpha-synuclein mice: implications for neurodegenerative disorders. Science.

[CR316] Matsuda W, Furuta T, Nakamura KC, Hioki H, Fujiyama F, Arai R, Kaneko T (2009). Single nigrostriatal dopaminergic neurons form widely spread and highly dense axonal arborizations in the neostriatum. J Neurosci.

[CR317] Matthews RT, Ferrante RJ, Klivenyi P, Yang L, Klein AM, Mueller G, Kaddurah-Daouk R, Beal MF (1999). Creatine and cyclocreatine attenuate MPTP neurotoxicity. Exp Neurol.

[CR318] McCord JM, Fridovich I (1968). The reduction of cytochrome c by milk xanthine oxidase. J Biol Chem.

[CR319] McCoy MK, Martinez TN, Ruhn KA, Szymkowski DE, Smith CG, Botterman BR, Tansey KE, Tansey MG (2006). Blocking soluble tumor necrosis factor signaling with dominant-negative tumor necrosis factor inhibitor attenuates loss of dopaminergic neurons in models of Parkinson’s disease. J Neurosci.

[CR320] McGeer PL, McGeer EG (2008). Glial reactions in Parkinson’s disease. Mov Disord.

[CR321] McGeer PL, Itagaki S, Boyes BE, McGeer EG (1988). Reactive microglia are positive for HLA-DR in the substantia nigra of Parkinson’s and Alzheimer’s disease brains. Neurology.

[CR322] McGeer PL, Schwab C, Parent A, Doudet D (2003). Presence of reactive microglia in monkey substantia nigra years after 1-methyl-4-phenyl-1,2,3,6-tetrahydropyridine administration. Ann Neurol.

[CR323] McNaught KS, Jenner P (2001). Proteasomal function is impaired in substantia nigra in Parkinson’s disease. Neurosci Lett.

[CR324] McNaught KS, Olanow CW, Halliwell B, Isacson O, Jenner P (2001). Failure of the ubiquitin-proteasome system in Parkinson’s disease. Nat Rev Neurosci.

[CR325] McNaught KS, Belizaire R, Isacson O, Jenner P, Olanow CW (2003). Altered proteasomal function in sporadic Parkinson’s disease. Exp Neurol.

[CR326] Melo TQ, van Zomeren KC, Ferrari MF, Boddeke HW, Copray JC (2017). Impairment of mitochondria dynamics by human A53T α-synuclein and rescue by NAP (davunetide) in a cell model for Parkinson’s disease. Exp Brain Res.

[CR327] Mendez I, Viñuela A, Astradsson A, Mukhida K, Hallett P, Robertson H, Tierney T, Holness R, Dagher A, Trojanowski JQ, Isacson O (2008). Dopamine neurons implanted into people with Parkinson’s disease survive without pathology for 14 years. Nat Med.

[CR328] Menzies FM, Fleming A, Rubinsztein DC (2015). Compromised autophagy and neurodegenerative diseases. Nat Rev Neurosci.

[CR329] Meredith GE, Kang UJ (2006). Behavioral models of Parkinson’s disease in rodents: a new look at an old problem. Mov Disord.

[CR330] Miklossy J, Doudet DD, Schwab C, Yu S, McGeer EG, McGeer PL (2006). Role of ICAM-1 in persisting inflammation in Parkinson disease and MPTP monkeys. Exp Neurol.

[CR331] Mitchell IJ, Clarke CE, Boyce S, Robertson RG, Peggs D, Sambrook MA, Crossman AR (1989). Neural mechanisms underlying parkinsonian symptoms based upon regional uptake of 2-deoxyglucose in monkeys exposed to 1-methyl-4-phenyl-1,2,3,6-tetrahydropyridine. Neuroscience.

[CR332] Miyoshi H (1998). Structure-activity relationships of some complex I inhibitors. Biochim Biophys Acta.

[CR333] Mizushima N, Levine B, Cuervo AM, Klionsky DJ (2008). Autophagy fights disease through cellular self-digestion. Nature.

[CR334] Moehle MS, West AB (2015) M1 and M2 immune activation in Parkinson’s disease: foe and ally? Neuroscience 302:59–73 10.1016/j.neuroscience.2014.11.018 PMC4442748 25463515

[CR335] Mogi M, Harada M, Riederer P, Narabayashi H, Fujita K, Nagatsu T (1994) Tumor necrosis factor-alpha (TNF-alpha) increases both in the brain and in the cerebrospinal fluid from parkinsonian patients. Neurosci Lett 165(1–2):208–210 10.1016/0304-3940(94)90746-3 8015728

[CR336] Monnet-Tschudi F, Zurich MG, Honegger P (2007). Neurotoxicant-induced inflammatory response in three-dimensional brain cell cultures. Hum Exp Toxicol.

[CR337] Monnet-Tschudi F, Defaux A, Braissant O, Cagnon L, Zurich MG (2011). Methods to assess neuroinflammation. Curr Protoc Toxicol.

[CR338] Moon Y, Lee KH, Park JH, Geum D, Kim K (2005). Mitochondrial membrane depolarization and the selective death of dopaminergic neurons by rotenone: protective effect of coenzyme Q10. J Neurochem.

[CR339] Moon M, Kim HG, Hwang L, Seo JH, Kim S, Hwang S, Kim S, Lee D, Chung H, Oh MS, Lee KT, Park S (2009). Neuroprotective effect of ghrelin in the 1-methyl-4-phenyl-1,2,3,6-tetrahydropyridine mouse model of Parkinson’s disease by blocking microglial activation. Neurotox Res.

[CR340] Moratalla R, Quinn B, DeLanney LE, Irwin I, Langston JW, Graybiel AM (1992). Differential vulnerability of primate caudate-putamen and striosome-matrix dopamine systems to the neurotoxic effects of 1-methyl-4-phenyl-1,2,3,6-tetrahydropyridine. Proc Natl Acad Sci U S A.

[CR341] Morikawa N, Nakagawa-Hattori Y, Mizuno Y (1996). Effect of dopamine, dimethoxyphenylethylamine, papaverine, and related compounds on mitochondrial respiration and complex I activity. J Neurochem.

[CR342] Mount MP, Lira A, Grimes D, Smith PD, Faucher S, Slack R, Anisman H, Hayley S, Park DS (2007). Involvement of interferon-gamma in microglial-mediated loss of dopaminergic neurons. J Neurosci.

[CR343] Munafó DB, Colombo MI (2002). Induction of autophagy causes dramatic changes in the subcellular distribution of GFP-Rab24. Traffic.

[CR344] Murata S, Yashiroda H, Tanaka K (2009). Molecular mechanisms of proteasome assembly. Nat Rev Mol Cell Biol.

[CR345] Muthane U, Ramsay KA, Jiang H, Jackson-Lewis V, Donaldson D, Fernando S, Ferreira M, Przedborski S (1994). Differences in nigral neuron number and sensitivity to 1-methyl-4-phenyl-1,2,3,6-tetrahydropyridine in C57/bl and CD-1 mice. Exp Neurol.

[CR346] Nakai M, Mori A, Watanabe A, Mitsumoto Y (2003). 1-methyl-4-phenylpyridinium (MPP^+^) decreases mitochondrial oxidation–reduction (REDOX) activity and membrane potential (Deltapsi(m)) in rat striatum. Exp Neurol.

[CR347] Nakajima K, Kohsaka S (2004). Microglia: neuroprotective and neurotrophic cells in the central nervous system. Curr Drug Targets Cardiovasc Haematol Disord.

[CR348] Nataraj J, Manivasagam T, Thenmozhi AJ, Essa MM (2016). Lutein protects dopaminergic neurons against MPTP-induced apoptotic death and motor dysfunction by ameliorating mitochondrial disruption and oxidative stress. Nutr Neurosci.

[CR349] Nath S, Goodwin J, Engelborghs Y, Pountney DL (2011). Raised calcium promotes α-synuclein aggregate formation. Mol Cell Neurosci.

[CR350] Nayak A, Ansar R, Verma SK, Bonifati DM, Kishore U (2011) Huntington’s Disease: An Immune Perspective. Neurol Res Int 2011:563784 10.1155/2011/563784 PMC3163125 21876800

[CR351] Nedergaard S, Flatman JA, Engberg I (1993). Nifedipine- and omega-conotoxin-sensitive Ca2 + conductances in guinea-pig substantia nigra pars compacta neurones. J Physiol.

[CR352] Nguyen VT, Morange M, Bensaude O (1988). Firefly luciferase luminescence assays using scintillation counters for quantitation in transfected mammalian cells. Anal Biochem.

[CR353] Nicklas WJ, Vyas I, Heikkila RE (1985). Inhibition of NADH-linked oxidation in brain mitochondria by 1-methyl-4-phenyl-pyridine, a metabolite of the neurotoxin, 1-methyl-4-phenyl-1,2,5,6-tetrahydropyridine. Life Sci.

[CR354] NINDS NET-PD Investigators (2006). A randomized, double-blind, futility clinical trial of creatine and minocycline in early Parkinson disease. Neurology.

[CR355] NINDS NET-PD Investigators (2008). A pilot clinical trial of creatine and minocycline in early Parkinson disease: 18-month results. Clin Neuropharmacol.

[CR356] Noelker C, Morel L, Lescot T, Osterloh A, Alvarez-Fischer D, Breloer M, Henze C, Depboylu C, Skrzydelski D, Michel PP, Dodel RC, Lu L, Hirsch EC, Hunot S, Hartmann A (2013). Toll like receptor 4 mediates cell death in a mouse MPTP model of Parkinson disease. Sci Rep.

[CR357] Norden DM, Muccigrosso MM, Godbout JP (2015). Microglial priming and enhanced reactivity to secondary insult in aging, and traumatic CNS injury, and neurodegenerative disease. Neuropharmacology.

[CR358] O’Malley KL (2010). The role of axonopathy in Parkinson’s disease. Exp Neurobiol.

[CR359] Obeso JA, Marin C, Rodriguez-Oroz C, Blesa J, Benitez-Temiño B, Mena-Segovia J, Rodríguez M, Olanow CW (2008). The basal ganglia in Parkinson’s disease: current concepts and unexplained observations. Ann Neurol.

[CR360] Obeso JA, Rodríguez-Oroz MC, Benitez-Temino B, Blesa FJ, Guridi J, Marin C, Rodriguez M (2008). Functional organization of the basal ganglia: therapeutic implications for Parkinson’s disease. Mov Disord.

[CR361] Ockleford C, Adriaanse P, Berny P, Brock T, Duquesne S, Grilli S, Hernandez-Jerez AF, Bennekou SH, Klein M, Kuhl T, Laskowski R, Machera K, Pelkonen O, Pieper S, Smith R, Stemmer M, Sundh I, Teodorovic I, Tiktak A, Topping CJ, Wolterink G, Angeli K, Fritsche E, Hernandez-Jerez AF, Leist M, Mantovani A, Menendez P, Pelkonen O, Price A, Viviani B, Chiusolo A, Ruffo F, Terron A, Bennekou SH (2017). Scientific opinion on the investigation into experimental toxicological properties of plant protection products having a potential link to Parkinson’s disease and childhood leukaemia. EFSA J.

[CR362] Odekerken VJ, van Laar T, Staal MJ, Mosch A, Hoffmann CF, Nijssen PC, Beute GN, van Vugt JP, Lenders MW, Contarino MF, Mink MS, Bour LJ, van den Munckhof P, Schmand BA, de Haan RJ, Schuurman PR, de Bie RM (2013). Subthalamic nucleus versus globus pallidus bilateral deep brain stimulation for advanced Parkinson’s disease (NSTAPS study): a randomised controlled trial. Lancet Neurol.

[CR363] Ogilvie I, Kennaway NG, Shoubridge EA (2005). A molecular chaperone for mitochondrial complex I assembly is mutated in a progressive encephalopathy. J Clin Investig.

[CR364] Ohnishi T (1998). Iron-sulfur clusters/semiquinones in complex I. Biochim Biophys Acta.

[CR365] Okun JG, Lümmen P, Brandt U (1999). Three classes of inhibitors share a common binding domain in mitochondrial complex I (NADH:ubiquinone oxidoreductase). J Biol Chem.

[CR366] Olanow CW, Hauser RA, Jankovic J, Langston W, Lang A, Poewe W, Tolosa E, Stocchi F, Melamed E, Eyal E, Rascol O (2008). A randomized, double-blind, placebo-controlled, delayed start study to assess rasagiline as a disease modifying therapy in Parkinson’s disease (the ADAGIO study): rationale, design, and baseline characteristics. Mov Disord.

[CR367] Orrenius S, Zhivotovsky B, Nicotera P (2003). Regulation of cell death: the calcium-apoptosis link. Nat Rev Mol Cell Biol.

[CR368] Ortega Z, Lucas JJ (2014). Ubiquitin-proteasome system involvement in Huntington’s disease. Front Mol Neurosci.

[CR369] Pacelli C, Giguère N, Bourque MJ, Lévesque M, Slack RS, Trudeau L (2015). Elevated mitochondrial bioenergetics and axonal arborization size are key contributors to the vulnerability of dopamine neurons. Curr Biol.

[CR370] Pagliarini DJ, Calvo SE, Chang B, Sheth SA, Vafai SB, Ong SE, Walford GA, Sugiana C, Boneh A, Chen WK, Hill DE, Vidal M, Evans JG, Thorburn DR, Carr SA, Mootha VK (2008). A mitochondrial protein compendium elucidates complex I disease biology. Cell.

[CR371] Pålhagen S, Heinonen EH, Hägglund J, Kaugesaar T, Kontants H, Mäki-Ikola O, Palm R, Turunen J (1998). Selegiline delays the onset of disability in de novo parkinsonian patients. Neurology.

[CR372] Pålhagen S, Heinonen E, Hägglund J, Kaugesaar T, Mäki-Ikola O, Palm R, Swedish Parkinson Study Group (2006). Selegiline slows the progression of the symptoms of Parkinson disease. Neurology.

[CR373] Palmer G, Horgan DJ, Tisdale H, Singer TP, Beinert H (1968). Studies on the respiratory chain-linked reduced nicotinamide adenine dinucleotide dehydrogenase. XIV. Location of the sites of inhibition of rotenone, barbiturates, and piericidin by means of electron paramagnetic resonance spectroscopy. J Biol Chem.

[CR374] Pan W, Kastin AJ (2002). TNFalpha transport across the blood-brain barrier is abolished in receptor knockout mice. Exp Neurol.

[CR375] Pan T, Rawal P, Wu Y, Xie W, Jankovic J, Le W (2009). Rapamycin protects against rotenone-induced apoptosis through autophagy induction. Neuroscience.

[CR376] Papa SM, Desimone R, Fiorani M, Oldfield EH (1999). Internal globus pallidus discharge is nearly suppressed during levodopa-induced dyskinesias. Ann Neurol.

[CR377] Parkinson Study Group (1993). Effects of tocopherol and deprenyl on the progression of disability in early Parkinson’s disease. N Engl J Med.

[CR378] Parkinson Study Group (1996). Effect of lazabemide on the progression of disability in early Parkinson’s disease. The Parkinson Study Group. Ann Neurol.

[CR379] Parkinson Study Group (2002). A controlled trial of rasagiline in early Parkinson disease: the TEMPO Study. Arch Neurol.

[CR380] Petronilli V, Miotto G, Canton M, Brini M, Colonna R, Bernardi P, Di Lisa F (1999). Transient and long-lasting openings of the mitochondrial permeability transition pore can be monitored directly in intact cells by changes in mitochondrial calcein fluorescence. Biophys J.

[CR381] Petroske E, Meredith GE, Callen S, Totterdell S, Lau YS (2001). Mouse model of Parkinsonism: a comparison between subacute MPTP and chronic MPTP/probenecid treatment. Neuroscience.

[CR382] Petzinger GM, Fisher B, Hogg E, Abernathy A, Arevalo P, Nixon K, Jakowec MW (2006). Behavioral motor recovery in the 1-methyl-4-phenyl-1,2,3,6-tetrahydropyridine-lesioned squirrel monkey (*Saimiri sciureus*): changes in striatal dopamine and expression of tyrosine hydroxylase and dopamine transporter proteins. J Neurosci Res.

[CR383] Piao Y, Kim HG, Oh MS, Pak YK (2012). Overexpression of TFAM, NRF-1 and myr-AKT protects the MPP(+)-induced mitochondrial dysfunctions in neuronal cells. Biochim Biophys Acta.

[CR384] Pickart CM, Cohen RE (2004). Proteasomes and their kin: proteases in the machine age. Nat Rev Mol Cell Biol.

[CR385] Pirker W (2003) Correlation of dopamine transporter imaging with parkinsonian motor handicap: how close is it? Mov Disord 18(Suppl 7):S43–S51 10.1002/mds.10579 14531046

[CR386] Pisanu A, Lecca D, Mulas G, Wardas J, Simbula G, Spiga S, Carta AR (2014). Dynamic changes in pro- and anti-inflammatory cytokines in microglia after PPAR-γ agonist neuroprotective treatment in the MPTPp mouse model of progressive Parkinson’s disease. Neurobiol Dis.

[CR387] Pissadaki EK, Bolam JP (2013). The energy cost of action potential propagation in dopamine neurons: clues to susceptibility in Parkinson’s disease. Front Comput Neurosci.

[CR388] Pivtoraiko VN, Stone SL, Roth KA, Shacka JJ (2009). Oxidative stress and autophagy in the regulation of lysosome-dependent neuron death. Antioxid Redox Signal.

[CR389] Plowey ED, Cherra SJ, Liu YJ, Chu CT (2008). Role of autophagy in G2019S-LRRK2-associated neurite shortening in differentiated SH-SY5Y cells. J Neurochem.

[CR390] Pöltl D, Schildknecht S, Karreman C, Leist M (2012). Uncoupling of ATP-depletion and cell death in human dopaminergic neurons. Neurotoxicology.

[CR391] Porras G, Li Q, Bezard E (2012). Modeling Parkinson’s disease in primates: the MPTP model. Cold Spring Harb Perspect Med.

[CR392] Pott Godoy MC, Tarelli R, Ferrari CC, Sarchi MI, Pitossi FJ (2008). Central and systemic IL-1 exacerbates neurodegeneration and motor symptoms in a model of Parkinson’s disease. Brain.

[CR393] Przedborski S, Kostic V, Jackson-Lewis V, Naini AB, Simonetti S, Fahn S, Carlson E, Epstein CJ, Cadet JL (1992). Transgenic mice with increased Cu/Zn-superoxide dismutase activity are resistant to* N*-methyl-4-phenyl-1,2,3,6-tetrahydropyridine-induced neurotoxicity. J Neurosci.

[CR394] Przedborski S, Jackson-Lewis V, Yokoyama R, Shibata T, Dawson VL, Dawson TM (1996). Role of neuronal nitric oxide in 1-methyl-4-phenyl-1,2,3,6-tetrahydropyridine (MPTP)-induced dopaminergic neurotoxicity. Proc Natl Acad Sci USA.

[CR395] Qin L, Wu X, Block ML, Liu Y, Breese GR, Hong JS, Knapp DJ, Crews FT (2007). Systemic LPS causes chronic neuroinflammation and progressive neurodegeneration. Glia.

[CR396] Qureshi HY, Paudel HK (2011). Parkinsonian neurotoxin 1-methyl-4-phenyl-1,2,3,6-tetrahydropyridine (MPTP) and alpha-synuclein mutations promote Tau protein phosphorylation at Ser262 and destabilize microtubule cytoskeleton in vitro. J Biol Chem.

[CR397] Raff MC, Whitmore AV, Finn JT (2002). Axonal self-destruction and neurodegeneration. Science.

[CR398] Rajapakshe AR, Podyma-Inoue KA, Terasawa K, Hasegawa K, Namba T, Kumei Y, Yanagishita M, Hara-Yokoyama M (2015). Lysosome-associated membrane proteins (LAMPs) regulate intracellular positioning of mitochondria in MC3T3–E1 cells. Exp Cell Res.

[CR399] Rakshi JS, Uema T, Ito K, Bailey DL, Morrish PK, Ashburner J, Dagher A, Jenkins IH, Friston KJ, Brooks DJ (1999). Frontal, midbrain and striatal dopaminergic function in early and advanced Parkinson’s disease A 3D [(18)F]dopa-PET study. Brain.

[CR400] Ramsey CP, Tansey MG (2014). A survey from 2012 of evidence for the role of neuroinflammation in neurotoxin animal models of Parkinson’s disease and potential molecular targets. Exp Neurol.

[CR401] Rascol O, Brooks DJ, Melamed E, Oertel W, Poewe W, Stocchi F, Tolosa E, LARGO study group (2005). Rasagiline as an adjunct to levodopa in patients with Parkinson’s disease and motor fluctuations (LARGO, Lasting effect in Adjunct therapy with Rasagiline Given Once daily, study): a randomised, double-blind, parallel-group trial. Lancet.

[CR402] Rasheed MZ, Tabassum H, Parvez S (2017). Mitochondrial permeability transition pore: a promising target for the treatment of Parkinson’s disease. Protoplasma.

[CR403] Reinheckel T, Sitte N, Ullrich O, Kuckelkorn U, Davies KJ, Grune T (1998) Comparative resistance of the 20S and 26S proteasome to oxidative stress. Biochem J 335 (Pt 3):637–642 10.1042/bj3350637 PMC1219826 9794805

[CR404] Reinheckel T, Ullrich O, Sitte N, Grune T (2000). Differential impairment of 20S and 26S proteasome activities in human hematopoietic K562 cells during oxidative stress. Arch Biochem Biophys.

[CR405] Reynolds AD, Banerjee R, Liu J, Gendelman HE, Mosley RL (2007). Neuroprotective activities of CD4^+^ CD25^+^ regulatory T cells in an animal model of Parkinson’s disease. J Leukoc Biol.

[CR406] Rideout HJ, Larsen KE, Sulzer D, Stefanis L (2001). Proteasomal inhibition leads to formation of ubiquitin/alpha-synuclein-immunoreactive inclusions in PC12 cells. J Neurochem.

[CR407] Rinne JO, Kuikka JT, Bergström KA, Rinne UK (1995). Striatal dopamine transporter in different disability stages of Parkinson’s disease studied with [(123)I]beta-CIT SPECT. Parkinsonism Relat Disord.

[CR408] Robotta M, Gerding HR, Vogel A, Hauser K, Schildknecht S, Karreman C, Leist M, Subramaniam V, Drescher M (2014). Alpha-synuclein binds to the inner membrane of mitochondria in an α-helical conformation. Chembiochem.

[CR409] Rodriguez-Oroz MC, Jahanshahi M, Krack P, Litvan I, Macias R, Bezard E, Obeso JA (2009). Initial clinical manifestations of Parkinson’s disease: features and pathophysiological mechanisms. Lancet Neurol.

[CR410] Rothblat DS, Schroeder JA, Schneider JS (2001). Tyrosine hydroxylase and dopamine transporter expression in residual dopaminergic neurons: potential contributors to spontaneous recovery from experimental Parkinsonism. J Neurosci Res.

[CR411] Roy A, Ghosh A, Jana A, Liu X, Brahmachari S, Gendelman HE, Pahan K (2012). Sodium phenylbutyrate controls neuroinflammatory and antioxidant activities and protects dopaminergic neurons in mouse models of Parkinson’s disease. PLoS One.

[CR412] Rozas G, López-Martín E, Guerra MJ, Labandeira-García JL (1998). The overall rod performance test in the MPTP-treated-mouse model of Parkinsonism. J Neurosci Methods.

[CR413] Ruch W, Cooper PH, Baggiolini M (1983). Assay of H_2_O_2_ production by macrophages and neutrophils with homovanillic acid and horse-radish peroxidase. J Immunol Methods.

[CR414] Saada A, Edvardson S, Rapoport M, Shaag A, Amry K, Miller C, Lorberboum-Galski H, Elpeleg O (2008). C6ORF66 is an assembly factor of mitochondrial complex I. Am J Hum Genet.

[CR415] Saha AR, Hill J, Utton MA, Asuni AA, Ackerley S, Grierson AJ, Miller CC, Davies AM, Buchman VL, Anderton BH, Hanger DP (2004). Parkinson’s disease alpha-synuclein mutations exhibit defective axonal transport in cultured neurons. J Cell Sci.

[CR416] Salabei JK, Gibb AA, Hill BG (2014). Comprehensive measurement of respiratory activity in permeabilized cells using extracellular flux analysis. Nat Protoc.

[CR417] Sanders LH, McCoy J, Hu X, Mastroberardino PG, Dickinson BC, Chang CJ, Chu CT, Van Houten B, Greenamyre JT (2014). Mitochondrial DNA damage: molecular marker of vulnerable nigral neurons in Parkinson’s disease. Neurobiol Dis.

[CR418] von SandströmTobel J, Zoia D, Althaus J, Antinori P, Mermoud J, Pak HS, Scherl A, Monnet-Tschudi F (2014). Immediate and delayed effects of subchronic Paraquat exposure during an early differentiation stage in 3D-rat brain cell cultures. Toxicol Lett.

[CR419] Santoro M, Maetzler W, Stathakos P, Martin HL, Hobert MA, Rattay TW, Gasser T, Forrester JV, Berg D, Tracey KJ, Riedel G, Teismann P (2016). In-vivo evidence that high mobility group box 1 exerts deleterious effects in the 1-methyl-4-phenyl-1,2,3,6-tetrahydropyridine model and Parkinson’s disease which can be attenuated by glycyrrhizin. Neurobiol Dis.

[CR420] Saotome M, Safiulina D, Szabadkai G, Das S, Fransson A, Aspenstrom P, Rizzuto R, Hajnóczky G (2008). Bidirectional Ca^2+^-dependent control of mitochondrial dynamics by the Miro GTPase. Proc Natl Acad Sci USA.

[CR421] Sarkar S, Chigurupati S, Raymick J, Mann D, Bowyer JF, Schmitt T, Beger RD, Hanig JP, Schmued LC, Paule MG (2014). Neuroprotective effect of the chemical chaperone, trehalose in a chronic MPTP-induced Parkinson’s disease mouse model. Neurotoxicology.

[CR422] Sasaki T, Liu K, Agari T, Yasuhara T, Morimoto J, Okazaki M, Takeuchi H, Toyoshima A, Sasada S, Shinko A, Kondo A, Kameda M, Miyazaki I, Asanuma M, Borlongan CV, Nishibori M, Date I (2016) Anti-high mobility group box 1 antibody exerts neuroprotection in a rat model of Parkinson’s disease. Exp Neurol 275(Pt 1):220–231 10.1016/j.expneurol.2015.11.003 26555088

[CR423] Sayre LM, Smith MA, Perry G (2001). Chemistry and biochemistry of oxidative stress in neurodegenerative disease. Curr Med Chem.

[CR424] Schapira AH (2013). Recent developments in biomarkers in Parkinson disease. Curr Opin Neurol.

[CR425] Schierle GS, Hansson O, Leist M, Nicotera P, Widner H, Brundin P (1999). Caspase inhibition reduces apoptosis and increases survival of nigral transplants. Nat Med.

[CR426] Schildknecht S, Bachschmid M, Baumann A, Ullrich V (2004). COX-2 inhibitors selectively block prostacyclin synthesis in endotoxin-exposed vascular smooth muscle cells. FASEB J.

[CR427] Schildknecht S, Bachschmid M, Weber K, Maass D, Ullrich V (2005). Endotoxin elicits nitric oxide release in rat but prostacyclin synthesis in human and bovine vascular smooth muscle cells. Biochem Biophys Res Commun.

[CR428] Schildknecht S, Pöltl D, Nagel DM, Matt F, Scholz D, Lotharius J, Schmieg N, Salvo-Vargas A, Leist M (2009). Requirement of a dopaminergic neuronal phenotype for toxicity of low concentrations of 1-methyl-4-phenylpyridinium to human cells. Toxicol Appl Pharmacol.

[CR429] Schildknecht S, Pape R, Müller N, Robotta M, Marquardt A, Bürkle A, Drescher M, Leist M (2011). Neuroprotection by minocycline caused by direct and specific scavenging of peroxynitrite. J Biol Chem.

[CR430] Schildknecht S, Gerding HR, Karreman C, Drescher M, Lashuel HA, Outeiro TF, Di Monte DA, Leist M (2013). Oxidative and nitrative alpha-synuclein modifications and proteostatic stress: implications for disease mechanisms and interventions in synucleinopathies. J Neurochem.

[CR431] Schildknecht S, Pape R, Meiser J, Karreman C, Strittmatter T, Odermatt M, Cirri E, Friemel A, Ringwald M, Pasquarelli N, Ferger B, Brunner T, Marx A, Möller HM, Hiller K, Leist M (2015) Preferential extracellular generation of the active parkinsonian toxin MPP ^+^ by transporter-independent export of the intermediate MPDP^+^. Antiox Redox Signal 23(13):1001–1016 10.1089/ars.2015.6297 PMC4649766 26413876

[CR432] Schildknecht S, Di Monte DA, Pape R, Tieu K, Leist M (2017). Tipping points and endogenous determinants of nigrostriatal degeneration by MPTP. Trends Pharmacol Sci.

[CR433] Schmidt M, Hanna J, Elsasser S, Finley D (2005). Proteasome-associated proteins: regulation of a proteolytic machine. Biol Chem.

[CR434] Schmued LC, Albertson C, Slikker W (1997). Fluoro-Jade: a novel fluorochrome for the sensitive and reliable histochemical localization of neuronal degeneration. Brain Res.

[CR435] Scholz D, Pöltl D, Genewsky A, Weng M, Waldmann T, Schildknecht S, Leist M (2011). Rapid, complete and large-scale generation of post-mitotic neurons from the human LUHMES cell line. J Neurochem.

[CR436] Schumacher JM, Ellias SA, Palmer EP, Kott HS, Dinsmore J, Dempsey PK, Fischman AJ, Thomas C, Feldman RG, Kassissieh S, Raineri R, Manhart C, Penney D, Fink JS, Isacson O (2000). Transplantation of embryonic porcine mesencephalic tissue in patients with PD. Neurology.

[CR437] Seniuk NA, Tatton WG, Greenwood CE (1990). Dose-dependent destruction of the coeruleus-cortical and nigral-striatal projections by MPTP. Brain Res.

[CR438] Seo BB, Kitajima-Ihara T, Chan EK, Scheffler IE, Matsuno-Yagi A, Yagi T (1998). Molecular remedy of complex I defects: rotenone-insensitive internal NADH-quinone oxidoreductase of* Saccharomyces cerevisiae* mitochondria restores the NADH oxidase activity of complex I-deficient mammalian cells. Proc Natl Acad Sci USA.

[CR439] Seo BB, Wang J, Flotte TR, Yagi T, Matsuno-Yagi A (2000). Use of the NADH-quinone oxidoreductase (NDI1) gene of* Saccharomyces cerevisiae* as a possible cure for complex I defects in human cells. J Biol Chem.

[CR440] Seo BB, Nakamaru-Ogiso E, Flotte TR, Yagi T, Matsuno-Yagi A (2002). A single-subunit NADH-quinone oxidoreductase renders resistance to mammalian nerve cells against complex I inhibition. Mol Ther.

[CR441] Shacka JJ, Roth KA, Zhang J (2008). The autophagy-lysosomal degradation pathway: role in neurodegenerative disease and therapy. Front Biosci.

[CR442] Shamoto-Nagai M, Maruyama W, Kato Y, Isobe K, Tanaka M, Naoi M, Osawa T (2003). An inhibitor of mitochondrial complex I, rotenone, inactivates proteasome by oxidative modification and induces aggregation of oxidized proteins in SH-SY5Y cells. J Neurosci Res.

[CR443] Shan S, Hong-Min T, Yi F, Jun-Peng G, Yue F, Yan-Hong T, Yun-Ke Y, Wen-Wei L, Xiang-Yu W, Jun M, Guo-Hua W, Ya-Ling H, Hua-Wei L, Ding-Fang C (2011). New evidences for fractalkine/CX3CL1 involved in substantia nigral microglial activation and behavioral changes in a rat model of Parkinson’s disease. Neurobiol Aging.

[CR444] Sharma LK, Lu J, Bai Y (2009). Mitochondrial respiratory complex I: structure, function and implication in human diseases. Curr Med Chem.

[CR445] Shaw VE, Keay KA, Ashkan K, Benabid AL, Mitrofanis J (2010). Dopaminergic cells in the periaqueductal grey matter of MPTP-treated monkeys and mice; patterns of survival and effect of deep brain stimulation and lesion of the subthalamic nucleus. Parkinsonism Relat Disord.

[CR446] Sheehan JP, Swerdlow RH, Parker WD, Miller SW, Davis RE, Tuttle JB (1997). Altered calcium homeostasis in cells transformed by mitochondria from individuals with Parkinson’s disease. J Neurochem.

[CR447] Sherer TB, Betarbet R, Stout AK, Lund S, Baptista M, Panov AV, Cookson MR, Greenamyre JT (2002). An in vitro model of Parkinson’s disease: linking mitochondrial impairment to altered alpha-synuclein metabolism and oxidative damage. J Neurosci.

[CR448] Sherer TB, Betarbet R, Testa CM, Seo BB, Richardson JR, Kim JH, Miller GW, Yagi T, Matsuno-Yagi A, Greenamyre JT (2003). Mechanism of toxicity in rotenone models of Parkinson’s disease. J Neurosci.

[CR449] Sherer TB, Richardson JR, Testa CM, Seo BB, Panov AV, Yagi T, Matsuno-Yagi A, Miller GW, Greenamyre JT (2007). Mechanism of toxicity of pesticides acting at complex I: relevance to environmental etiologies of Parkinson’s disease. J Neurochem.

[CR450] Shimoji M, Zhang L, Mandir AS, Dawson VL, Dawson TM (2005). Absence of inclusion body formation in the MPTP mouse model of Parkinson’s disease. Brain Res Mol Brain Res.

[CR451] Shimomura Y, Kawada T, Suzuki M (1989). Capsaicin and its analogs inhibit the activity of NADH-coenzyme Q oxidoreductase of the mitochondrial respiratory chain. Arch Biochem Biophys.

[CR452] Shin JY, Park HJ, Ahn YH, Lee PH (2009). Neuroprotective effect of L-dopa on dopaminergic neurons is comparable to pramipexol in MPTP-treated animal model of Parkinson’s disease: a direct comparison study. J Neurochem.

[CR453] Shinozaki Y, Shibata K, Yoshida K, Shigetomi E, Gachet C, Ikenaka K, Tanaka KF, Koizumi S (2017). Transformation of astrocytes to a neuroprotective phenotype by microglia via P2Y1 receptor downregulation. Cell Rep.

[CR454] Shoulson I (1998). DATATOP: a decade of neuroprotective inquiry. Parkinson Study Group. Deprenyl And Tocopherol Antioxidative Therapy Of Parkinsonism. Ann Neurol.

[CR455] Shults CW (2003). Coenzyme Q10 in neurodegenerative diseases. Curr Med Chem.

[CR456] Shults CW, Haas RH, Passov D, Beal MF (1997). Coenzyme Q10 levels correlate with the activities of complexes I and II/III in mitochondria from parkinsonian and nonparkinsonian subjects. Ann Neurol.

[CR457] Shults CW, Haas RH, Beal MF (1999). A possible role of coenzyme Q10 in the etiology and treatment of Parkinson’s disease. Biofactors.

[CR458] Shults CW, Oakes D, Kieburtz K, Beal MF, Haas R, Plumb S, Juncos JL, Nutt J, Shoulson I, Carter J, Kompoliti K, Perlmutter JS, Reich S, Stern M, Watts RL, Kurlan R, Molho E, Harrison M, Lew M, Parkinson Study Group (2002). Effects of coenzyme Q10 in early Parkinson disease: evidence of slowing of the functional decline. Arch Neurol.

[CR459] Shults CW, Flint Beal M, Song D, Fontaine D (2004). Pilot trial of high dosages of coenzyme Q10 in patients with Parkinson’s disease. Exp Neurol.

[CR460] Sieger D, Moritz C, Ziegenhals T, Prykhozhij S, Peri F (2012). Long-range Ca^2+^ waves transmit brain-damage signals to microglia. Dev Cell.

[CR461] Silverdale MA, Fox SH, Crossman AR, Brotchie JM (2003). Potential nondopaminergic drugs for Parkinson’s disease. Adv Neurol.

[CR462] Singer TP (1979). Mitochondrial electron-transport inhibitors. Methods Enzymol.

[CR463] Singleton AB, Farrer M, Johnson J, Singleton A, Hague S, Kachergus J, Hulihan M, Peuralinna T, Dutra A, Nussbaum R, Lincoln S, Crawley A, Hanson M, Maraganore D, Adler C, Cookson MR, Muenter M, Baptista M, Miller D, Blancato J, Hardy J, Gwinn-Hardy K (2003). alpha-Synuclein locus triplication causes Parkinson’s disease. Science.

[CR464] Smith Y, Kieval JZ (2000). Anatomy of the dopamine system in the basal ganglia. Trends Neurosci.

[CR465] Smith Y, Bennett BD, Bolam JP, Parent A, Sadikot AF (1994). Synaptic relationships between dopaminergic afferents and cortical or thalamic input in the sensorimotor territory of the striatum in monkey. J Comp Neurol.

[CR466] Smith LA, Jackson MJ, Hansard MJ, Maratos E, Jenner P (2003). Effect of pulsatile administration of levodopa on dyskinesia induction in drug-naïve MPTP-treated common marmosets: effect of dose, frequency of administration, and brain exposure. Mov Disord.

[CR467] Snow BJ, Vingerhoets FJ, Langston JW, Tetrud JW, Sossi V, Calne DB (2000). Pattern of dopaminergic loss in the striatum of humans with MPTP induced parkinsonism. J Neurol Neurosurg Psychiatry.

[CR468] Song L, Cortopassi G (2015). Mitochondrial complex I defects increase ubiquitin in substantia nigra. Brain Res.

[CR469] Spillantini MG, Schmidt ML, Lee VM, Trojanowski JQ, Jakes R, Goedert M (1997). Alpha-synuclein in Lewy bodies. Nature.

[CR470] Spinazzi M, Casarin A, Pertegato V, Salviati L, Angelini C (2012). Assessment of mitochondrial respiratory chain enzymatic activities on tissues and cultured cells. Nat Protoc.

[CR471] Sriram K, Matheson JM, Benkovic SA, Miller DB, Luster MI, O’Callaghan JP (2002). Mice deficient in TNF receptors are protected against dopaminergic neurotoxicity: implications for Parkinson’s disease. FASEB J.

[CR472] Sriram K, Matheson JM, Benkovic SA, Miller DB, Luster MI, O’Callaghan JP (2006). Deficiency of TNF receptors suppresses microglial activation and alters the susceptibility of brain regions to MPTP-induced neurotoxicity: role of TNF-alpha. FASEB J.

[CR473] Stefanis L, Larsen KE, Rideout HJ, Sulzer D, Greene LA (2001). Expression of A53T mutant but not wild-type alpha-synuclein in PC12 cells induces alterations of the ubiquitin-dependent degradation system, loss of dopamine release, and autophagic cell death. J Neurosci.

[CR474] Stone DK, Reynolds AD, Mosley RL, Gendelman HE (2009). Innate and adaptive immunity for the pathobiology of Parkinson’s disease. Antioxid Redox Signal.

[CR475] Streit WJ, Walter SA, Pennell NA (1999). Reactive microgliosis. Prog Neurobiol.

[CR476] Streit WJ, Conde JR, Harrison JK (2001). Chemokines and Alzheimer’s disease. Neurobiol Aging.

[CR477] Struzynska L, Dabrowska-Bouta B, Koza K, Sulkowski G (2007). Inflammation-like glial response in lead-exposed immature rat brain. Toxicol Sci.

[CR478] Subramaniam SR, Chesselet MF (2013). Mitochondrial dysfunction and oxidative stress in Parkinson’s disease. Prog Neurobiol.

[CR479] Sulzer D, Zecca L (2000). Intraneuronal dopamine-quinone synthesis: a review. Neurotox Res.

[CR480] Surmeier DJ, Schumacker PT (2013). Calcium, bioenergetics, and neuronal vulnerability in Parkinson’s disease. J Biol Chem.

[CR481] Surmeier DJ, Guzman JN, Sanchez-Padilla J, Schumacker PT (2011) The role of calcium and mitochondrial oxidant stress in the loss of substantia nigra pars compacta dopaminergic neurons in Parkinson’s disease. Neuroscience 198:221–231 10.1016/j.neuroscience.2011.08.045 PMC3244353 21884755

[CR482] Suzuki H, King TE (1983). Evidence of an ubisemiquinone radical(s) from the NADH-ubiquinone reductase of the mitochondrial respiratory chain. J Biol Chem.

[CR483] Talpade DJ, Greene JG, Higgins DS, Greenamyre JT (2000). In vivo labeling of mitochondrial complex I (NADH:ubiquinone oxidoreductase) in rat brain using [(3)H]dihydrorotenone. J Neurochem.

[CR484] Tanaka S, Ishii A, Ohtaki H, Shioda S, Yoshida T, Numazawa S (2013). Activation of microglia induces symptoms of Parkinson’s disease in wild-type, but not in IL-1 knockout mice. J Neuroinflammation.

[CR485] Tanner CM, Kamel F, Ross GW, Hoppin JA, Goldman SM, Korell M, Marras C, Bhudhikanok GS, Kasten M, Chade AR, Comyns K, Richards MB, Meng C, Priestley B, Fernandez HH, Cambi F, Umbach DM, Blair A, Sandler DP, Langston JW (2011). Rotenone, paraquat, and Parkinson’s disease. Environ Health Perspect.

[CR486] Thomas B, Banerjee R, Starkova NN, Zhang SF, Calingasan NY, Yang L, Wille E, Lorenzo BJ, Ho DJ, Beal MF, Starkov A (2012). Mitochondrial permeability transition pore component cyclophilin D distinguishes nigrostriatal dopaminergic death paradigms in the MPTP mouse model of Parkinson’s disease. Antioxid Redox Signal.

[CR487] Thundyil J, Lim KL (2015). DAMPs and neurodegeneration. Ageing Res Rev.

[CR488] Tieu K (2011). A guide to neurotoxic animal models of Parkinson’s disease. Cold Spring Harb Perspect Med.

[CR489] Tikka T, Fiebich BL, Goldsteins G, Keinanen R, Koistinaho J (2001). Minocycline, a tetracycline derivative, is neuroprotective against excitotoxicity by inhibiting activation and proliferation of microglia. J Neurosci.

[CR490] Tillerson JL, Miller GW (2002). Forced limb-use and recovery following brain injury. Neuroscientist.

[CR491] Tissingh G, Bergmans P, Booij J, Winogrodzka A, van Royen EA, Stoof JC, Wolters EC (1998). Drug-naive patients with Parkinson’s disease in Hoehn and Yahr stages I and II show a bilateral decrease in striatal dopamine transporters as revealed by [^123^I]beta-CIT SPECT. J Neurol.

[CR492] Ton TG, Heckbert SR, Longstreth WT, Rossing MA, Kukull WA, Franklin GM, Swanson PD, Smith-Weller T, Checkoway H (2006). Nonsteroidal anti-inflammatory drugs and risk of Parkinson’s disease. Mov Disord.

[CR493] Tong J, Boileau I, Furukawa Y, Chang LJ, Wilson AA, Houle S, Kish SJ (2011). Distribution of vesicular monoamine transporter 2 protein in human brain: implications for brain imaging studies. J Cereb Blood Flow Metab.

[CR494] Toulorge D, Schapira AH, Hajj R (2016). Molecular changes in the postmortem parkinsonian brain. J Neurochem 139 Suppl.

[CR495] Treberg JR, Brand MD (2011). A model of the proton translocation mechanism of complex I. J Biol Chem.

[CR496] Tseng YT, Chang FR, Lo YC (2014). The Chinese herbal formula Liuwei dihuang protects dopaminergic neurons against Parkinson’s toxin through enhancing antioxidative defense and preventing apoptotic death. Phytomedicine.

[CR497] Ungerstedt U, Arbuthnott GW (1970). Quantitative recording of rotational behavior in rats after 6-hydroxy-dopamine lesions of the nigrostriatal dopamine system. Brain Res.

[CR498] van Belzen R, Kotlyar AB, Moon N, Dunham WR, Albracht SP (1997). The iron-sulfur clusters 2 and ubisemiquinone radicals of NADH:ubiquinone oxidoreductase are involved in energy coupling in submitochondrial particles. Biochemistry.

[CR499] Van Maele-Fabry G, Hoet P, Vilain F, Lison D (2012). Occupational exposure to pesticides and Parkinson’s disease: a systematic review and meta-analysis of cohort studies. Environ Int.

[CR500] Varastet M, Riche D, Maziere M, Hantraye P (1994). Chronic MPTP treatment reproduces in baboons the differential vulnerability of mesencephalic dopaminergic neurons observed in Parkinson’s disease. Neuroscience.

[CR501] Villarán RF, Espinosa-Oliva AM, Sarmiento M, De Pablos RM, Argüelles S, Delgado-Cortés MJ, Sobrino V, Van Rooijen N, Venero JL, Herrera AJ, Cano J, Machado A (2010). Ulcerative colitis exacerbates lipopolysaccharide-induced damage to the nigral dopaminergic system: potential risk factor in Parkinson’s disease. J Neurochem.

[CR502] Villeneuve DL, Crump D, Garcia-Reyero N, Hecker M, Hutchinson TH, LaLone CA, Landesmann B, Lettieri T, Munn S, Nepelska M, Ottinger MA, Vergauwen L, Whelan M (2014). Adverse outcome pathway (AOP) development I: strategies and principles. Toxicol Sci.

[CR503] Villeneuve DL, Crump D, Garcia-Reyero N, Hecker M, Hutchinson TH, LaLone CA, Landesmann B, Lettieri T, Munn S, Nepelska M, Ottinger MA, Vergauwen L, Whelan M (2014). Adverse outcome pathway development II: best practices. Toxicol Sci.

[CR504] Vinogradov AD (1993). Kinetics, control, and mechanism of ubiquinone reduction by the mammalian respiratory chain-linked NADH-ubiquinone reductase. J Bioenerg Biomembr.

[CR505] Vinogradov AD, Sled VD, Burbaev DS, Grivennikova VG, Moroz IA, Ohnishi T (1995). Energy-dependent Complex I-associated ubisemiquinones in submitochondrial particles. FEBS Lett.

[CR506] Vivekanantham S, Shah S, Dewji R, Dewji A, Khatri C, Ologunde R (2015). Neuroinflammation in Parkinson’s disease: role in neurodegeneration and tissue repair. Int J Neurosci.

[CR507] Voges D, Zwickl P, Baumeister W (1999). The 26S proteasome: a molecular machine designed for controlled proteolysis. Annu Rev Biochem.

[CR508] von Tobel JS, Antinori P, Zurich MG, Rosset R, Aschner M, Glück F, Scherl A, Monnet-Tschudi F (2014). Repeated exposure to Ochratoxin A generates a neuroinflammatory response, characterized by neurodegenerative M1 microglial phenotype. Neurotoxicology.

[CR510] Wang XD, Wolfbeis OS (2014). Optical methods for sensing and imaging oxygen: materials, spectroscopies and applications. Chem Soc Rev.

[CR511] Wang XF, Li S, Chou AP, Bronstein JM (2006). Inhibitory effects of pesticides on proteasome activity: implication in Parkinson’s disease. Neurobiol Dis.

[CR512] Wang X, Yen J, Kaiser P, Huang L (2010). Regulation of the 26S proteasome complex during oxidative stress. Sci Signal.

[CR513] Wang X, Su B, Liu W, He X, Gao Y, Castellani RJ, Perry G, Smith MA, Zhu X (2011). DLP1-dependent mitochondrial fragmentation mediates 1-methyl-4-phenylpyridinium toxicity in neurons: implications for Parkinson’s disease. Aging Cell.

[CR514] Wang X, Petrie TG, Liu Y, Liu J, Fujioka H, Zhu X (2012). Parkinson’s disease-associated DJ-1 mutations impair mitochondrial dynamics and cause mitochondrial dysfunction. J Neurochem.

[CR515] Wang S, He H, Chen L, Zhang W, Zhang X, Chen J (2015). Protective effects of salidroside in the MPTP/MPP(+)-induced model of Parkinson’s disease through ROS-NO-related mitochondrion pathway. Mol Neurobiol.

[CR516] Wen Y, Li W, Poteet EC, Xie L, Tan C, Yan LJ, Ju X, Liu R, Qian H, Marvin MA, Goldberg MS, She H, Mao Z, Simpkins JW, Yang SH (2011). Alternative mitochondrial electron transfer as a novel strategy for neuroprotection. J Biol Chem.

[CR517] Widner H, Tetrud J, Rehncrona S, Snow B, Brundin P, Gustavii B, Björklund A, Lindvall O, Langston JW (1992). Bilateral fetal mesencephalic grafting in two patients with parkinsonism induced by 1-methyl-4-phenyl-1,2,3,6-tetrahydropyridine (MPTP). N Engl J Med.

[CR518] Willems PH, Valsecchi F, Distelmaier F, Verkaart S, Visch HJ, Smeitink JA, Koopman WJ (2008). Mitochondrial Ca^2+^ homeostasis in human NADH:ubiquinone oxidoreductase deficiency. Cell Calcium.

[CR519] Willems PH, Smeitink JA, Koopman WJ (2009). Mitochondrial dynamics in human NADH:ubiquinone oxidoreductase deficiency. Int J Biochem Cell Biol.

[CR520] Wirth C, Brandt U, Hunte C, Zickermann V (2016). Structure and function of mitochondrial complex I. Biochim Biophys Acta.

[CR521] Wittwehr C, Aladjov H, Ankley G, Bryne H, de Knecht J, Heinzle E, Klambauer G, Landesmann B, Luijten M, MacKay C, Maxwell G, Meek B, Paini A, Perkins E, Sobanski T, Villeneuve D, Waters K, Whelan M (2017). How adverse outcome pathways can aid the development of computational prediction models for regulatory toxicology. Toxicol Sci.

[CR522] Wu DC, Jackson-Lewis V, Vila M, Tieu K, Teismann P, Vadseth C, Choi DK, Ischiropoulos H, Przedborski S (2002). Blockade of microglial activation is neuroprotective in the 1-methyl-4-phenyl-1,2,3,6-tetrahydropyridine mouse model of Parkinson disease. J Neurosci.

[CR523] Wu F, Poon WS, Lu G, Wang A, Meng H, Feng L, Li Z, Liu S (2009). Alpha-synuclein knockdown attenuates MPP + induced mitochondrial dysfunction of SH-SY5Y cells. Brain Res.

[CR524] Wu F, Xu HD, Guan JJ, Hou YS, Gu JH, Zhen XC, Qin ZH (2015). Rotenone impairs autophagic flux and lysosomal functions in Parkinson’s disease. Neuroscience.

[CR525] Xie W, Chung KK (2012). Alpha-synuclein impairs normal dynamics of mitochondria in cell and animal models of Parkinson’s disease. J Neurochem.

[CR526] Yam PS, Patterson J, Graham DI, Takasago T, Dewar D, McCulloch J (1998). Topographical and quantitative assessment of white matter injury following a focal ischaemic lesion in the rat brain. Brain Res Brain Res Protoc.

[CR527] Yang L, Calingasan NY, Wille EJ, Cormier K, Smith K, Ferrante RJ, Beal MF (2009). Combination therapy with coenzyme Q10 and creatine produces additive neuroprotective effects in models of Parkinson’s and Huntington’s diseases. J Neurochem.

[CR528] Ye X, Han Y, Zhang L, Liu W, Zuo J (2015). MTERF4 regulates the mitochondrial dysfunction induced by MPP(+) in SH-SY5Y cells. Biochem Biophys Res Commun.

[CR529] Yi M, Weaver D, Hajnóczky G (2004). Control of mitochondrial motility and distribution by the calcium signal: a homeostatic circuit. J Cell Biol.

[CR530] Yong-Kee CJ, Sidorova E, Hanif A, Perera G, Nash JE (2012). Mitochondrial dysfunction precedes other sub-cellular abnormalities in an in vitro model linked with cell death in Parkinson’s disease. Neurotox Res.

[CR531] Yu WH, Dorado B, Figueroa HY, Wang L, Planel E, Cookson MR, Clark LN, Duff KE (2009). Metabolic activity determines efficacy of macroautophagic clearance of pathological oligomeric alpha-synuclein. Am J Pathol.

[CR532] Yuan H, Zhang ZW, Liang LW, Shen Q, Wang XD, Ren SM, Ma HJ, Jiao SJ, Liu P (2010). Treatment strategies for Parkinson’s disease. Neurosci Bull.

[CR533] Yuan YH, Yan WF, Sun JD, Huang JY, Mu Z, Chen NH (2015). The molecular mechanism of rotenone-induced α-synuclein aggregation: emphasizing the role of the calcium/GSK3β pathway. Toxicol Lett.

[CR534] Zecca L, Wilms H, Geick S, Claasen JH, Brandenburg LO, Holzknecht C, Panizza ML, Zucca FA, Deuschl G, Sievers J, Lucius R (2008). Human neuromelanin induces neuroinflammation and neurodegeneration in the rat substantia nigra: implications for Parkinson’s disease. Acta Neuropathol.

[CR535] Zhang J, Graham DG, Montine TJ, Ho YS (2000). Enhanced* N*-methyl-4-phenyl-1,2,3,6-tetrahydropyridine toxicity in mice deficient in CuZn-superoxide dismutase or glutathione peroxidase. J Neuropathol Exp Neurol.

[CR536] Zharikov AD, Cannon JR, Tapias V, Bai Q, Horowitz MP, Shah V, El Ayadi A, Hastings TG, Greenamyre JT, Burton EA (2015). shRNA targeting α-synuclein prevents neurodegeneration in a Parkinson’s disease model. J Clin Investig.

[CR537] Zheng Q, Huang T, Zhang L, Zhou Y, Luo H, Xu H, Wang X (2016). Dysregulation of ubiquitin-proteasome system in neurodegenerative diseases. Front Aging Neurosci.

[CR538] Zhou M, Diwu Z, Panchuk-Voloshina N, Haugland RP (1997). A stable nonfluorescent derivative of resorufin for the fluorometric determination of trace hydrogen peroxide: applications in detecting the activity of phagocyte NADPH oxidase and other oxidases. Anal Biochem.

[CR539] Zhu JH, Horbinski C, Guo F, Watkins S, Uchiyama Y, Chu CT (2007). Regulation of autophagy by extracellular signal-regulated protein kinases during 1-methyl-4-phenylpyridinium-induced cell death. Am J Pathol.

[CR540] Zhu W, Xie W, Pan T, Xu P, Fridkin M, Zheng H, Jankovic J, Youdim MB, Le W (2007). Prevention and restoration of lactacystin-induced nigrostriatal dopamine neuron degeneration by novel brain-permeable iron chelators. FASEB J.

